# Supplementary material for: FIP200 Claw Domain Binding to p62 Promotes Autophagosome Formation at Ubiquitin Condensates
Source: Mol Cell. 2019 Apr 18;74(2):330–346.e11. doi: 10.1016/j.molcel.2019.01.035 (PMC6477179; doi:10.1016/j.molcel.2019.01.035)
Supplement: Document S1. Figures S1–S7 and Table S1 [file mmc1.pdf]

Molecular Cell, Volume *74*

Supplemental Information

FIP200 Claw Domain Binding to p62 Promotes Autophagosome Formation at Ubiquitin Condensates

Eleonora Turco, Marie Witt, Christine Abert, Tobias Bock-Bierbaum, Ming-Yuan Su, Riccardo Trapannone, Martin Sztacho, Alberto Danieli, Xiaoshan Shi, Gabriele Zaffagnini, Annamaria Gamper, Martina Schuschnig, Dorotea Fracchiolla, Daniel Bernklau, Julia Romanov, Markus Hartl, James H. Hurley, Oliver Daumke, and Sascha Martens

Figure S1 - related to Fig. 1

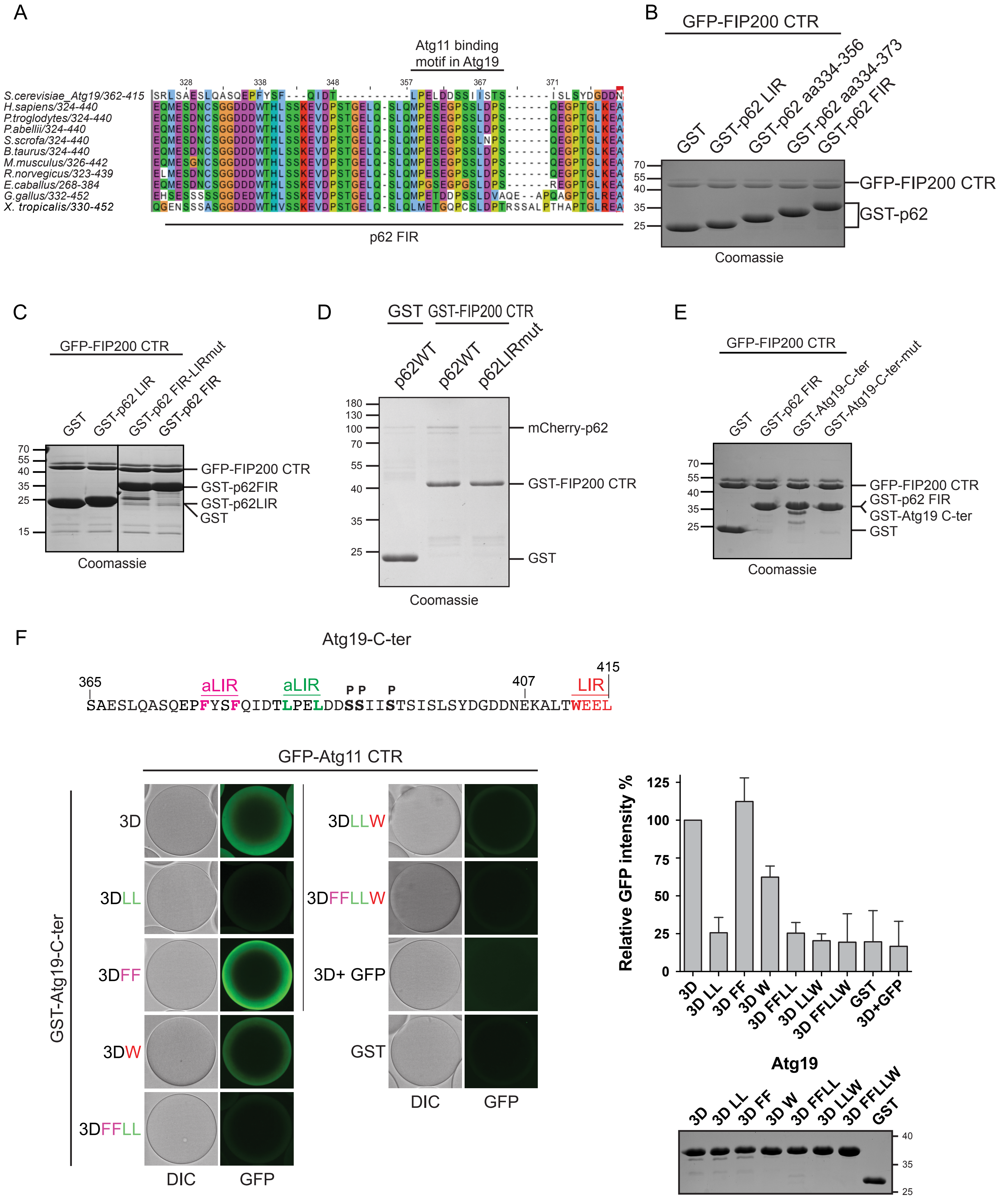

Figure S1, related to Figure 1.

A) Sequence alignment of p62 FIR from different species with *S. cerevisiae* Atg19. The region of p62 showing sequence similarity to the Atg11 binding site in Atg19 (aa 358-370) is indicated above the alignment.

B,C,D,E) Protein inputs for the respective experiments in Fig. 1E, F, G, H. 20 µl of each sample (beads + supernatant) were collected after microscopy imaging and analyzed by SDS-PAGE followed by Coomassie staining.

F) The Atg19 C-terminus sequence is shown. The canonical LIR motif at the extreme C-terminus is labeled in red. Pink and green labels indicate two accessory LIR-like motifs: $_{376}$FYSF$_{379}$ and $_{384}$LPEL$_{387}$. Phosphorylation sites are indicated in bold. The GFP-Atg11 CTR (aa851-1178) was mixed at 1µM final concentration with glutathione beads pre-coated with GST-Atg19 C-terminus (the phosphorylation-mimicking 3D mutant: S390D, S391D, S394D (Pfaffenwimmer et al., 2014) with the indicated LIR mutations (LL: L384, L386A, FF: F376, F379A, W: W412A) and the beads were imaged using a Zeiss LSM700 confocal microscope. The graph on the right shows average intensities (normalized to the signal of GFP-Atg11 CTR bound to GST-Atg19 C-ter 3D) and standard deviation of 3 independent experiments. The gel below the graph shows beads bound GST/GST Atg19 C-terminus constructs (1µl of each beads sample was loaded on the gel).

Figure S2 - related to Fig. 2

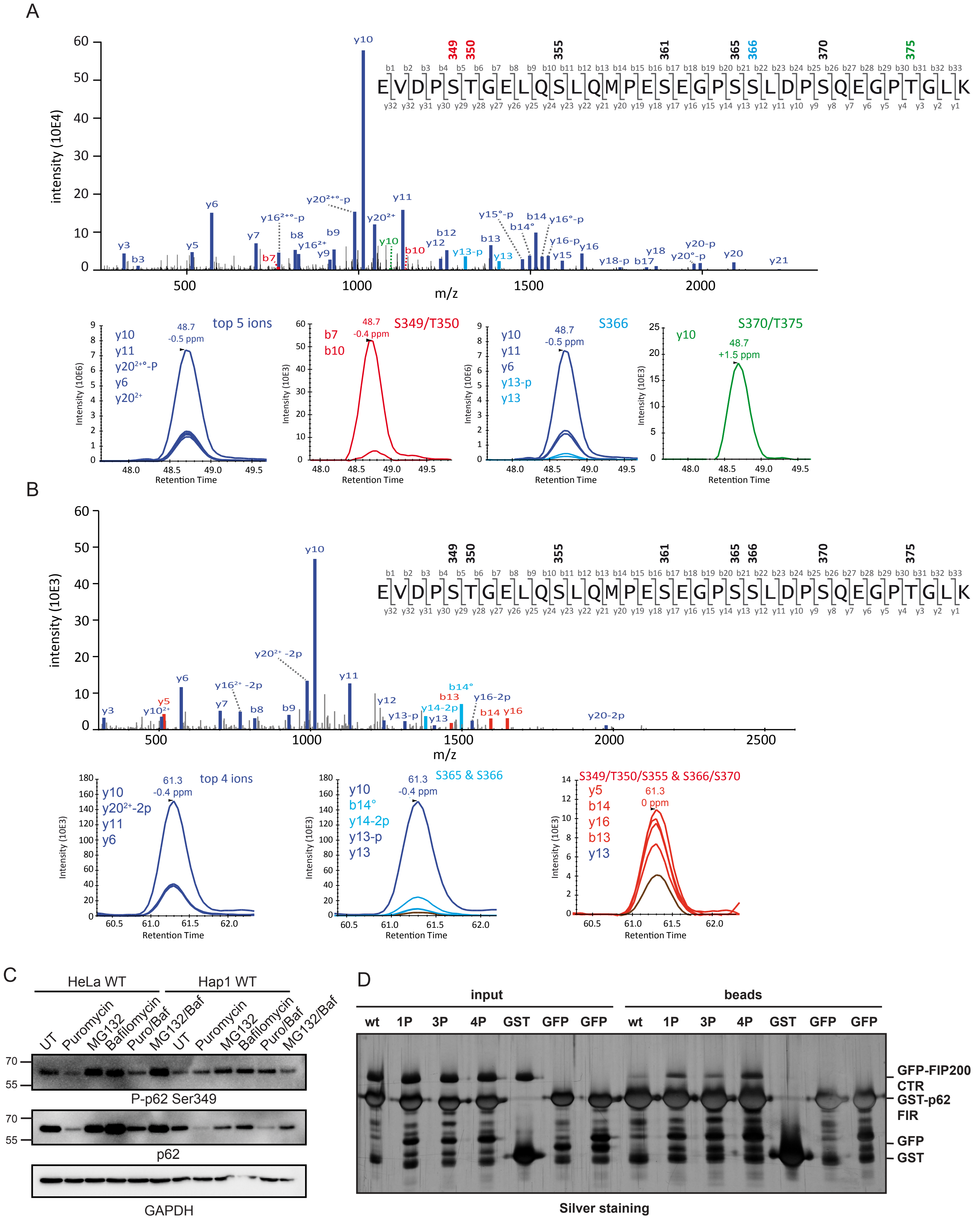

Figure S2, related to Figure 2.

A, B) MS/MS fragmentation spectra of singly (A) and doubly (B) phosphorylated forms of the peptide covering the FIR of p62. Spectra were acquired using parallel reaction monitoring, targeting peptide forms with up to four phosphorylations. Since all singly phosphorylated variants co-eluted, the spectra obtained display mixed signatures of different phosphorylation sites. Although this makes an unambiguous designation for some of the potential sites impossible (S355, S361), a combined analysis with the data of the doubly phosphorylated peptide allowed a clear assignment for two sites (S365, S366). Additionally, there is evidence for phosphorylation of at least one or both of the neighboring sites at the positions S349/T350, as well as at S370/S375. Depicted spectra were summed over the whole chromatographic peak, ions are color-coded for site-specific and -unspecific fragment ions, and the corresponding ion chromatograms are shown below each spectrum. Selected ions were validated manually in terms of mass accuracy, co-elution, isotopic envelope, and charge state. Dark blue: fragment ions with ambiguous site information; light blue, red, green: site-specific fragment ions; p: neutral phosphate loss; °: water loss.

C) HeLa and HAP1 cells were treated with puromycin, MG132, Bafilomycin or a combination of them for 3 h and cell lysates were analyzed by Western blot. The membranes were probed with a phospho-S349 specific antibody (top) or with an anti-p62 antibody (middle). An anti-GAPDH antibody was used as a loading control (bottom).

D) Binding of GFP-FIP200 CTR to GST-p62 FIR phospho-mimicking mutant assessed by GST pull-down. The same beads analyzed by microscopy in Fig. 2B were loaded on the gel before being washed (inputs) and after the unbound protein was washed away (beads).

Figure S3 - related to Fig. 2 and 3

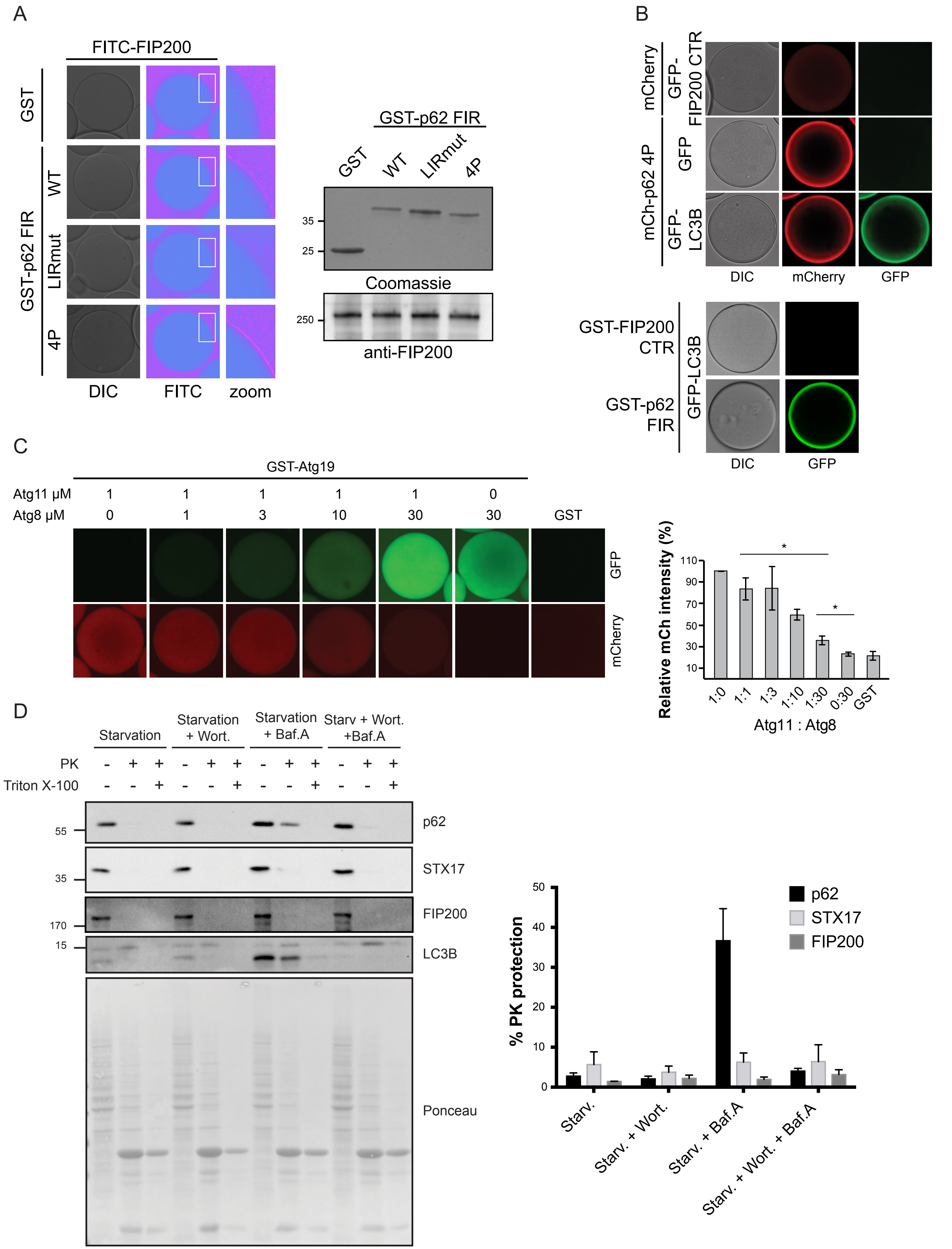

Figure S3, related to Figure 2 and 3.

A) Binding of GST-p62 FIR (wt, LIRmut and 4P phospho-mimetic mutant) to FITC-labelled full length FIP200 was assessed with the same experimental set up as in Fig. 2B. FITC peptide signal is shown in false color (ImageJ: Ice). The amount of bait protein on the beads was visualized by Coomassie staining and FIP200 protein inputs by Western blotting.

B) Negative and positive controls for the experiment in Fig. 3A. In the top panel, mCherry coated beads were incubated with GFP-FIP200 CTR (1.1 μM; top) and GST-p62 FIR 4P coated beads were incubated with GFP only (1.1 μM; middle) or GFP-LC3B (1.1 μM; bottom). In the bottom panel, GST-FIP200 CTR coated beads and GST-p62 FIR coated beads were incubated with GFP-LC3B (2 μM).

C) GST- Atg19 C-terminus coated glutathione beads were pre-incubated with mCherry-tagged Atg11 CTR and increasing amounts of GFP-Atg8 were added. Concentrations of mCherry-Atg11 CTR and GFP-Atg8 are indicated above the images. For the GST negative control, 1 μM mCherry-Atg11 CTR and 1 μM GFP-Atg8 were added. Average mCherry intensity (normalized to the intensity of 1:0 Atg11:Atg8 sample) and standard error of 3 independent experiments are plotted on the right. Significant differences are indicated with * when p value % 0.05, ** when p value % 0.01, and *** when p value % 0.001.

D) Protease protection assay as in Fig. 3B. As additional control, cells were treated with the PI3K inhibitor Wortmannin, which prevents autophagosome formation.

Figure S4 - related to Fig. 4

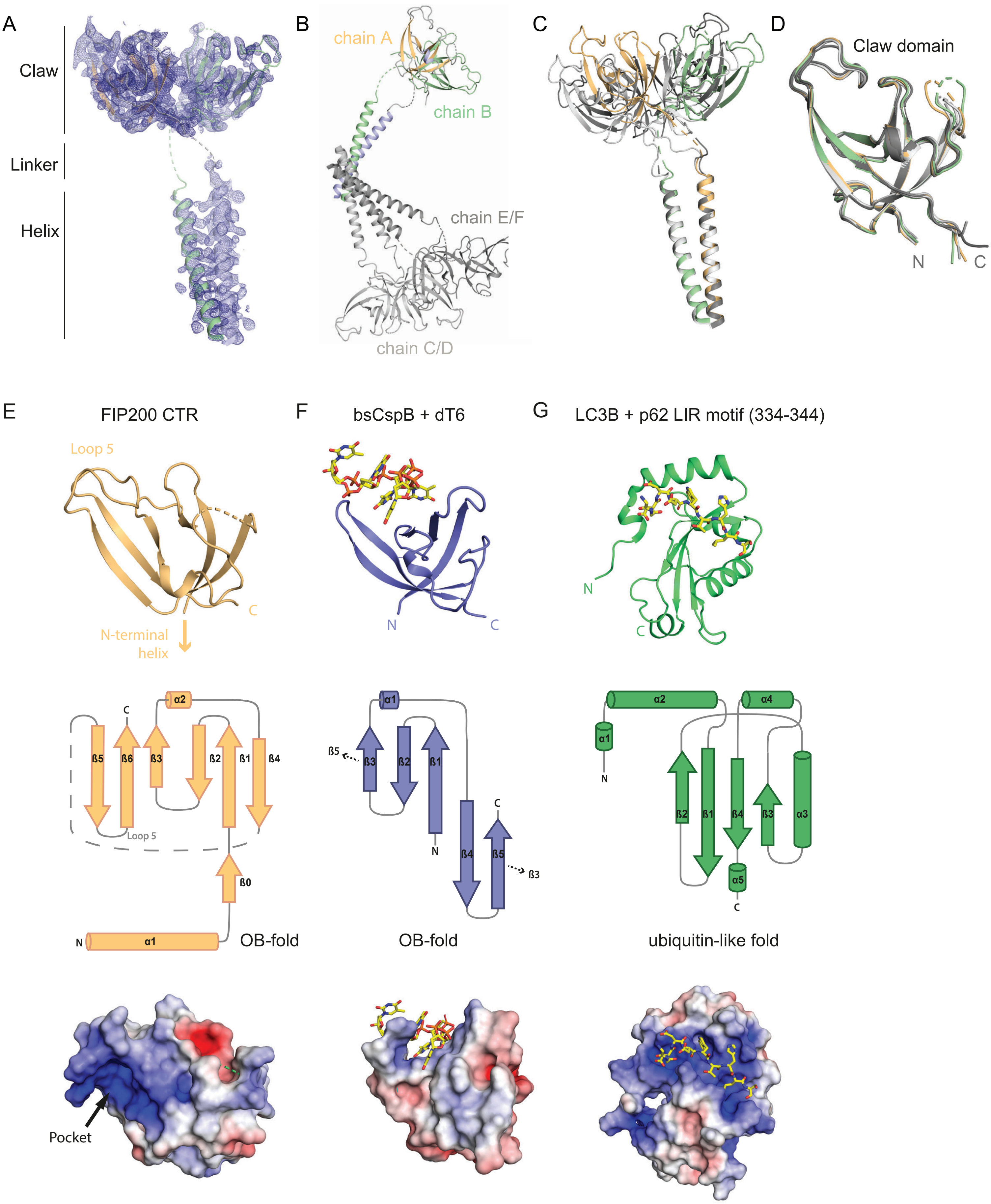

Figure S4, related to Figure 4.

A) Experimental electron density after density modification at 1 σ. Final model shown in cartoon representation.

B) Asymmetric unit cell of the crystal containing six molecules arranged as three dimers. One dimer is shown in colors.

C) Cα atoms superposition of the three dimers of the asymmetric unit cell (orange/green, light gray, dark gray). Only N-terminal α-helices were used for alignment.

D) Superposition of the Cα atoms in the six Claw domains present in the asymmetric unit cell (rmsd = 0.33).

E-G) Comparison of FIP200 Claw domain with *bs*CspB and LC3B. Cartoon representation, topology plot and electrostatic surface potential of E) FIP200 Claw domain, F) bsCspB bound to dT_7. (PDB code: 2ES2) and G) LC3B bound to the $p62^{334\text{-}344}$ LIR motif (PDB code: 2ZJD). Electrostatic surface potential in ±5 k_bT/e_c. Ligands are depicted in yellow stick representation. The arrow in S4E indicates the putative p62 FIR binding pocket.

Figure S5 - related to Fig. 4 and 5

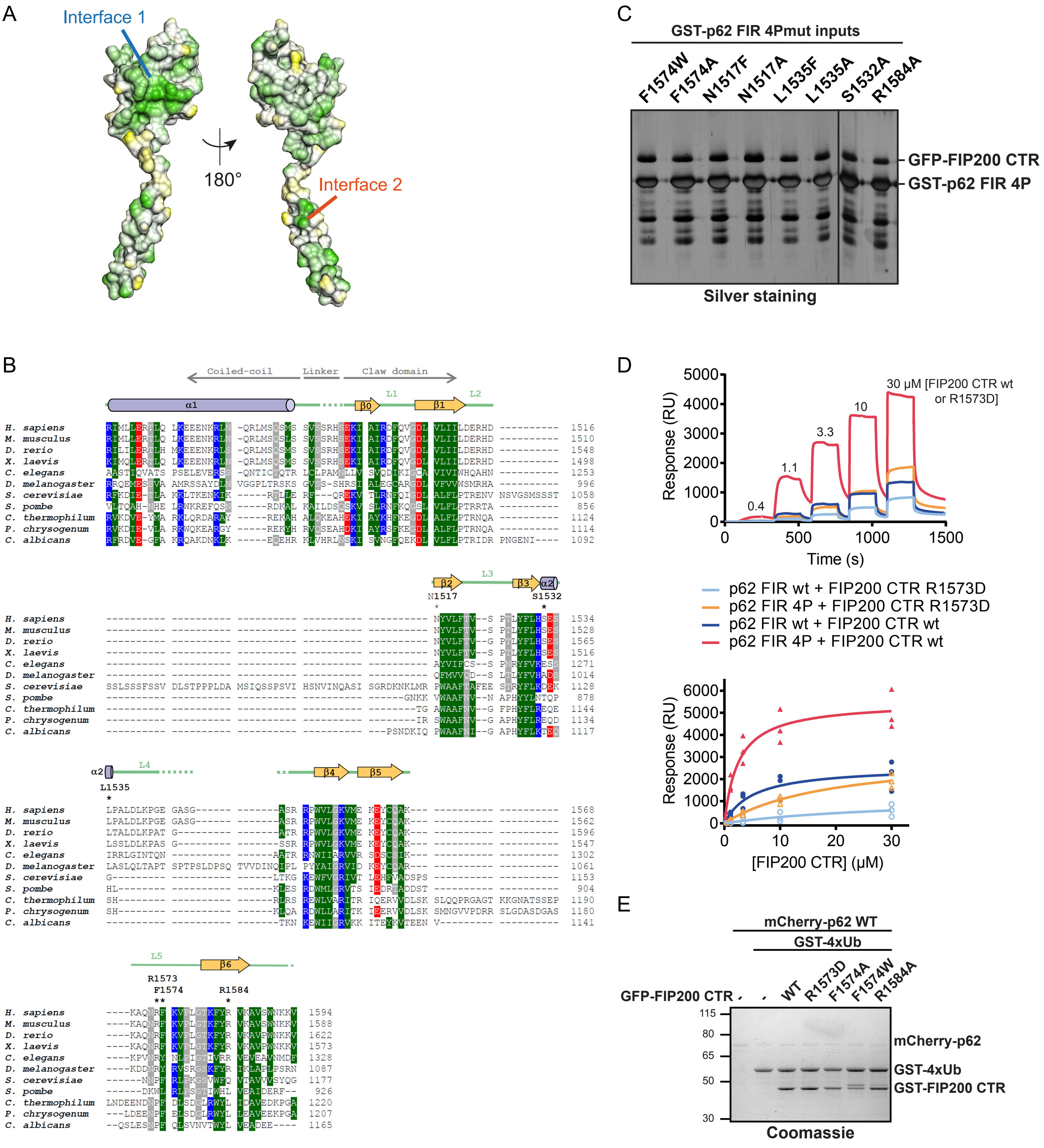

Figure S5, related to Figure 4 and 5.

A) Lipophilic surface potential of the FIP200 CTR monomer. Lipophilic and hydrophilic residues are colored in green and yellow, respectively.

B) Sequence alignment of the following Atg11 homology regions from different species (UniProt accession code in parentheses). *H. sapiens* (Q8TDY2), *M. musculus*, (Q9ESK9), *D. rerio*, (E7FFM2), *X. laevis* (A0A1L8FYZ6), *C. elegans* (Q22342), *D. melanogaster* (Q7KTS2), *S. cerevisiae* (Q12527), *S. pombe* (O14261), *C. thermophilum*, (G0S0K0), *P. chrysogenum* (A0A167XQU9), *C. albicans* (Q5AMN3). Residues with a conservation greater than 60% are color-coded (D, E in red; R, K, H in blue N, Q, S, T, G, P in gray; A, L, I, V, F, Y, W, M, C in green). FIP200 CTR secondary structure is displayed on top of the alignment. α-Helices are shown as cylinders and β-strands as arrows. Mutated residues in this study are indicated with an asterisk (*).

C) The samples (beads + supernatant) of the experiment in Fig. 5B were analyzed by SDS-PAGE followed by Silver staining.

D) Representative SPR sensorgrams (top) corrected for background binding to GST and buffer control. The experiment was performed as described in Fig. 2E to analyze the binding of GFP-FIP200 CTR wt and R1573D to GST-p62 FIR wt and 4P. Equilibrium analysis (bottom) for the FIP200 CTR variants: GST-p62 FIR variants interaction was performed as in Fig. 2F. Binding of the FIP200 CTR R1573D was too weak to be accurately fitted for a $K_D$app determination. Data were collected as two technical replicates of three independent experiments.

E) Loading control for the experiment in Fig. 5D. After imaging, samples were recovered and analyzed by SDS-PAGE followed by Coomassie staining.

Figure S6 - related to Fig. 6

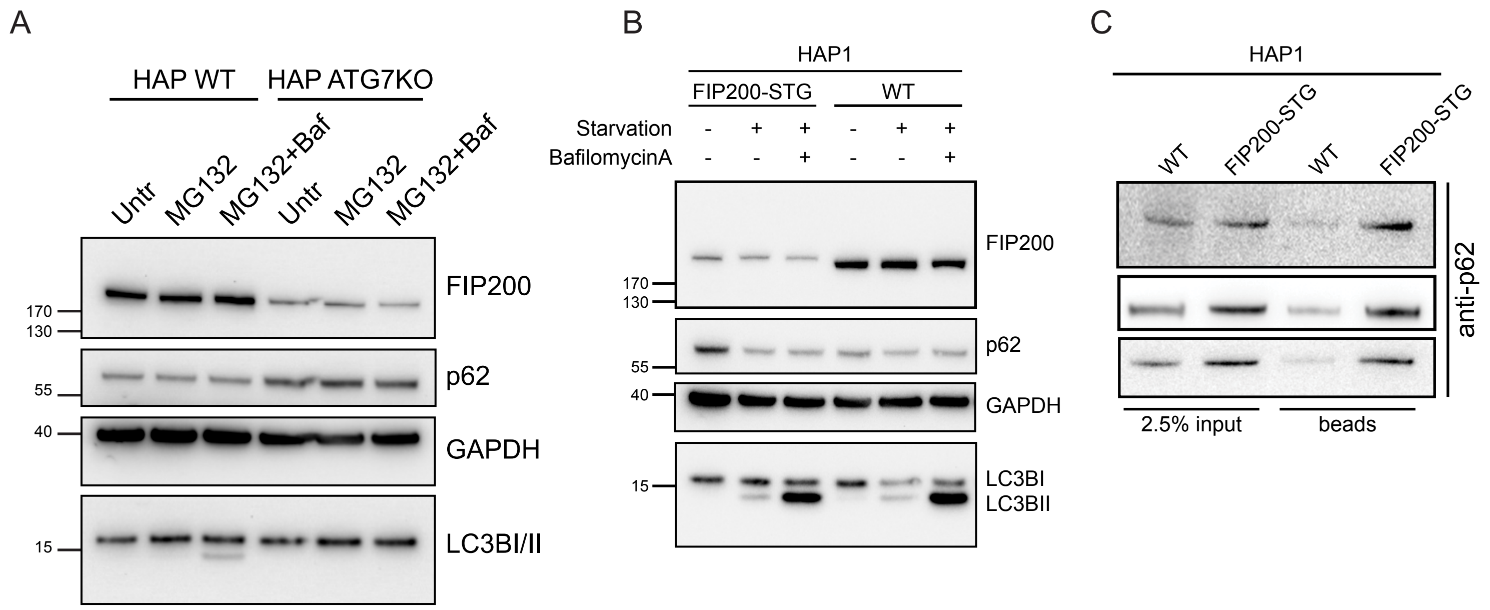

Figure S6, related to Figure 6

A) HAP1 wt or ATG7ko were left untreated or treated with MG132 (+/- Bafilomycin). After 1 h treatment, cell lysates were analyzed by Western blot to assess the ability to perform LC3B lipidation. FIP200 and p62 levels in the cells were also analyzed while GAPDH signal was used as loading control.

B) Validation of the HAP1 FIP200-STG cell line. Bulk autophagy was induced by starvation (in presence or absence of Bafilomycin) in HAP1 wt and FIP200-STG cell lines. Cell lysates were analyzed by Western blotting with anti-FIP200, anti-p62 and anti-LC3B. Anti-GAPDH was used as loading control. Overexpression of FIP200 caused cell death in the cell lines used.

C) 3 additional replicates of the affinity co-purification in Fig. 6C are shown.

Figure S7 - related to Fig. 7

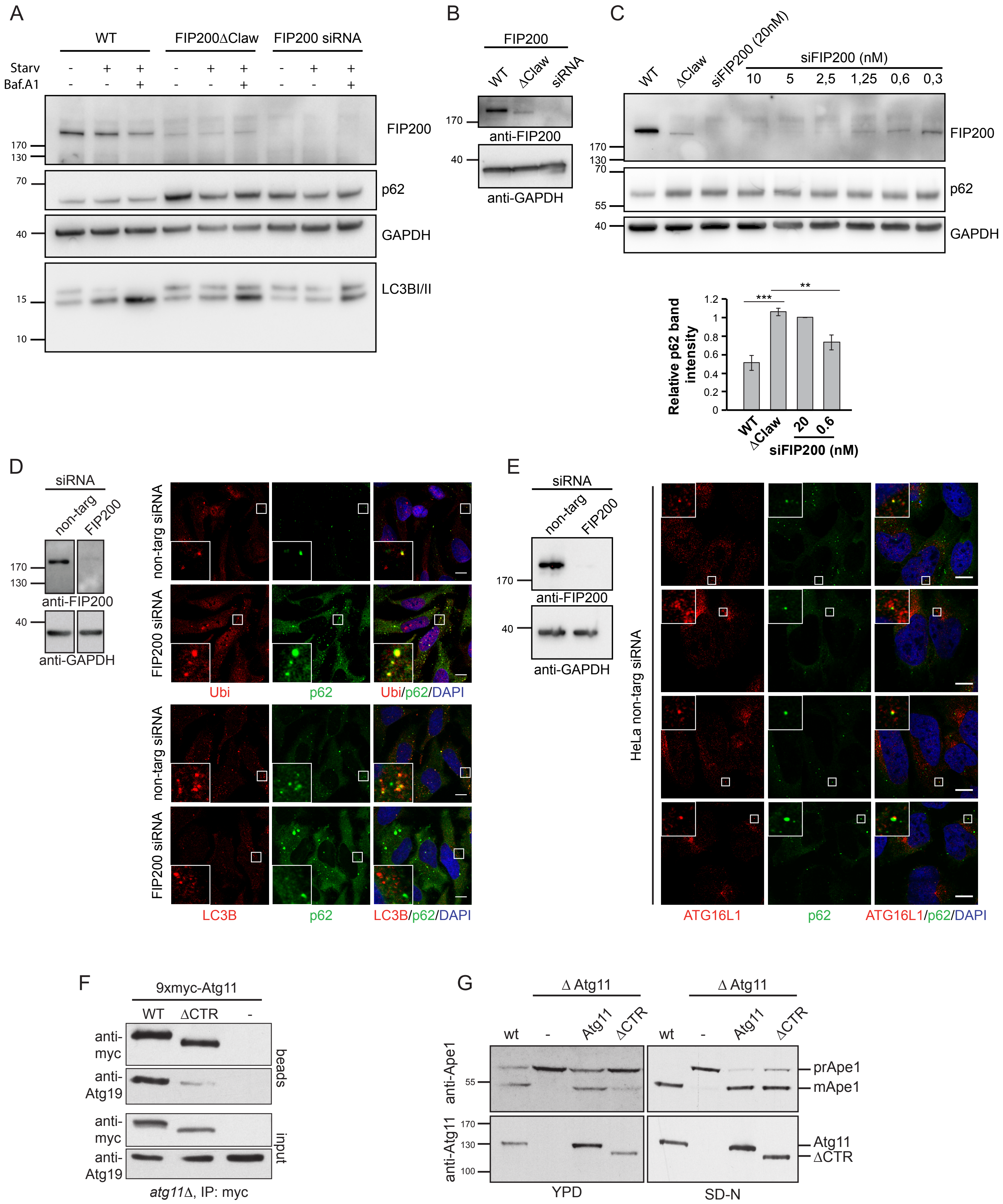

Figure S7, related to Figure 7.

A) HeLa wt and FIP200ΔClaw, treated with non-targeting siRNA and HeLa wt treated with FIP200 siRNA were left untreated or starved (in presence or absence of Bafilomycin). Cell lysates were analyzed by Western blotting for autophagy markers (p62 accumulation and LC3B lipidation) and FIP200. GAPDH levels were used as loading control.

B) Expression levels of FIP200 wt and FIP200ΔClaw in HeLa cells were analyzed by Western blotting using lysates collected from low-passage number (3) cells.

C) Higher passage number HeLa FIP200ΔClaw were treated with non-targeting siRNA and wt cells were treated either with non-targeting siRNA or the indicated concentrations of FIP200 siRNA. Then, FIP200 expression levels and p62 levels were analyzed by Western blotting. p62 band intensity for the samples shown in the plot was measured with ImageJ and normalized to the amount of GAPDH in the lysate. Average p62 band intensity (relative to p62 levels in HeLa treated with 20 nM FIP200 siRNA) and standard error for 4 independent experiments are shown. Significant differences are indicated with * when p value % 0.05, ** when p value % 0.01, and *** when p value % 0.001.

D) Immunofluorescence staining with ubiquitin and p62 or p62 and LC3B relative to the quantification showed in Fig. 7D. HeLa cells treated with non-targeting siRNA or FIP200 siRNA were fixed and immuno-stained as indicated. Scale bar = 10 µm. The efficiency of the siRNA treatment was assessed by Western blotting.

E) Cells used for the immunofluorescence staining in Fig. 7E were analyzed by Western blotting with anti-FIP200 to assess the efficiency of the siRNA treatment. Additional images of p62-ATG16L1 immunofluorescence in HeLa cells treated with non-targeting siRNA are shown. Scale bar = 10 µm.

E) Co-immunoprecipitation of *S. cerevisiae* Atg11 and Atg19. An Atg11-deficient yeast strain was transformed with plasmids expressing myc-Atg11 (full-length or ΔCTR, under endogenous promoter) or empty plasmid. Atg11 was immunoprecipitated using the myc tag and samples were probed with Atg19 antibody.

F) prApe1 processing assay using Atg11 deficient yeast strains transformed with the indicated Atg11 constructs. Cells were grown in YPD (rich) or SD-N (starvation) medium and cell lysates were analyzed by Western blotting. Conversion of prApe1 into mApe1 was used to monitor the selective Cvt pathway.

Table S1-related to Figure 6C: Mass Spectrometry analysis of FIP200-STG purified from Hap1 cells.

Gene Name	Protein Name	Score	Sequence coverage (%)	Log2(LFQ) (STG-FIP200-ST-GFP/wt)
**RB1CC1**	FIP200/RB1-inducible coiled-coil protein 1	323,3	68,5	13,07
**ULK1**	Serine/threonine-protein kinase ULK1	323,3	51,7	12,41
**ATG13**	Autophagy-related protein 13	323,3	54,4	9,9
**ATG101**	Autophagy-related protein 101	267,8	73,9	11,96
**SQSTM1**	p62/Sequestosome-1	227,1	38,2	1,68

Table S1. FIP200-STG and wt Hap1 cells were subjected to StrepTactin purification followed by mass spectrometry. Listed are the known FIP200 direct interactors enriched in the FIP200-STG sample compared to the wt. A Log2(LFQ)≥1 was considered as significant enrichment. LFQ: label-free quantitation.
