# Supplementary material for: FIP200 Claw Domain Binding to p62 Promotes Autophagosome Formation at Ubiquitin Condensates
Source: Mol Cell. 2019 Apr 18;74(2):330–346.e11. doi: 10.1016/j.molcel.2019.01.035 (PMC6477179; doi:10.1016/j.molcel.2019.01.035)
Supplement: Document S2. Article plus Supplemental Information [file mmc2.pdf]

Molecular Cell

Article

FIP200 Claw Domain Binding to p62 Promotes Autophagosome Formation at Ubiquitin Condensates

Graphical Abstract

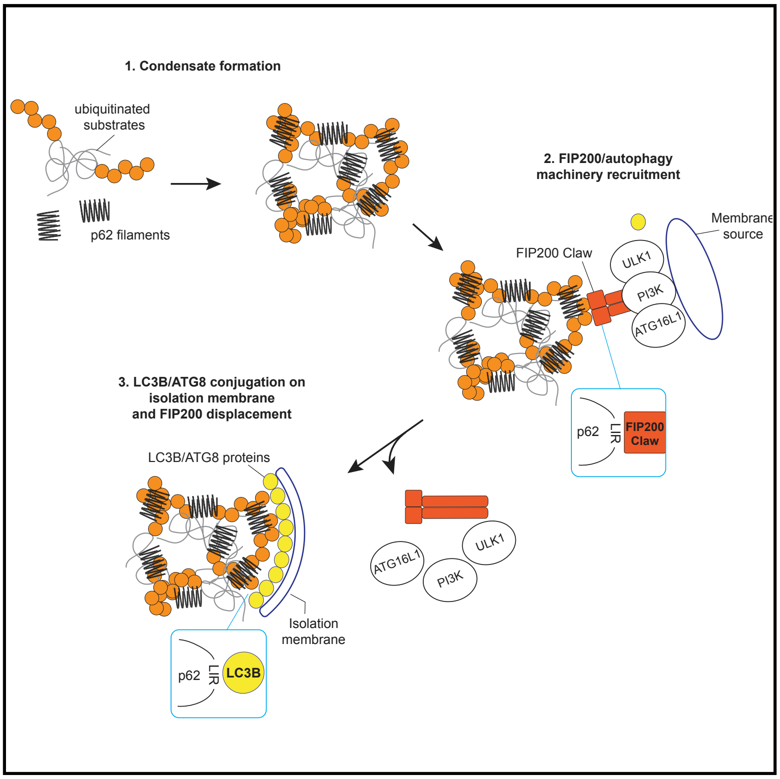

Authors

Eleonora Turco, Marie Witt, Christine Abert, ..., James H. Hurley, Oliver Daumke, Sascha Martens

Correspondence

oliver.daumke@mdc-berlin.de (O.D.), sascha.martens@univie.ac.at (S.M.)

In Brief

Turco et al. show that p62 interacts with the claw-shaped C-terminal domain of FIP200. This interaction is enhanced by p62 phosphorylation, mutually exclusive with the binding of p62 to LC3B, and promotes degradation of ubiquitinated cargo by autophagy. The recruitment of the FIP200 Claw slows the formation of p62-ubiquitin condensates in a reconstituted system.

Highlights

- p62 directly interacts with the FIP200 C-terminal domain
- Structural studies reveal a claw shape of the conserved FIP200 C-terminal domain
- p62-ubiquitin condensates recruit FIP200 via the Claw to promote their degradation
- LC3B outcompetes FIP200 from p62, suggesting an inbuild directionality in the system

Turco et al., 2019, Molecular Cell *74*, 330–346
April 18, 2019 © 2019 The Author(s). Published by Elsevier Inc.
https://doi.org/10.1016/j.molcel.2019.01.035

CellPress

Molecular Cell
Article

FIP200 Claw Domain Binding to p62 Promotes Autophagosome Formation at Ubiquitin Condensates

Eleonora Turco,[1,6] Marie Witt,[2,3,6] Christine Abert,[1,6] Tobias Bock-Bierbaum,[2,6] Ming-Yuan Su,[4,5,6] Riccardo Trapannone,[1] Martin Sztacho,[1,8] Alberto Danieli,[1] Xiaoshan Shi,[4] Gabriele Zaffagnini,[1,9] Annamaria Gamper,[1,10] Martina Schuschnig,[1] Dorotea Fracchiolla,[1] Daniel Bernklau,[1] Julia Romanov,[1] Markus Hartl,[1] James H. Hurley,[4,5,7] Oliver Daumke,[2,3,7,*] and Sascha Martens[1,7,11,*]

[1]Department of Biochemistry and Cell Biology, Max F. Perutz Laboratories (MFPL), University of Vienna, Vienna BioCenter, Dr. Bohr-Gasse 9, 1030 Vienna, Austria
[2]Department of Crystallography, Max-Delbrück-Center for Molecular Medicine, Robert-Rössle-Strasse 10, 13125 Berlin, Germany
[3]Institute of Chemistry and Biochemistry, Freie Universität Berlin, Takustrasse 6, 14195 Berlin, Germany
[4]Department of Molecular and Cell Biology and California Institute for Quantitative Biosciences, University of California, Berkeley, Berkeley, CA 94720, USA
[5]Molecular Biophysics and Integrated Bioimaging Division, Lawrence Berkeley National Laboratory, Berkeley, CA 94720, USA
[6]These authors contributed equally
[7]Senior author
[8]Present address: Institute of Molecular Genetics, Vídeňská 1083, Prague 4, Czech Republic
[9]Present address: Centre for Genomic Regulation (CRG), C/ Dr. Aiguader, 88, 08003 Barcelona, Spain
[10]Present address: Institute of Biochemistry, ETH Zürich, Otto-Stern-Weg 3, 8093 Zürich, Switzerland
[11]Lead Contact
*Correspondence: oliver.daumke@mdc-berlin.de (O.D.), sascha.martens@univie.ac.at (S.M.)
https://doi.org/10.1016/j.molcel.2019.01.035

SUMMARY

The autophagy cargo receptor p62 facilitates the condensation of misfolded, ubiquitin-positive proteins and their degradation by autophagy, but the molecular mechanism of p62 signaling to the core autophagy machinery is unclear. Here, we show that disordered residues 326–380 of p62 directly interact with the C-terminal region (CTR) of FIP200. Crystal structure determination shows that the FIP200 CTR contains a dimeric globular domain that we designated the "Claw" for its shape. The interaction of p62 with FIP200 is mediated by a positively charged pocket in the Claw, enhanced by p62 phosphorylation, mutually exclusive with the binding of p62 to LC3B, and it promotes degradation of ubiquitinated cargo by autophagy. Furthermore, the recruitment of the FIP200 CTR slows the phase separation of ubiquitinated proteins by p62 in a reconstituted system. Our data provide the molecular basis for a crosstalk between cargo condensation and autophagosome formation.

INTRODUCTION

The removal of aggregated proteins from the cytoplasm is essential for cellular homeostasis. Two ubiquitin-based systems, the ubiquitin-proteasome system (UPS) and macroautophagy (hereafter autophagy), are major pathways for the degradation of these substances. During autophagy, substrates referred to as cargo are sequestered within autophagosomes (Zaffagnini and Martens, 2016). The formation of autophagosomes requires the hierarchical action of a number of conserved factors, including the upstream-acting ULK1 complex containing the scaffold protein FIP200 and the downstream-acting conjugation machinery, which catalyzes the conjugation of the ubiquitin-like ATG8 proteins to the head group of phosphatidylethanolamine on the isolation membrane (Hurley and Young, 2017).

The selectivity of autophagic processes is mediated by cargo receptors, which link the cargo material to the nascent autophagosomal membrane (Stolz et al., 2014). Cargo receptors also play crucial roles in the formation of autophagosomes in vicinity of the cargo, by recruiting upstream components of the autophagy machinery (Fracchiolla et al., 2016; Zaffagnini and Martens, 2016). For example, in *S. cerevisiae*, the Atg19 cargo receptor recruits the scaffold protein and Atg1/ULK1 kinase complex member Atg11 to the prApe1 cargo in the cytoplasm-to-vacuole targeting (Cvt) pathway (Kamber et al., 2015; Shintani et al., 2002; Torggler et al., 2016). In mammals, optineurin and NDP52 recruit the autophagy machinery to damaged mitochondria (Lazarou et al., 2015). In addition, ER-resident transmembrane proteins in yeast and mammalian cells have been shown to recruit the autophagy machinery via the Atg11/FIP200 scaffold proteins (Khaminets et al., 2015; Mochida et al., 2015; Smith et al., 2018). The molecular mechanisms that link cargo receptors to the core autophagy machinery are mostly obscure.

p62/SQSTM1 is a major cargo receptor for the selective degradation of misfolded, ubiquitinated proteins by autophagy in a process that is referred to as aggrephagy (Zaffagnini and

 © 2019 The Author(s). Published by Elsevier Inc.
This is an open access article under the CC BY license (http://creativecommons.org/licenses/by/4.0/).

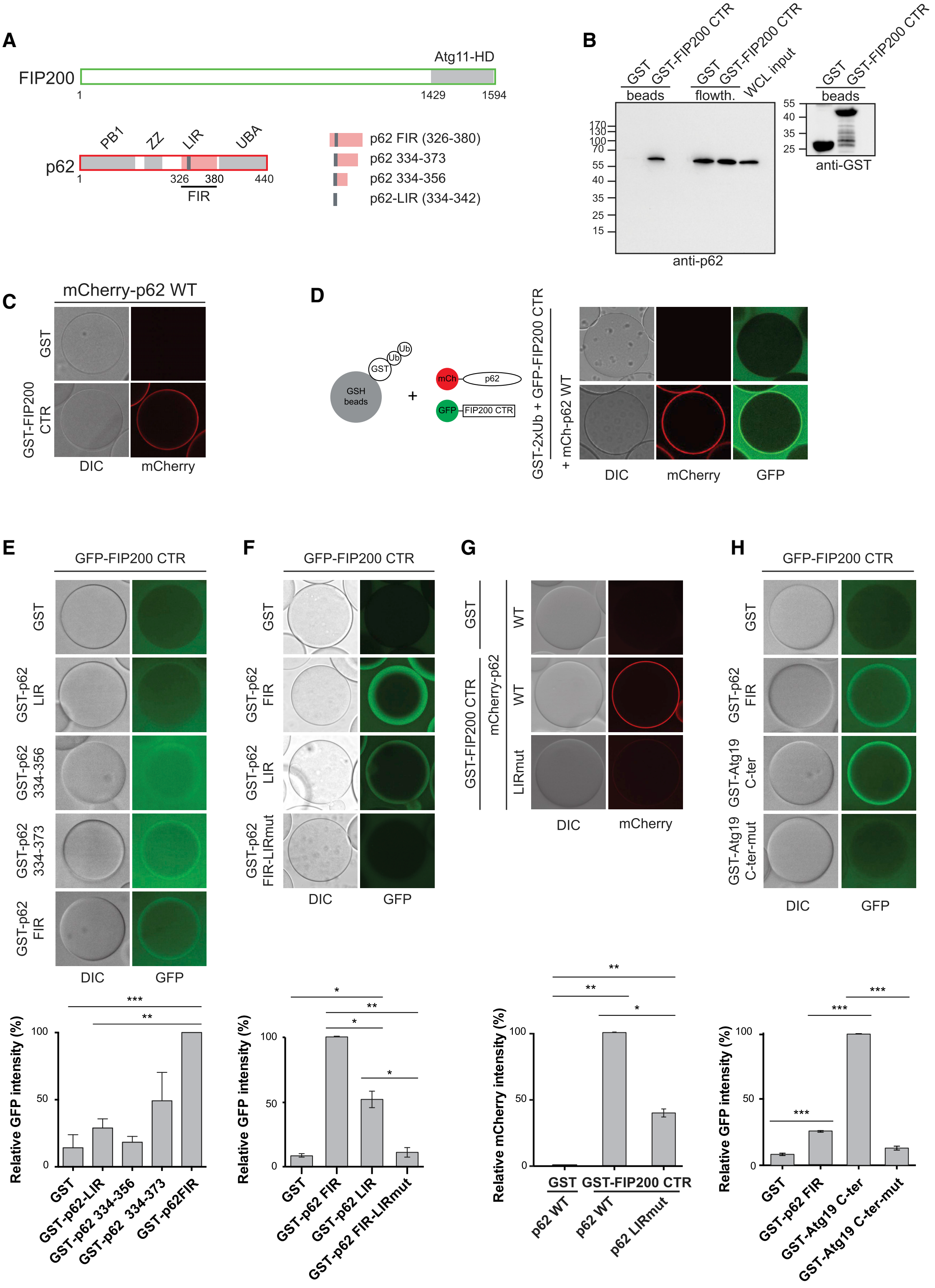

Figure 1. The FIP200 C-Terminal Region Directly Interacts with p62 in a LIR-Dependent Manner

(A) Schematic representation of FIP200 and p62. The Atg11 homology domain of FIP200 is depicted in gray. The FIP200-interacting region (FIR) of p62 is depicted in pink and the LIR motif in dark gray. p62 constructs covering parts of the FIR are shown on the right.

(legend continued on next page)

Martens, 2016). Mutations in p62 are associated with neurodegenerative diseases including amyotrophic lateral sclerosis and frontotemporal dementia (Sánchez-Martín and Komatsu, 2018). p62 oligomerizes via its N-terminal PB1 domain (Figure 1A) and exists as oligomers in cells (Ciuffa et al., 2015; Lamark et al., 2003; Zaffagnini et al., 2018). Its interaction with ubiquitinated proteins is mediated by a C-terminal UBA domain (Seibenhener et al., 2004), while the binding to ATG8 proteins is conferred by a LIR motif, which is located in a disordered region (Ichimura et al., 2008; Pankiv et al., 2007).

p62 functions at multiple steps of aggrephagy. It mediates the phase separation of misfolded, ubiquitinated proteins into larger condensates and it also functions to tether them to nascent autophagosomal membranes via its LIR motif-dependent interaction with ATG8 family proteins, such as LC3B and GABARAP (Bjørkøy et al., 2005; Ichimura et al., 2008; Pankiv et al., 2007; Sun et al., 2018; Wurzer et al., 2015; Zaffagnini et al., 2018). However, it is unclear how the p62-mediated generation of ubiquitin-positive condensates is linked to the formation of autophagosomes.

We show that p62 recruits the ULK1 kinase complex subunit FIP200 to ubiquitin-positive condensates. This recruitment is mediated by the interaction of the FIP200 C-terminal region (CTR) with the region of p62 surrounding the LIR motif. The crystal structure of the FIP200 CTR reveals a positively charged pocket required for the binding of the p62. The FIP200 CTR inhibits clustering of ubiquitinated substrates in a fully reconstituted system and that the FIP200 CTR can be outcompeted from p62 by LC3B. Our data suggest a cascade of binding events that coordinate phase separation, initiation of isolation membrane formation, and its elongation played out at the LIR motif region of p62.

RESULTS

The FIP200 CTR Directly Interacts with p62 in a LIR-Dependent Manner

p62 contains a short region of weak homology with the Atg11-binding motif of yeast Atg19 (aa358–370 of p62) (Sawa-Makarska et al., 2014) (Figure S1A). At the same time, the CTR of FIP200 contains an Atg11 homology domain which shows sequence similarity to the corresponding region of yeast Atg11 (Figure 1A). We therefore hypothesized that, similar to the situation in the yeast-specific Cvt pathway, p62 may recruit the upstream autophagy machinery via FIP200 during aggrephagy. To test this, we fused the FIP200 CTR to glutathione S-transferase (GST) and used this protein as a bait for pull-downs with HeLa cell lysates. Indeed, p62 was specifically pulled down by the FIP200 CTR (Figure 1B). To test for a direct interaction, we immobilized GST-FIP200 CTR on beads and determined the recruitment of recombinant mCherry-p62 by microscopy (Figure 1C). p62 was robustly recruited to the beads, pointing to a direct interaction. p62 also recruited the FIP200 CTR to ubiquitinated proteins (Figure 1D).

Next, we determined which region of p62 interacts with the FIP200 CTR. We found that the region encompassing amino acids (aa) 326–380 is sufficient for the interaction (Figures 1A, 1E, and S1B). We refer to this sequence as the "FIP200-interacting region (FIR)." Further truncation resulted in weakening of the interaction (Figures 1A and 1E).

Interestingly, the FIR contains the LIR motif of p62, which is responsible for the interaction with ATG8 proteins and required for the efficient phase separation of ubiquitinated substrates (Ichimura et al., 2008; Pankiv et al., 2007; Zaffagnini et al., 2018). Therefore, we tested whether the LIR motif would be sufficient for the interaction with the FIP200 CTR. Indeed, the LIR motif interacted with the FIP200 CTR, albeit weaker than the entire FIR. When we mutated the LIR motif in the context of the FIR, its FIP200 CTR binding activity was completely lost (Figures 1F and S1C). Similarly, mutation of the LIR motif in full-length p62 reduced its binding to the FIP200 CTR (Figures 1G and S1D).

A part of the p62 FIR shows weak sequence similarity to the Atg19 C terminus, which contains the Atg11 binding motif and multiple LIR/AIM motifs (Sawa-Makarska et al., 2014) (Figures S1A and S1F). We tested whether the FIP200 CTR would also interact with the Atg19 C terminus. Indeed, the FIP200 CTR was robustly recruited to the Atg19 C terminus. Furthermore,

(B) GST or GST-FIP200 CTR were coupled to glutathione (GSH) beads and incubated with HeLa cells lysates (200 μg). Beads were washed, and the beads/flow-through fractions were analyzed by western blot using anti-p62 antibody. The bound sample was probed with anti-GST to visualize the amount of bait protein on the beads.

(C) GSH beads were coated with GST or GST-FIP200 CTR and incubated with recombinant mCherry-p62 (2 μM). Beads were imaged by microscopy.

(D) The experimental setup is shown on the left. GSH beads were coated with GST-2x ubiquitin. Excess GST-2x ubiquitin was washed off, and beads were incubated with mCherry-p62 (2 μM) and GFP-FIP200 CTR aa 1429–2594 (5 μM). After 1 h incubation, beads were imaged by microscopy.

(E) GSH beads were coated with the indicated GST-p62 constructs and incubated with GFP-FIP200 CTR aa 1429–1594 (5 μM). Beads at equilibrium were imaged by microscopy. Protein inputs are shown in Figure S1B. The GFP signal on the beads was normalized to the signal of GFP-FIP200 CTR bound to GST-p62 FIR-coated beads. Average intensity and SEM for n = 3 are plotted. Significant differences are indicated with * when p value $\leq$ 0.05, ** when p value $\leq$ 0.01, and *** when p value $\leq$ 0.001.

(F) Same experimental setup and quantification used in (E). Beads were imaged by microscopy. Protein inputs are shown in Figure S1C. The GST-p62 FIR-LIRmut construct carries a mutation in the LIR motif ($_{335}$DDDW$_{338}$ > AAAA).

(G) Same experimental setup used in (C). In the mCherry p62 LIRmut residues 335-338 are mutated to Ala. Protein inputs are shown in Figure S1D. The mCherry signal on the beads was normalized to the signal of mCherry-p62 WT on GST-FIP200 CTR-coated beads. The average intensity and SEM for n = 3 are shown. Significant differences are indicated with * when p value $\leq$ 0.05, ** when p value $\leq$ 0.01, and *** when p value $\leq$ 0.001.

(H) The same experimental setup used in (E) and (F) was used to test the interaction of the Atg19 C terminus (Atg19 C-ter) with the FIP200 CTR. In the Atg19 C-ter-mut, the three LIR motifs (Figure S1F) are mutated. The graph on the bottom shows the average GFP intensity, normalized to the signal of GFP-FIP200 CTR binding to GST-Atg19 C-ter, and SEM for n = 3. Significant differences are indicated with * when p value $\leq$ 0.05, ** when p value $\leq$ 0.01, and *** when p value $\leq$ 0.001. Protein inputs are shown in Figure S1E.

See also Figure S1.

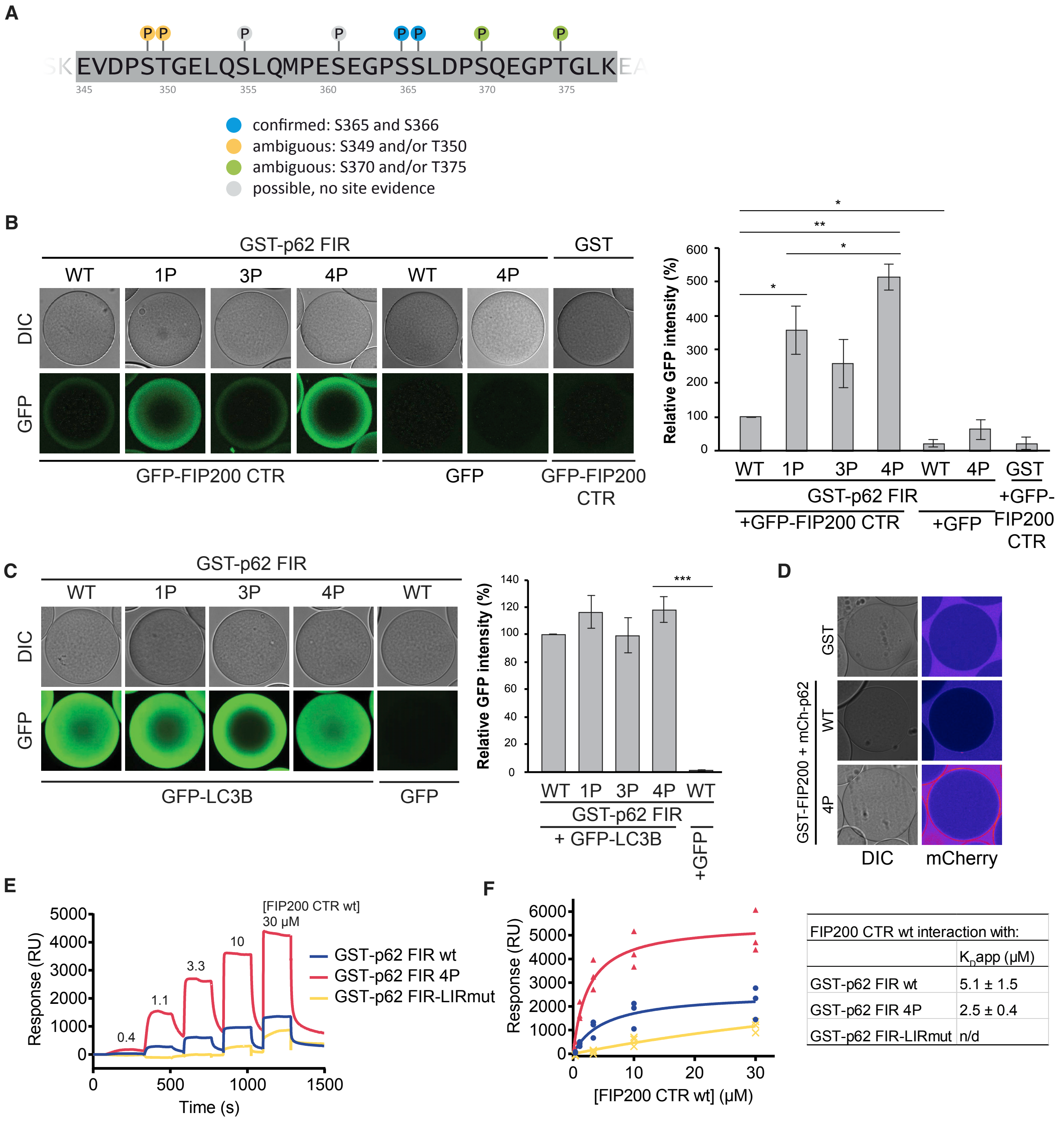

Figure 2. p62 FIR Phosphorylation Enhances FIP200 Binding

(A) Overview of phosphosites in the FIR of affinity-purified p62 as identified by mass spectrometry (Figures S2A and S2B).

(B) Phospho-mimicking mutations were introduced in GST-p62 FIR as follows: 1P (S349D); 3P (S365D, S366D, S370D); 4P (S349D, S365D, S366D, S370D). GSH beads were coated with GST or the GST-p62 FIRs, incubated with GFP-FIP200 CTR (aa 1458–1594) and imaged by microscopy at equilibrium. GFP signals on the beads were normalized to the signal of GFP-FIP200 CTR bound to GST-p62 FIR 4P. Average intensity and SEM for n = 3 are shown. Significant differences are indicated with * when p value $\leq$ 0.05, ** when p value $\leq$ 0.01, and *** when p value $\leq$ 0.001. The same beads were analyzed by SDS-PAGE like in a pull-down experiment (Figure S2D).

(C) The binding of GFP-LC3B to GST-p62 phospho-mimicking mutants was assessed like in (B).

(D) GSH beads were coated with full-length GST-FIP200 and incubated with full-length mCherry-p62 WT or the 4P phospho-mimicking mutant. Beads were imaged by microscopy. The mCherry signal is shown in false color (ImageJ: Fire).

(legend continued on next page)

mutation of the LIR motifs in the Atg19 C terminus abolished the interaction (Figures 1H and S1E) suggesting that the LIR-dependent interaction of the Atg11 homology region with cargo receptors is conserved from yeast to human. Consistently, the Atg19 C terminus directly interacted with the Atg11 CTR, and this interaction was lost upon mutation of the LIR motifs in the Atg19 C terminus (Figure S1F).

Phosphorylation of the p62 FIR Enhances FIP200 Binding

In *yeast*, the interaction of the Atg19 and Atg34 cargo receptors with Atg11 is positively regulated by phosphorylation in their Atg11 binding regions (Figure S1F) (Mochida et al., 2014; Pfaffenwimmer et al., 2014; Tanaka et al., 2014). Therefore, we asked whether the interaction of the p62 FIR with the FIP200 CTR also could be enhanced by phosphorylation. We first performed mass spectrometry to detect phosphorylated S/T residues within the FIR of p62 isolated from cells. We detected phosphorylation of S349/T350, S365, 366, and S370/T375 (Figures 2A, S2A, and S2B). S349 was previously shown to be phosphorylated (Sánchez-Martín and Komatsu, 2018). S365 and S366 are located in the region showing homology to the Atg11 binding site of Atg19 and Atg34. For phospho-S349, a specific antibody is available, and we corroborated the mass spectrometry data for this residue using HeLa and HAP1 cell lysates (Figure S2C). We readily detected p62 phosphorylated on S349 under all conditions tested and the amount of phospho-S349 p62 detected generally correlated with protein abundance. Treatment with the lysosomal inhibitor bafilomycin increased the signal of phospho-S349 p62 suggesting that the phosphorylated protein is transported into the lysosome (Figure S2C).

We went on to test whether the phosphorylation of p62 would enhance its interaction with the FIP200 CTR. To this end, we introduced a single phospho-mimetic mutation S349D (referred to as "1P") in the FIR of p62. We also separately mutated S365, S366 to D, and additionally included the S370D mutation, since the corresponding residue is phosphorylated in Atg19 (Pfaffenwimmer et al., 2014), and we have found evidence for phosphorylation of S370/T375 by mass spectrometry. We refer to this triple mutant as 3P and to the mutant containing all four phospho-mimetic mutations as 4P. When we tested the recruitment of the FIP200 CTR to the different p62 phospho-mimetic FIR mutants, it became evident that all of them enhanced the interaction with the FIP200 CTR. S349D had a stronger effect than the triple mutant and the quadruple mutant showed the strongest interaction (Figures 2B and S2D). In contrast, the phospho-mimicking mutations of p62 FIR did not affect its binding to LC3B (Figure 2C). The p62 4P FIR mutant also showed an increased interaction with FITC-labeled full-length FIP200 when compared to the wild-type (WT) FIR (Figure S3A). Furthermore, phospho-mimicking mutation of full-length p62 (mCherry-p62 4P) showed a stronger interaction with full-length FIP200 than WT p62 (Figure 2D).

To obtain a more quantitative analysis of the interaction between the p62 FIR and the FIP200 CTR, we performed surface plasmon resonance (SPR) experiments. The sensorgrams showed steep binding and dissociation curves indicating high on- and off-rates (Figure 2E). Equilibrium analysis for the calculation of an apparent K_D showed binding affinities in the low micromolar range and a 2-fold increase in affinity for p62 FIR 4P compared to p62 FIR WT (Figure 2F). In contrast, the binding signal of FIP200 to p62 FIR-LIRmut was too weak to be accurately fitted (Figures 2E and 2F).

FIP200 and LC3B Binding to p62 Is Mutually Exclusive

Since the interaction of the p62 FIR and the FIP200 CTR was LIR dependent, we asked whether it is mutually exclusive with the interaction of p62 with LC3B. To test this, we immobilized mCherry-p62 4P on RFP-trap beads and added the GFP-FIP200 CTR. This was followed by the addition of increasing amounts of LC3B. Indeed, LC3B outcompeted the GFP-FIP200 CTR from the beads in a concentration-dependent manner (Figures 3A and S3B). We found the same effect for the Atg19 C terminus-Atg11 CTR interaction, which was mutually exclusive with the interaction of the Atg19 C terminus with Atg8 (Figure S3C). These results suggest that LC3B and other ATG8 proteins located and concentrated on the isolation membrane may displace FIP200 from p62 as the cargo receptors engage in the interaction with ATG8 proteins. This, in turn, would suggest that FIP200 is largely excluded from autophagosomal lumen and inefficiently delivered into the lysosome for degradation. To test this hypothesis, we performed protease protection assays with autophagosome-containing fractions isolated from cells. The luminal p62 and LC3B were partly protease-resistant after bafilomycin treatment, FIP200 and the outer autophagosomal protein STX17 were protease sensitive, suggesting that they are not included in the autophagosomal lumen in significant amounts (Figure 3B). The observed protease-protection for p62 and LC3B is conferred by the autophagosomal membrane since protection is lost when cells are treated with the autophagy inhibitor wortmannin (Figure S3D).

Structure of the FIP200 CTR

To gain mechanistic insights into the FIP200-p62 interaction, we determined the X-ray structure of the FIP200 CTR (aa 1458–1594) (Figure 4). Crystals of this construct diffracted to a resolution of 3.2 Å and the phase problem was solved by a single anomalous dispersion approach using selenomethione-substituted crystals (Table 1; Figure S4A). In the asymmetric

(E) Representative SPR sensorgrams corrected for background binding to GST and buffer control. FIP200 CTR was passed over four flow channels with immobilized GST or GST-p62 FIR variants. Association of FIP200 CTR (0.4, 1.1, 3.3, 10, and 30 μM) was monitored for 180 s, followed by dissociation in buffer for 70 s.

(F) Equilibrium analysis to determine the apparent dissociation constants, $K_D$app. Response signals at equilibrium were plotted against the concentration of FIP200 CTR and globally fitted with a one site binding model. Three independent experiments with two technical replicates each were recorded for a 3-fold dilution series of FIP200 CTR (0.4–30 μM). Derived $K_D$app are shown in the table (right).

See also Figures S2 and S3.

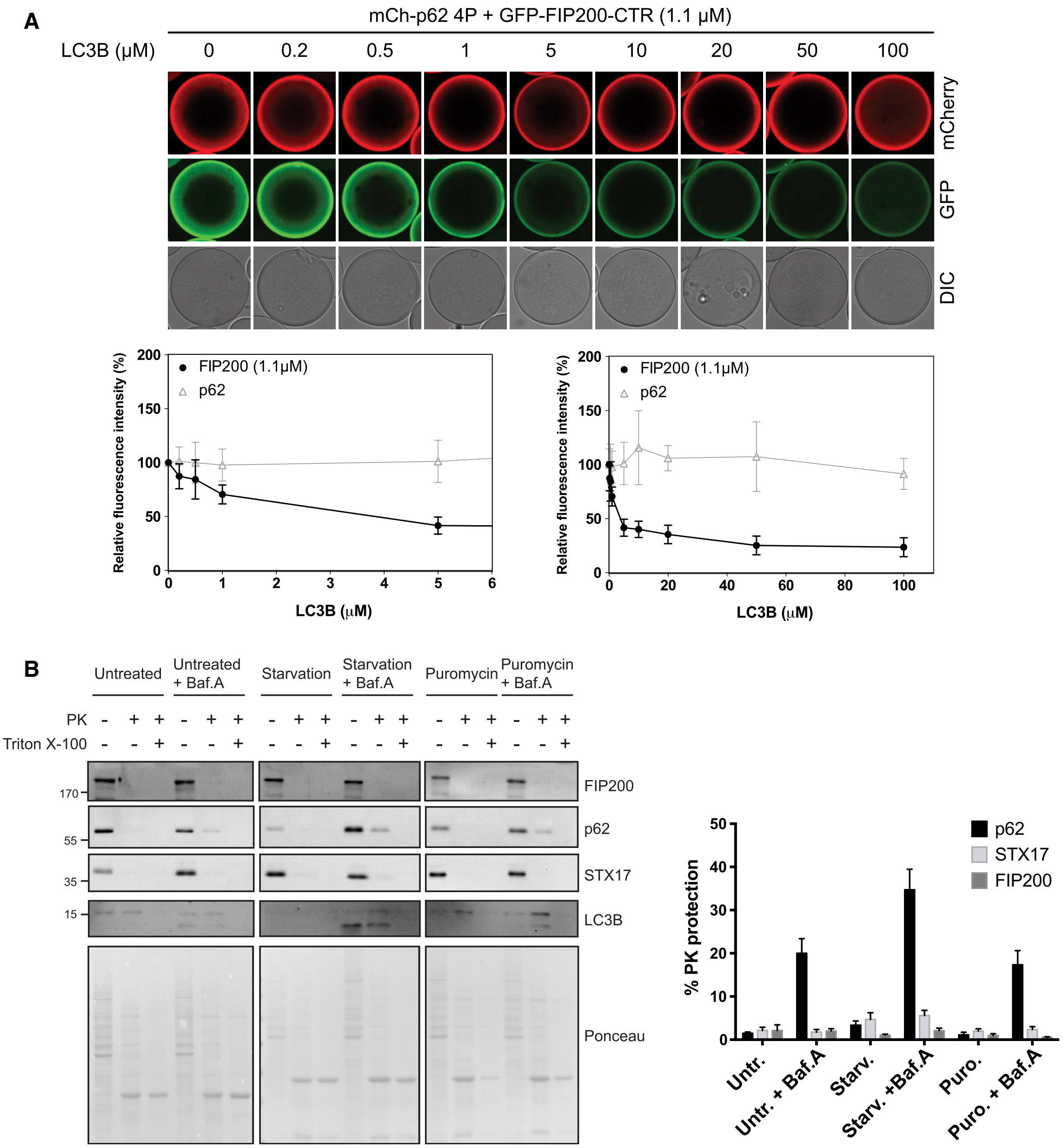

Figure 3. LC3B Competes with FIP200 for p62 Binding, and FIP200 Is Excluded from the Autophagosomal Lumen

(A) RFP trap beads were coated with mCherry-p62 FIR 4P and incubated with the GFP-FIP200 CTR (aa 1458-1594, 1.1 µM). Then, increasing concentrations of LC3B were added. Beads were imaged by microscopy. The intensities of the GFP and mCherry signals on the beads were measured and plotted against the LC3B concentrations (lower panels). A zoom-in of the plot at the lowest LC3B concentrations is shown on the left. Average intensities and SD for n = 3 are shown. Negative and positive controls of binding are shown in Figure S3B.

(B) HeLa cells were left untreated, starved or treated with puromycin both in presence or absence of bafilomycin. Cell lysates were then treated with proteinase K in the presence or absence of Triton X-100 and analyzed by western blotting with anti-p62 and anti-STX17. LC3B processing was used to monitor autophagy induction. The percentage of protease protection for FIP200, in comparison to p62 and STX17 is plotted on the right. Average protection and SD for n = 3 are shown. Protease protection in starved cells treated with wortmannin and/or bafilomycin are shown in Figure S3D.

See also Figure S3.

unit, six FIP200 CTR molecules formed three almost identical dimers (Figures S4B and S4C).

A monomer of the FIP200 CTR comprises an N-terminal extended helix of 29 amino acids and a C-terminal globular domain of 100 amino acids to which we refer as the "Claw" (Figure 4A). The connecting linker between the helix and the Claw is resolved in two out of six monomers. Accordingly, the Claw shows some flexibility relative to the helix (Figure S4C). The six

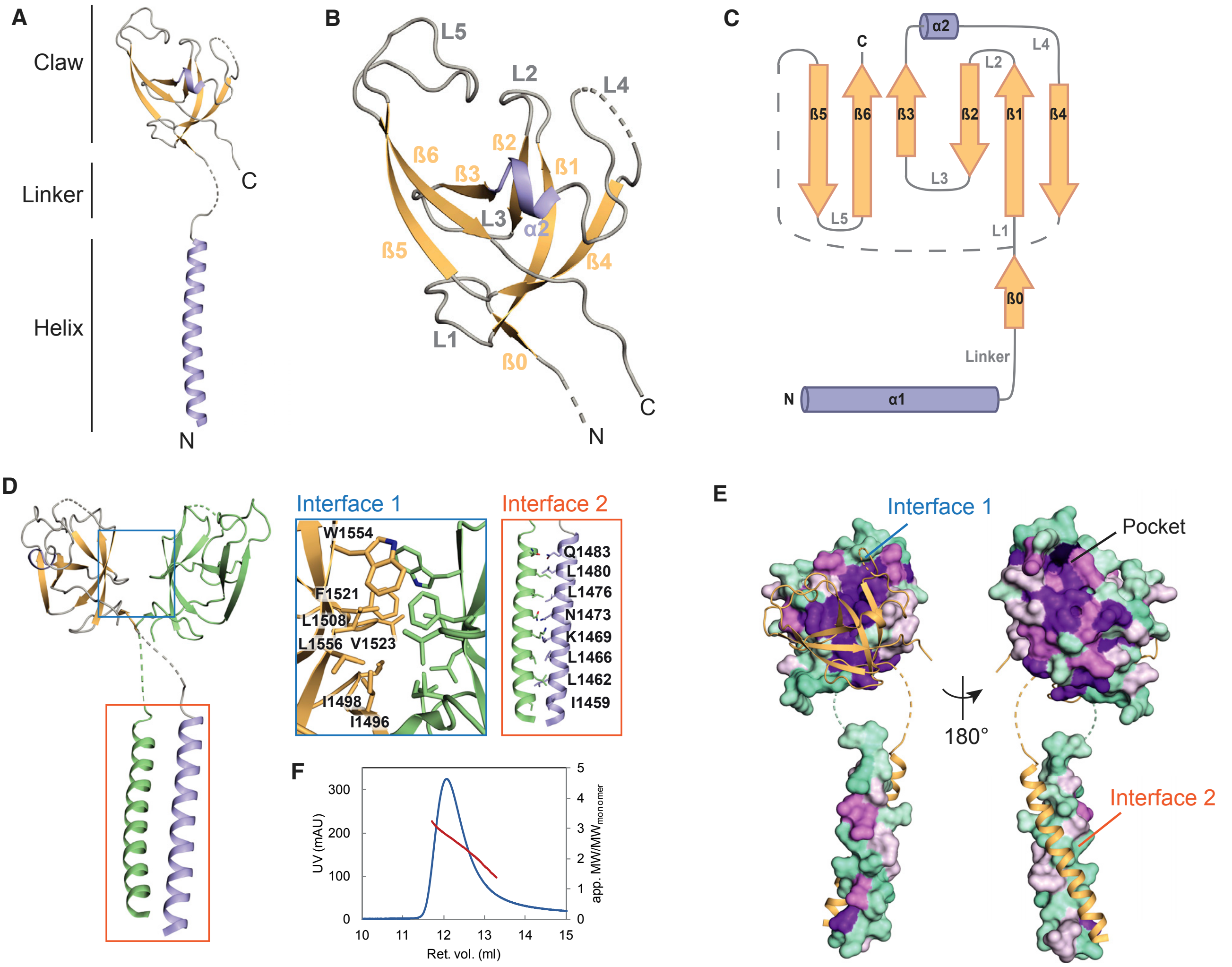

Figure 4. Crystal Structure of FIP200 CTR

(A) Crystal structure of the FIP200 CTR monomer. The molecule comprises a helix, a linker, and a globular Claw domain. The structure is colored according to its secondary structure elements (helix, purple; β strands, orange; loops, gray).

(B) Close-up of the Claw domain. The fold resembles a Claw with the β sheet being the palm of the Claw and loops L2, L4, and L5 flexed fingers. Same view and coloring used in (A).

(C) Topology plot of monomeric FIP200 CTR. α helices are shown as purple cylinders and β strands as orange arrows.

(D) Dimerization of FIP200 CTR. The homodimer is formed via two interfaces: interface 1 (blue box) and interface 2 (red box). Monomers are colored in orange and green. Interface residues are shown as sticks and labeled for only one monomer.

(E) Surface conservation plot of the FIP200 CTR monomer based on the sequence alignment of 11 different species (Figure S5B). Conserved residues are colored in purple and non-conserved residues in cyan. The second monomer is shown in cartoon representation.

(F) Analytical size-exclusion chromatography coupled to right-angle light scattering. Absorption at 280 nm (blue) and oligomeric state (as apparent molecular weight, divided by molecular weight of the monomer) (red) are plotted against the retention volume (mL).

See also Figures S4 and S5.

monomeric Claws in the asymmetric unit superimpose almost perfectly, with a root mean square deviation (rmsd) of their Cα atoms of 0.33 Å (Figure S4D).

The Claw is constituted of a six stranded, mostly antiparallel β sheet and a short α-helix (Figures 4B and 4C). Three relatively long loops are located on the same side of the β sheet in a way that the sheet resembles a palm and the loops flexed fingers of the Claw. Using PDBeFold (Krissinel and Henrick, 2007), we found that the Claw belongs to the oligonucleotide/oligosaccharide binding fold (OB-fold) (Mihailovich et al., 2010). Within this family, the FIP200 Claw domain is most similar to cold shock domains (Figures S4E and S4F) (Schindelin et al., 1993). Notably, the Claw domain did not display any structural similarity to the so-far known LIR-binding domain, the ubiquitin-related Atg8 fold (Figure S4G).

Homodimerization of FIP200 CTR is mediated by the Claw (interface-1) and the N-terminal helices that form a coiled-coil (interface-2). The linkers cross each other in such a way that

Table 1. Data Collection Statistics and Refinement

	FIP200-CTR (aa 1458–1594)		FIP200-Claw (aa 1494–1594)
	SeMet-SAD	Native	SeMet-SAD
Data Collection			
Space group	$P2_12_12$	$P2_12_12$	$C222_1$
Cell dimensions			
a, *b*, *c* (Å)	92.46, 188.68, 55.74	92.045, 187.166, 55.343	30.78, 89.22, 80.10
α, β, γ (°)	90, 90, 90	90, 90, 90	90, 90, 90
Resolution (Å)	50.00–3.45 (3.54–3.45)	47.64–3.17 (3.36–3.17)	44.63–1.56 (1.62–1.56)
R_{meas} (%)	12.6 (209.7)	8.1 (230.3)	3.8 (25.8)
$I/\sigma I$	19.74 (1.88)	11.02 (0.56)	22.1 (3.9)
Completeness (%)	100 (99.9)	97.1 (98.4)	99.10 (95.20)
Redundancy	24.94	3.66	2
$CC_{1/2}$ (%)	99.9 (81.2)	99.9 (23.9)	99.9 (86.5)
Refinement			
Resolution (Å)	–	37.76–3.20	19.49–1.56 (1.62–1.56)
No. of reflections	–	15,973	15,834
R_{work}/R_{free} (%)	–	26.65/29.49	20.77/24.02
No. of protein atoms	–	5,956	758
B factors protein (Å^2)	–	154	25.40
Ramachandran plot			
Favored (%)	–	95.2	98
Allowed (%)	–	4.8	2.0
Outliers (%)	–	0	0
RMS deviations			
Bond lengths (Å)	–	0.004	0.006
Bond angles (°)	–	0.820	0.820

Values in parentheses are for the highest-resolution shell.

the Claw of one monomer sits on top of the coiled-coil helix of the second monomer. Dimerization buries an extensive surface area of 1,440 Å^2, suggesting a physiologically plausible assembly. Both interfaces comprise mostly hydrophobic interaction areas (Figures 4D and S5A). In the Claw, a single β strand, β0, contacts β0 of the opposing monomer in interface-1. In addition, several side chains outside β0 mediate dimerization. This interface is highly conserved in different species (Figures 4E and S5B). Along with these results, analytical size exclusion chromatography coupled to right-angle light scattering confirmed the dimeric nature of FIP200 CTR (Figure 4F).

We also determined the crystal structure of the isolated Claw domain without the adjacent coiled-coil and obtained higher resolution diffraction from this material (Figure 5A). Crystals of the isolated Claw diffracted to 1.56 Å, permitting a precise characterization of side-chain conformations and ions and waters of solvation. The isolated Claw crystallized with a monomer in the asymmetric unit; however, the unit cell contains a crystallographic 2-fold-related molecule that interacts through interface-1. The preservation of interface-1 in two independently determined crystal structures obtained with different constructs and in different space groups is consistent with the functional importance of the interface-1-linked dimer.

Three sulfate ions from the crystallization medium were bound to the isolated Claw monomer at a location distal to the dimer interface-1 but overlapping with the putative FIR binding pocket (Figure 5A). These sites are of interest as sulfates ions of crystallization often serve as markers of biologically relevant phosphate binding sites. The three sulfates are very close to one another, at 6.0, 9.2, and 8.9 Å apart, respectively. Sulfate 1 is coordinated by the main chain amide and the side-chain hydroxyl of S1532 and the guanidino groups of R1573 and R1584. Sulfate 2 is also coordinated by the side chains of R1573 and R1584. Sulfate 3 is coordinated by both the main-chain NH and side chain of R1573 and is near the side chain of K1569. As compared to the structure of the CTR, which does not contain bound sulfate ions, R1573 moves by about 4 Å to engage the ions, while other residues in the pocket only move minimally. The proximity of these three sulfates was suggestive of a potential binding site for a tris-phosphorylated peptide segment, such as perhaps the tri-phosphorylated S365-S370 segment of p62.

The putative polyphosphopeptide binding sequence is located next to a hydrophobic pocket that is formed where the extended loop L5 folds back to the β sheet by hydrophobic stacking interactions between F1574 (L5), F1582 (β6), and F1529 (β3). Collectively, these Phe and other nearby hydrophobic residues join the basic residues described above to form a conserved pocket with deep

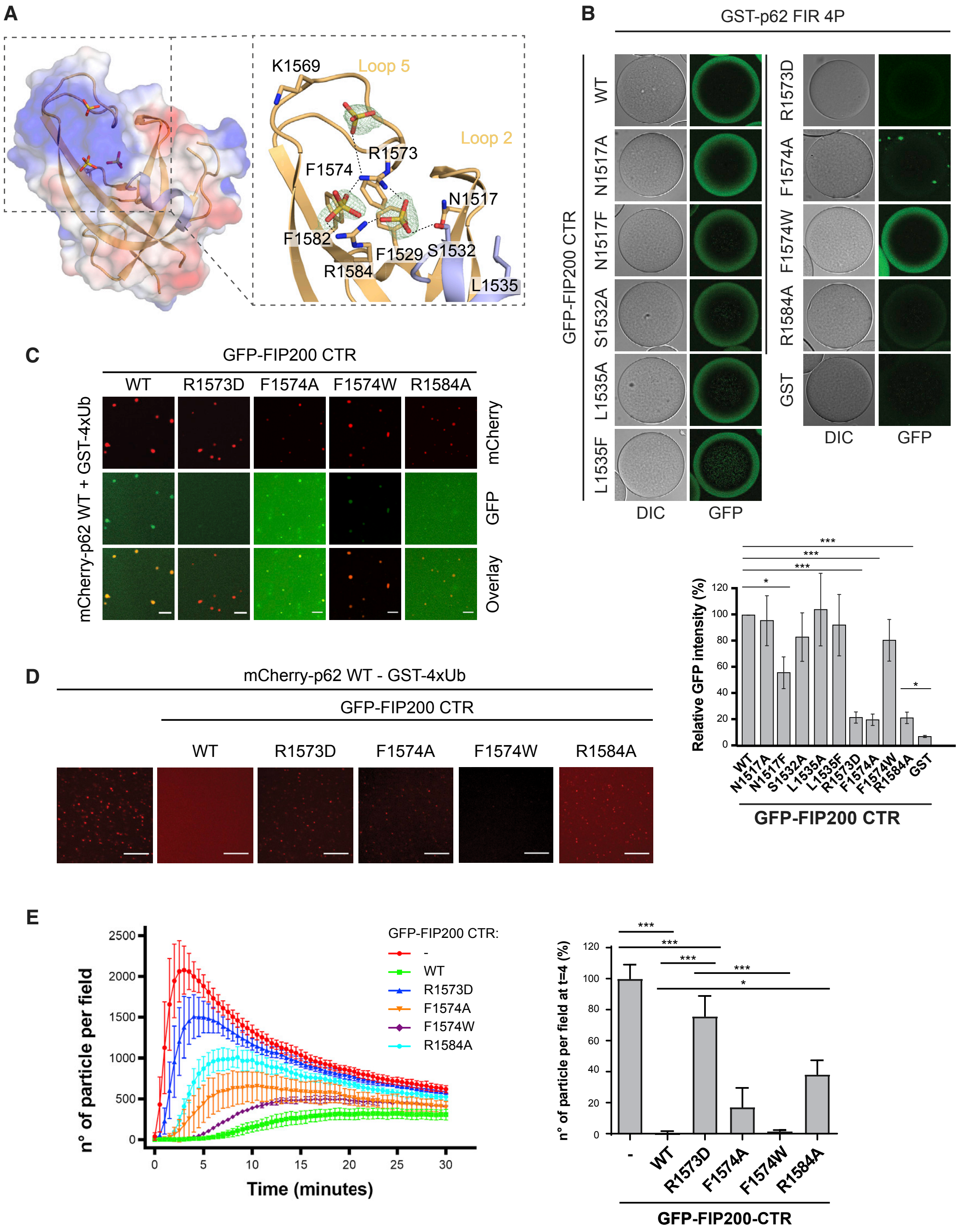

Figure 5. p62 LIR Motif Binding Depends on a Positively Charged Pocket in FIP200 CTR

(A) Electrostatic surface potential of the FIP200 Claw domain. Positively and negatively charged surfaces are colored in blue and red, respectively. The coordination of sulfate ions and amino acids of interest are shown as sticks.

(B) GSH beads were coated with GST-p62 FIR 4P, incubated with the indicated GFP-FIP200 CTR (aa 1458–1594) mutants and imaged by microscopy. For each sample the GFP intensity was normalized to the signal of GFP-FIP200 CTR WT on GST-p62 FIR 4P-coated beads. Average intensity and SEM for n = 3 are shown. Significant differences are indicated with * when p value ≤ 0.05, ** when p value ≤ 0.01, and *** when p value ≤ 0.001. Protein inputs are shown in Figure S5C.

(C) mCherry-p62 (2 μM) was incubated with GST-4x ubiquitin (10 μM) to form condensates in solution. Pre-formed condensates were incubated with 1 μM GFP-FIP200 CTR (aa 1458–1594). The recruitment of GFP-FIP200 CTR to p62-ubiquitin clusters was monitored by confocal microscopy. Scale bar, 5 μm.

(legend continued on next page)

hydrophobic recesses and more exposed phosphate binding sites. This pocket thus appeared to have all the properties expected for binding to a sequence containing both a hydrophobic motif and multiple phosphorylation sites.

Point Mutations in the FIP200 CTR Affect p62 Binding

To verify whether the identified pocket in the Claw would contribute to the p62 binding site, we introduced mutations that neutralize the positive charge of the pocket, reduce its hydrophobicity or sterically block it. The mutant FIP200 CTRs were fused to GFP and tested for their recruitment to beads coated with the p62 FIR. To render the interaction more robust, we used the 4P mutant version of the p62 FIR. Most of the FIP200 mutants bound the p62 FIR to a similar degree as the WT FIP200 CTR (Figures 5B and S5C). However, N1517F showed decreased binding while the interaction of F1574A, R1584A, and R1573D mutants was almost completely abolished. Further analysis by SPR supported our finding that the R1573D mutation strongly reduced FIP200 CTR binding to both p62 FIR WT and 4P (Figure S5D). All four residues that, when mutated, affected the binding are located in close proximity to each other around the pocket. We conclude that this pocket is a likely binding site for the p62 FIR region.

We have recently shown that p62 and certain ubiquitinated proteins phase separate into larger condensates *in vitro* that recruit LC3B via the LIR motif (Zaffagnini et al., 2018). We therefore asked if the FIP200 CTR would also be recruited to pre-formed p62-ubiquitin condensates. Indeed, we detected robust recruitment of the WT GFP-FIP200 CTR and the F1574W mutant, whereas the recruitment of the R1573D and R1584A mutants was severely impaired or completely abolished (Figure 5C). The F1574A mutant of FIP200 CTR was also recruited to p62-ubiquitin condensates although it showed a tendency to aggregate by itself in solution (Figures 5B and 5C). We also noticed a marked inhibition of the phase separation reaction in presence of the WT and F1574W GFP-FIP200 CTRs (Figures 5D, 5E, and S5E). This may involve masking of the p62 LIR motif, which is required for efficient phase separation (Zaffagnini et al., 2018). In contrast, the R1573D and R1584A mutants, which are not efficiently recruited to the clusters, did not inhibit the reaction strongly, suggesting that the inhibition of the clustering reaction by the FIP200 CTR is due to its specific interaction with p62.

FIP200 and p62 Colocalize and Interact in Cells

According to the biochemical and structural data, p62 and FIP200 are likely interacting in a transient manner during the initial stages of autophagosome formation. Therefore, we asked if they also colocalize *in vivo*. To address this, we stained for endogenous p62 and FIP200 in HAP1 cells by immunofluorescence. Indeed, we observed some p62 puncta colocalizing with FIP200, consistent with previous work conducted with HeLa cells (Itakura and Mizushima, 2011). Colocalization became more evident upon treatment with wortmannin, which blocks autophagy at a stage before isolation membrane elongation (Figure 6A). Similarly, we observed an increased p62-FIP200 colocalization in ATG7KO cells, where autophagy is blocked at a stage preceding LC3B lipidation (Figures 6A and S6A). We then used cells in which endogenous p62 is fused to a Strep-TEV-GFP tag at its N terminus (STG-p62) and analyzed the p62-FIP200 colocalization upon bafilomycin treatment. Under this condition, the colocalization of FIP200 and p62 is reduced (Figure 6B). These experiments suggest that FIP200 is recruited to p62 condensates early during autophagosome formation and dissociates from them downstream of LC3B lipidation. To determine the interaction between endogenous FIP200 and p62, we C-terminally tagged FIP200 with Strep-TEV-GFP tag (FIP200-STG) using CRISPR/Cas9. The C-terminal tagging of FIP200 did not abolish autophagy in these cells, even though the levels of the fusion protein were reduced (Figure S6B). Consistent with our biochemical results, p62 was detectable in the affinity purified fraction (Figures 6C and S6C). The same beads were analyzed by mass spectrometry and all the components of the ULK1 complex were found to interact with FIP200 (Table S1). We then asked whether mutations in the FIP200 Claw domain affected the binding to endogenous p62. GFP-FIP200 CTR WT or the mutants were used as baits to pull down p62 from cell lysates. As observed for the recombinant proteins (Figures 5B and 5C), the FIP200 R1573D mutant did not interact with p62, unlike WT FIP200 and the FIP200 F1574W mutant (Figure 6D). To test whether mutations in full-length FIP200 would also affect binding to p62, we transfected HeLa cells with various HA-tagged FIP200 variants. We then used the HA tag to co-immunoprecipitate FIP200 and p62 from cell lysates. In accordance with the pull-down experiment (Figure 6D), p62 was efficiently co-immunoprecipitated by WT FIP200, while binding was very weak for the R1573D and the ΔCTR mutants (Figure 6E). The F1574W mutant also showed a markedly reduced interaction with p62 in this assay, suggesting that additional interactions are required for efficient co-precipitation of p62 and FIP200.

In vitro the FIP200-p62 interaction is dependent on the p62 LIR motif. Therefore, we asked if mutating the LIR motif in endogenous p62 would affect its binding to FIP200 CTR. To this end, we used lysates from STG-p62 cells in which the p62 LIR motif was mutated (STG-p62 LIRmut) in a pull-down experiment with GST-FIP200 CTR as bait. Indeed, the recruitment of p62 LIRmut to FIP200 CTR was significantly reduced (Figure 6F). To dissect the function of the FIP200 Claw domain in cells, we generated cells in which the Claw of endogenous FIP200 was deleted by CRISPR/Cas9 (FIP200ΔClaw, aa1493–1594). These cells showed accumulation of p62 and reduced LC3B lipidation (Figure S7A). The expression level of the FIP200ΔClaw was lower compared to the WT proteins (Figures S7A and S7B), which was particularly evident after higher passage numbers. Therefore, we

(D) mCherry-p62 was incubated with GST-4x ubiquitin in the presence or absence of the GFP-FIP200 CTR (WT or mutants). The formation of p62-ubiquitin condensates was monitored over 30 min. Images show p62-ubiquitin clusters at t = 4 min. Scale bar, 25 μm. Protein inputs are shown in Figure S5E.

(E) Quantifications of the experiment in (D). For each sample, the number of particles per field is plotted against time (left). Number of particles per field at t = 4 min is plotted on the right. For each sample, the particle number was normalized to the average number of p62-ubiquitin clusters formed in absence of FIP200 CTR. Averages and SEM for $n \geq 3$ are shown. Significant differences are indicated with * when p value ≤ 0.05, ** when p value ≤ 0.01, and *** when p value ≤ 0.001. See also Figure S5.

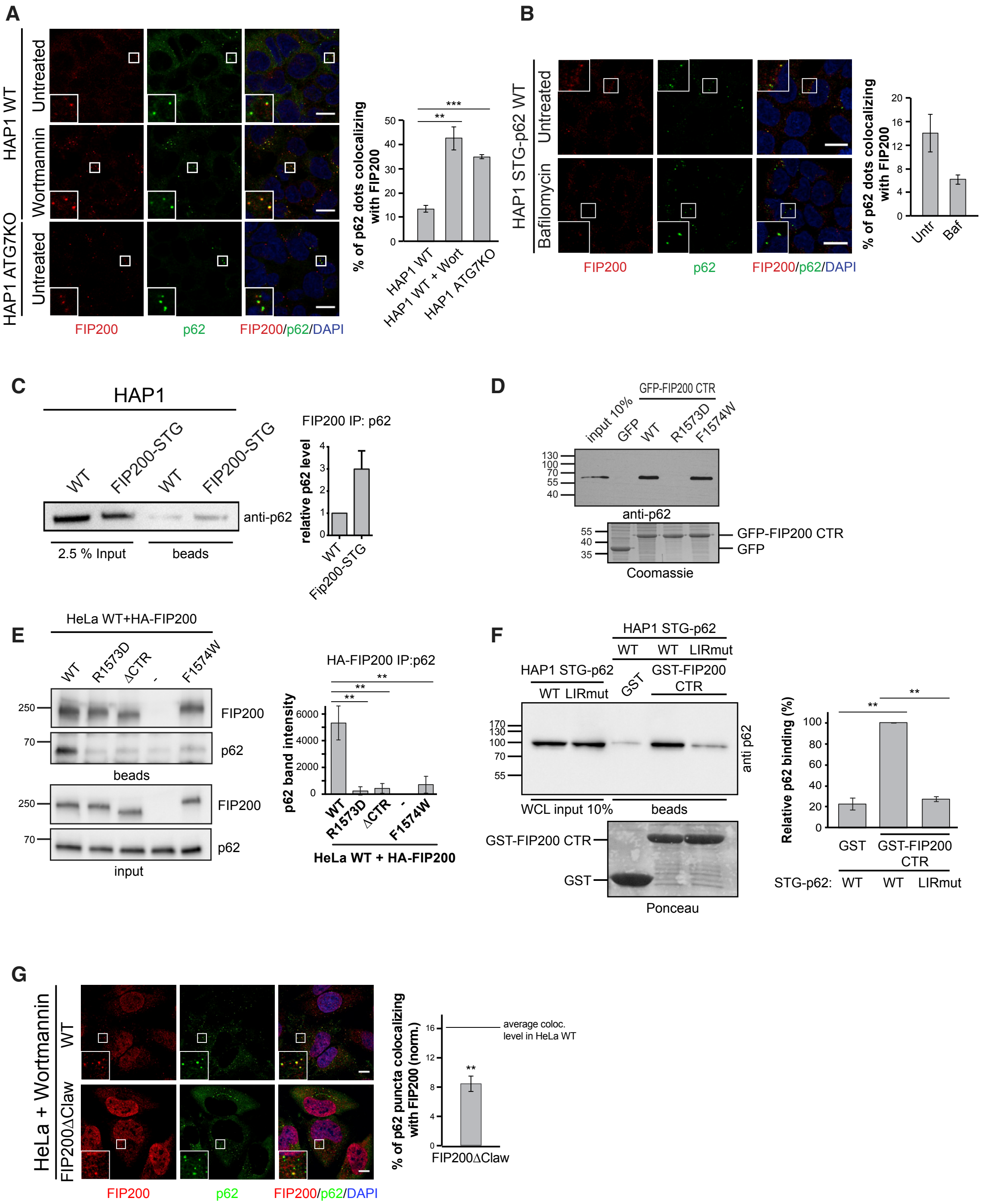

Figure 6. p62-FIP200 Interaction in Cells

(A) Colocalization analysis of p62 and FIP200 in HAP1 cells (WT or ATG7KO) left untreated or treated with wortmannin (1 μM) for 1 h. Endogenous p62 and FIP200 were detected by immunofluorescence. Scale bar, 10 μm. Average percentages of colocalization and SEM for n = 3 are shown. Significant differences are indicated with * when p value ≤ 0.05, ** when p value ≤ 0.01, and *** when p value ≤ 0.001.

(B) Immunofluorescence of p62 and FIP200 in HAP1 STG-p62 cell line left untreated or treated with bafilomycin (400 nM) for 1 h. p62 was detected through the GFP tag fused to the endogenous protein and FIP200 was detected by immunofluorescence. Scale bar, 10 μm. Average percentages of colocalization and SEM (n = 2) are shown. Significant differences are indicated with * when p value ≤ 0.05, ** when p value ≤ 0.01, and *** when p value ≤ 0.001.

(C) The C terminus of endogenous FIP200 was tagged in HAP1 cells with GFP-TEV-Strep (FIP200-STG). Affinity purification was performed using HAP1 WT or FIP200-STG cells and the bound material was analyzed by western blotting with anti-p62. The intensities of the p62 bands were normalized for the total level of p62 in the lysate (input). Average p62 levels and SD for n = 4 are shown. Three additional replicates of the immunoprecipitation are shown in Figure S6C.

(legend continued on next page)

conducted the following experiments with low passage number cells. We also compared the autophagic activity of higher passage number FIP200ΔClaw cells, where the protein expression is severely reduced, with cells expressing a corresponding amount of full-length FIP200 and found that the autophagic defect in the FIP200ΔClaw cells is not merely due to the reduced protein expression (Figure S7C).

We then analyzed the colocalization of p62 and FIP200 in WT and FIP200ΔClaw cells treated with wortmannin to accumulate FIP200 at p62 condensates, facilitating its detection. The recruitment of FIP200 to p62 condensates is significantly reduced in FIP200ΔClaw cells when compared to cells expressing WT FIP200 (Figure 6G).

Deletion of the FIP200 Claw in HeLa Cells Impairs the Selective Degradation of Condensates Containing Ubiquitinated Proteins and p62

Next, we set out to better understand the functional role of FIP200 and its Claw domain in selective autophagy. Immunofluorescence staining of p62 and ubiquitin in HeLa cells showed that p62 colocalized with ubiquitin-positive condensates and that FIP200ΔClaw cells showed a higher number of p62 and ubiquitin puncta/cell when compared to WT cells. We also observed an increased volume of the p62 condensates in the FIP200ΔClaw cells compared to WT (Figure 7A). In FIP200ΔClaw cells, colocalization between p62 and LC3B was significantly lower than in WT cells (Figure 7B), supporting the idea that FIP200 plays a role in the early stages of aggrephagy. When cells were treated with bafilomycin, the differences in the number of p62 puncta and the degree of p62-LC3B colocalization became smaller compared to untreated cells (Figures 7A and 7B), whereas we observed a further increase in the volume of p62 condensates (Figure 7A). This suggests that deletion of the FIP200 Claw domain does not completely inhibit the delivery of p62-positive cargo into the lysosome, but slows the initial stages of aggrephagy. To test whether the Claw mediated the recruitment of ATG8 proteins to the cargo, we performed immunofluorescence staining of ubiquitin and GABARAP. The number of ubiquitin puncta colocalizing with GABARAP was significantly decreased in FIP200ΔClaw cells when compared to WT cells (Figure 7C). This difference in colocalization became smaller when cells were treated with bafilomycin, again, suggesting that aggrephagy is not fully blocked (Figure 7C). Our results were recapitulated in cells in which FIP200 was knocked out by small interfering RNA (siRNA) (Figures 7D and S7D).

Next, we assessed at which step FIP200 is required for the autophagy of the p62-ubiquitin condensates. We first tested if ULK1 was still found in p62-ubiquitin condensates in the absence of FIP200. Surprisingly, ULK1 was recruited to p62 puncta when FIP200 was knocked down (Figure 7E, left). FIP200 has been shown to interact with ATG16L1 and to recruit it to the site of autophagosome formation under starvation conditions (Gammoh et al., 2013; Nishimura et al., 2013). Therefore, we tested whether ATG16L1 was still recruited to p62 puncta when FIP200 was depleted. Indeed, and in contrast to ULK1, the recruitment of ATG16L1 to p62 was undetectable when FIP200 was knocked down (Figure 7E, right).

We then went on to assess the evolutionary conservation of the interaction by studying the interaction of yeast Atg19 with Atg11. Atg19 was robustly co-precipitated by full-length Atg11, but not by a deletion mutant lacking the CTR (Figure S7F). Moreover, an Atg11Δ strain transformed with Atg11 ΔCTR showed impaired Ape1 processing, in particular in nutrient-rich conditions, where the Cvt pathway is the predominant pathway for prApe1 delivery into the vacuole (Figure S7G). This suggests that Atg11 CTR-Atg19 interaction is necessary for the correct progression of selective autophagy in yeast.

DISCUSSION

The p62 cargo receptor mediates the phase separation of ubiquitinated proteins into larger condensates and their subsequent tethering to the autophagosomal membrane via its interaction with membrane localized ATG8 proteins. Our discovery that p62 directly interacts with FIP200 shows that it has an additional function in the generation of an ATG8 protein-decorated isolation membrane in the vicinity of the cargo. The coordination of these events appears to be centered around a disordered region of p62 comprised of residues 326–380 (FIR), which also contains the LIR motif. This region directly interacts with the Claw of FIP200, which is highly conserved in evolution and is also present in the otherwise non-homologous *S. cerevisiae* Atg11.

The mutually exclusive interaction of p62 with FIP200 and ATG8 proteins may provide an inbuild directionality in the system (Figure 7F). FIP200 is an early acting component aiding the recruitment and activation of the autophagy machinery, which, in turn, recruits the ATG8 conjugation machinery culminating in the catalytic conjugation of LC3B and other ATG8 proteins to the nascent autophagosomal membrane. In this manner they accumulate to very high local concentrations on the surface of

(D) GFP-Trap beads coated with GFP or GFP-FIP200 CTR (aa 1458–1594) variants were incubated with cell lysates and analyzed by western blotting. Loading control of the bait proteins is shown below the blot.

(E) Anti-HA co-immunoprecipitation in HeLa cells transfected with HA-FIP200. The intensities of the p62 bands were measured and normalized to the amount of the respective bait (HA-FIP200). Average band intensities and SEM (n = 3) are shown. Significant differences are indicated with * when p value $\leq$ 0.05, ** when p value $\leq$ 0.01, and *** when p value $\leq$ 0.001.

(F) Pull-down of p62 WT and LIRmut from HAP1 STG-p62 cell lysates was performed as in Figure 1B using GST/GST-FIP200 CTR as bait. Loading control of the bait proteins is shown below the blot. Band intensities were measured and normalized to the intensity of p62 WT binding to FIP200 CTR WT. Average band intensities and SEM (n = 3) are shown. Significant differences are indicated with * when p value $\leq$ 0.05, ** when p value $\leq$ 0.01, and *** when p value $\leq$ 0.001.

(G) The Claw domain of endogenous FIP200 was deleted in HeLa cells by CRISPR/Cas9 (HeLa FIP200ΔClaw). Cells were treated with wortmannin (1 μM) for 3 h, and p62 and FIP200 were detected by immunofluorescence. Scale bar, 10 μm. The percentage of p62 puncta colocalizing with FIP200 in FIP200ΔClaw cells is compared to the average level of colocalization in WT cells. Average colocalization and SEM for n = 3 is shown. Significant differences are indicated with * when p value $\leq$ 0.05, ** when p value $\leq$ 0.01, and *** when p value $\leq$ 0.001.

See also Figure S6.

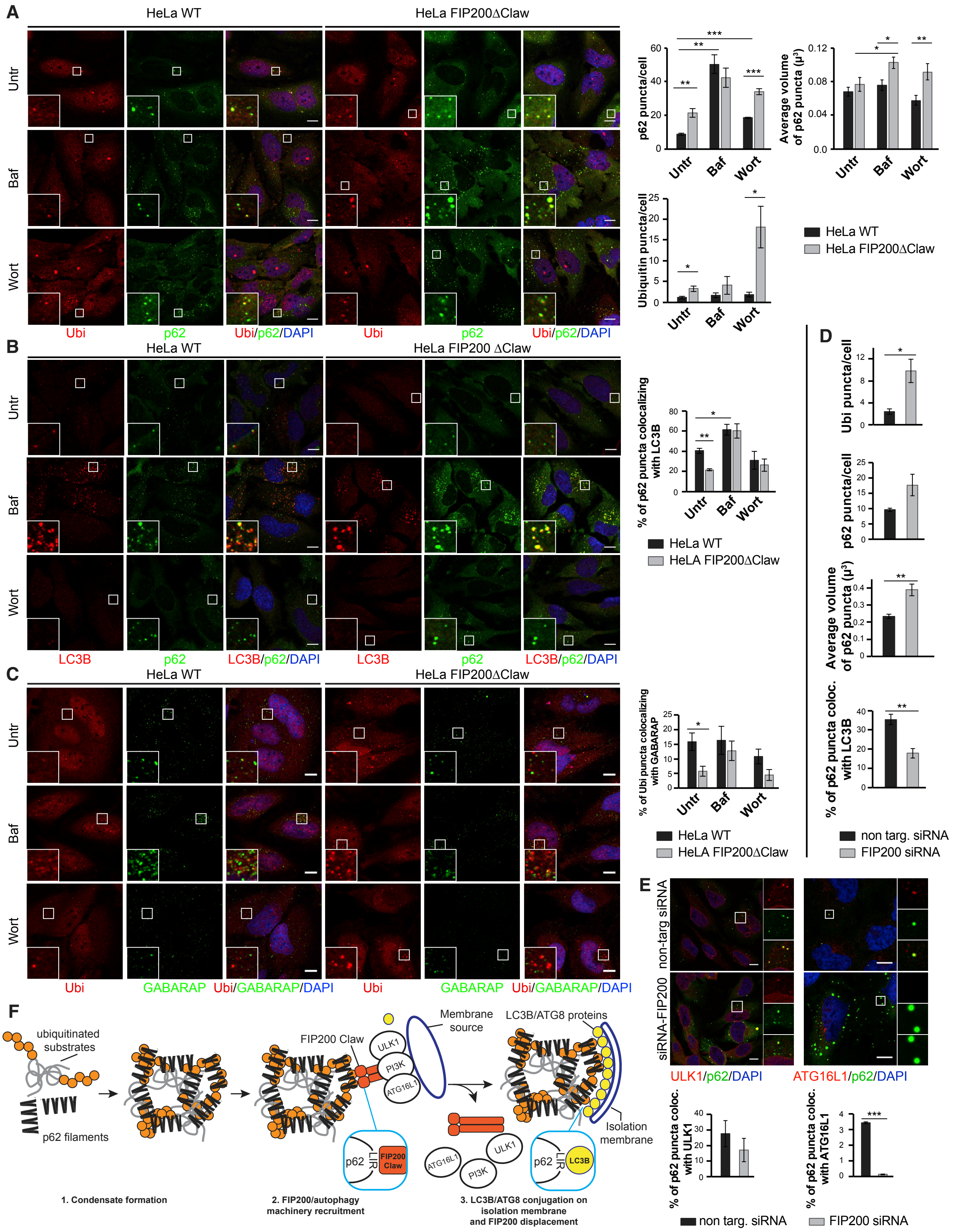

(legend on next page)

the membrane and may therefore be able to displace FIP200 from the cargo material because the binding sites of FIP200 and LC3B on p62 are overlapping.

The interaction between p62 and the FIP200 CTR bears some similarities with the recently reported interaction of CCPG1 with the C-terminal part of FIP200 (Smith et al., 2018). However, even though the FIR in CCPG1 has some resemblance with the LIR motif of p62, there are also notable differences in the two interactions as the FIRs of CCPG1 do not overlap with its LIR motif. It thus appears that the LC3B and FIP200 binding sites of p62 are fused into one region in order to enable it to drive the progression from phase separation to autophagy machinery recruitment and finally membrane enclosure.

In cells, the coordination of these events and in particular FIP200 recruitment may require phosphorylation of the p62 FIR on multiple residues since phospho-mimicking mutation of residues that we found to be phosphorylated *in vivo*, enhances the binding of p62 to the FIP200 Claw. S365, S366 and S370 are located in a region that shows similarity with yeast Atg19 and phosphorylation of the corresponding residues increase its binding to Atg11 (Pfaffenwimmer et al., 2014) revealing a remarkable conservation of this mode of interaction. In addition, it was previously shown that phosphorylation of S349 in p62 (Tanji et al., 2014), is stimulated by protein aggregation. The kinases mediating the phosphorylation of the residues in the p62 condensates to promote the FIP200 interaction remain to be identified. Other aspects of p62 function are also regulated by phosphorylation. For example, phosphorylation of S403, within the p62 UBA domain, by TBK1 increases the affinity of p62 for ubiquitin and promotes p62-ubiquitin condensates formation (Matsumoto et al., 2011, 2015).

FIP200 is a part of the ULK1 complex, the activity of which is essential for autophagosome nucleation (Hara et al., 2008). Similarly, Atg11 binds the Atg1 kinase complex, the equivalent of ULK1 in yeast (Kim et al., 2001). We found that the recruitment of ULK1 to p62-ubiquitin condensates is FIP200-independent and may be mediated by additional factors that contribute to the p62-mediated ubiquitin phase separation and autophagy (Clausen et al., 2010; Filimonenko et al., 2010; Liu et al., 2017; Rui et al., 2015). We further show that the recruitment of ATG16L1 to p62-ubiquitin condensates is abolished in FIP200-depleted cells providing a mechanistic basis for the block of autophagy of the p62-ubiquitin condensates upon FIP200 depletion. However, FIP200 may also be required for events upstream of ATG16L1 recruitment. ULK1 activation requires FIP200 at least under starvation conditions (Hara et al., 2008) and therefore the interaction of p62 with the FIP200 Claw may aid the activation of ULK1 at ubiquitin-positive condensates to initiate autophagosome formation. In addition, FIP200 may be required for early membrane remodeling events, similar to yeast Atg17 (Bahrami et al., 2017).

The crystal structures show that the FIP200 Claw and the larger CTR are stable dimers even in the absence of phospho-p62 FIR. Thus, induced dimerization of FIP200 is probably not responsible for signaling to ULK1. The function of Claw dimerization may rather be to increase the avidity of the p62-Claw interaction at p62-ubiquitin condensates, which is weak at the monomer-monomer level. Similarly, CCPG1 has two FIR motifs which may bind individually to each Claw domain within the dimer (Smith et al., 2018). In the absence of a crystal structure of the phospho-FIR Claw complex, it is not possible to say if conformational changes in the Claw and/or CTR dimer are induced. While p62 binding is not required to induce dimerization, it is still possible that subtle conformational changes in the dimer could take place. Such changes might potentially regulate the ability of the larger FIP200 structure to recruit and activate ULK1 and/or to remodel membranes. Taken together, our study reveals a long-sought link between cargo condensation and autophagic degradation in p62 mediated autophagy, represented by an interaction of the FIP200 Claw domain with p62.

STAR★METHODS

Detailed methods are provided in the online version of this paper and include the following:

- KEY RESOURCES TABLE
- CONTACT FOR REAGENT AND RESOURCE SHARING
- EXPERIMENTAL MODEL AND SUBJECT DETAILS
  - Cell lines and cell culture

Figure 7. Deletion of the FIP200 Claw in HeLa Cells Inhibits the Processing of p62-Ubiquitin Condensates by Autophagy

(A) WT or FIP200ΔClaw HeLa cells were left untreated or treated with bafilomycin (400 nM for 3 h) or wortmannin (1 μM for 3 h). Immunofluorescence for ubiquitin and p62 was performed. Scale bar, 10 μm. The number and volume of p62 puncta/cell and the number of ubiquitin puncta/cell were measured and plotted on the right. The average volume of p62 puncta was derived from the particle size. Average number and volume of puncta and SEM are shown (number of p62 and ubiquitin puncta: n = 3; volume of p62 condensates: n = 6). Significant differences are indicated with * when p value $\leq$ 0.05, ** when p value $\leq$ 0.01, and *** when p value $\leq$ 0.001.

(B) Immunofluorescence for LC3B and p62 was performed in HeLa cells (WT or FIP200ΔClaw) treated like in (A). Scale bar, 10 μm. The percentage of p62 puncta colocalizing with LC3B is shown on the right. Average colocalization and SEM for n = 3 are shown. Significant differences are indicated with * when p value $\leq$ 0.05, ** when p value $\leq$ 0.01, and *** when p value $\leq$ 0.001.

(C) Immunofluorescence for ubiquitin and GABARAP and colocalization analysis in HeLa WT and FIP200ΔClaw as described in (B). Scale bar, 10 μm. Significant differences are indicated with * when p value $\leq$ 0.05, ** when p value $\leq$ 0.01, and *** when p value $\leq$ 0.001.

(D) HeLa cells were treated with non-targeting siRNA or FIP200 siRNA. FIP200 depletion was monitored by western blotting (Figure S7D). After immunofluorescence staining of p62 and ubiquitin or p62 and LC3B (Figure S7D), number and volume of p62 puncta, number of ubiquitin puncta and colocalization of ubiquitin-LC3B puncta were analyzed as described in (A) and (B). Averages and SEM for n = 3 are shown in every plot except for the measure of p62 condensates volume, where n = 6. Significant differences are indicated with * when p value $\leq$ 0.05, ** when p value $\leq$ 0.01, and *** when p value $\leq$ 0.001.

(E) Immunofluorescence staining of HeLa cells treated with non-targeting siRNA or FIP200 siRNA. Cells were stained either with p62 and ULK1 antibodies (left) or with p62 and ATG16L1 antibodies (right; additional images in Figure S7E). Scale bar, 10 μm. The efficiency of FIP200 siRNA was assessed by western blotting (Figure S7E). The percentages of p62 puncta colocalizing with ULK1 (left) or ATG16L1 (right) are plotted. Average colocalization and SEM for n = 2 is shown. Significant differences are indicated with * when p value $\leq$ 0.05, ** when p value $\leq$ 0.01, and *** when p value $\leq$ 0.001.

(F) Mutual interaction of p62 with FIP200 and LC3B may provide an inbuilt directionality in autophagy.

See also Figure S7.

- METHOD DETAILS
  - Protein expression and purification
  - Microscopy-based protein-protein interaction assays
  - p62 pull-down from HeLa and HAP1 cell lysates
  - Identification of phosphorylated residues by mass spectrometry
  - Surface plasmon resonance spectroscopy
  - Protease protection assay
  - Crystallization and structure determination
  - Right-angle light scattering
  - p62 aggregation assay
  - Immunocytochemistry
  - Cell lysis and western blotting
  - Affinity co-purification of FIP200 and p62 from HAP1 FIP200-STG cells
  - HA-FIP200 – p62 co-immunoprecipitation from HeLa cells
  - Cell treatment with siRNA
  - Co-immunoprecipitation of yeast proteins
  - Ape1 processing assay
- QUANTIFICATION AND STATISTICAL ANALYSIS
  - Microscopy based protein-protein interaction assays
  - p62 pull-down experiments and protease protection assay
  - p62/ubiquitin puncta count and p62/LC3B colocalization analysis
  - Statistical analysis
- DATA AND SOFTWARE AVAILABILITY

SUPPLEMENTAL INFORMATION

Supplemental Information includes seven figures and one table and can be found with this article online at https://doi.org/10.1016/j.molcel.2019.01.035.

ACKNOWLEDGMENTS

We thank X. Ren for scientific assistance. This work was supported by ERC grants (646653 to S.M.; 616024 to O.D.), the Austrian Science Fund (FWF) (P30401-B21 and W1261) (to S.M.), NIH (R01 GM111730) (to J.H.H.), the Human Frontiers Science Program (RGP0026/2017) (to J.H.H. and S.M.), and an OEAW Doc fellowship (to C.A.). Beamline 8.3.1 at the Advance Light Source is operated by the University of California Office of the President, Multicampus Research Programs and Initiatives (MR-15-328599), the National Institutes of Health (R01 GM124149 and P30 GM124169), Plexxikon Inc., and the Integrated Diffraction Analysis Technologies program of the U.S. Department of Energy Office of Biological and Environment Research. The Advanced Light Source (Berkeley, CA) is a national user facility operated by Lawrence Berkeley National Laboratory on behalf of the U.S. Department of Energy (contract number DE-AC02-05CH11231), Office of Basic Energy Sciences. We thank the MFPL Mass Spectrometry Facility for technical support and the VBCF for providing the MS instrument pool. Diffraction data also have been collected on BL14.1 at the BESSY II electron storage ring operated by the Helmholtz-Zentrum Berlin. We particularly acknowledge the help and support of Manfred Weiss during the experiment. We thank Peter Seeberger, Oren Moscovitz, Paulina Kaplonek, and Amit Koikkarah Aji for providing the SPR instrument and technical support.

AUTHOR CONTRIBUTIONS

E.T., M.W., C.A., T.B.-B., M.-Y.S., R.T., M. Sztacho, A.D., X.S., G.Z., A.G., M. Schuschnig, D.F., D.B., J.R., and M.H. conducted the experiments; E.T., M.H., J.H.H., O.D., and S.M designed the experiments; and E.T., J.H.H., O.D., and S.M. wrote the paper.

CONFLICT OF INTEREST

J.H.H. is a co-founder of Casma Therapeutics. S.M. is member of the scientific advisory board of Casma Therapeutics.

Received: May 30, 2018
Revised: November 13, 2018
Accepted: January 24, 2019
Published: March 7, 2019

REFERENCES

Adams, P.D., Afonine, P.V., Bunkóczi, G., Chen, V.B., Davis, I.W., Echols, N., Headd, J.J., Hung, L.-W., Kapral, G.J., Grosse-Kunstleve, R.W., et al. (2010). PHENIX: a comprehensive Python-based system for macromolecular structure solution. Acta Crystallogr. D Biol. Crystallogr. *66*, 213–221.

Abert, C., Kontaxis, G., and Martens, S. (2016). Accessory interaction motifs in the Atg19 cargo receptor enable strong binding to the clustered ubiquitin-related Atg8 protein. J. Biol. Chem. *291*, 18799–18808.

Bahrami, A.H., Lin, M.G., Ren, X., Hurley, J.H., and Hummer, G. (2017). Scaffolding the cup-shaped double membrane in autophagy. PLoS Comput. Biol. *13*, e1005817.

Bjørkøy, G., Lamark, T., Brech, A., Outzen, H., Perander, M., Overvatn, A., Stenmark, H., and Johansen, T. (2005). p62/SQSTM1 forms protein aggregates degraded by autophagy and has a protective effect on huntingtin-induced cell death. J. Cell Biol. *171*, 603–614.

Ciuffa, R., Lamark, T., Tarafder, A.K., Guesdon, A., Rybina, S., Hagen, W.J., Johansen, T., and Sachse, C. (2015). The selective autophagy receptor p62 forms a flexible filamentous helical scaffold. Cell Rep. *11*, 748–758.

Clausen, T.H., Lamark, T., Isakson, P., Finley, K., Larsen, K.B., Brech, A., Øvervatn, A., Stenmark, H., Bjørkøy, G., Simonsen, A., and Johansen, T. (2010). p62/SQSTM1 and ALFY interact to facilitate the formation of p62 bodies/ALIS and their degradation by autophagy. Autophagy *6*, 330–344.

DeLano, W.L. (2014). The PyMOL molecular graphics system (Schrödinger LLC).

Doublie, S. (1997). Preparation of selenomethionyl proteins for phase determination. Methods Enzymol. *276*, 523–530.

Emsley, P., Lohkamp, B., Scott, W.G., and Cowtan, K. (2010). Features and development of Coot. Acta Crystallogr. D Biol. Crystallogr. *66*, 486–501.

Filimonenko, M., Isakson, P., Finley, K.D., Anderson, M., Jeong, H., Melia, T.J., Bartlett, B.J., Myers, K.M., Birkeland, H.C., Lamark, T., et al. (2010). The selective macroautophagic degradation of aggregated proteins requires the PI3P-binding protein Alfy. Mol. Cell *38*, 265–279.

Fracchiolla, D., Sawa-Makarska, J., Zens, B., Ruiter, A., Zaffagnini, G., Brezovich, A., Romanov, J., Runggatscher, K., Kraft, C., Zagrovic, B., and Martens, S. (2016). Mechanism of cargo-directed Atg8 conjugation during selective autophagy. eLife *5*, e18544.

Gammoh, N., Florey, O., Overholtzer, M., and Jiang, X. (2013). Interaction between FIP200 and ATG16L1 distinguishes ULK1 complex-dependent and -independent autophagy. Nat. Struct. Mol. Biol. *20*, 144–149.

Hara, T., Takamura, A., Kishi, C., Iemura, S., Natsume, T., Guan, J.-L., and Mizushima, N. (2008). FIP200, a ULK-interacting protein, is required for autophagosome formation in mammalian cells. J. Cell Biol. *181*, 497–510.

Hurley, J.H., and Young, L.N. (2017). Mechanisms of autophagy initiation. Annu. Rev. Biochem. *86*, 225–244.

Ichimura, Y., Kumanomidou, T., Sou, Y.S., Mizushima, T., Ezaki, J., Ueno, T., Kominami, E., Yamane, T., Tanaka, K., and Komatsu, M. (2008). Structural basis for sorting mechanism of p62 in selective autophagy. J. Biol. Chem. *283*, 22847–22857.

Itakura, E., and Mizushima, N. (2011). p62 Targeting to the autophagosome formation site requires self-oligomerization but not LC3 binding. J. Cell Biol. *192*, 17–27.

Kabsch, W. (2010). Integration, scaling, space-group assignment and post-refinement. Acta Crystallogr. D Biol. Crystallogr. *66*, 133–144.

Kamber, R.A., Shoemaker, C.J., and Denic, V. (2015). Receptor-bound targets of selective autophagy use a scaffold protein to activate the Atg1 kinase. Mol. Cell *59*, 372–381.

Khaminets, A., Heinrich, T., Mari, M., Grumati, P., Huebner, A.K., Akutsu, M., Liebmann, L., Stolz, A., Nietzsche, S., Koch, N., et al. (2015). Regulation of endoplasmic reticulum turnover by selective autophagy. Nature *522*, 354–358.

Kim, J., Kamada, Y., Stromhaug, P.E., Guan, J., Hefner-Gravink, A., Baba, M., Scott, S.V., Ohsumi, Y., Dunn, W.A., Jr., and Klionsky, D.J. (2001). Cvt9/Gsa9 functions in sequestering selective cytosolic cargo destined for the vacuole. J. Cell Biol. *153*, 381–396.

Krissinel, E., and Henrick, K. (2007). Inference of macromolecular assemblies from crystalline state. J. Mol. Biol. *372*, 774–797.

Krug, M., Weiss, M.S., Heinemann, U., and Mueller, U. (2012). XDSAPP: a graphical user interface for the convenient processing of diffraction data using XDS. J. Appl. Cryst. *45*, 568–572.

Lamark, T., Perander, M., Outzen, H., Kristiansen, K., Øvervatn, A., Michaelsen, E., Bjørkøy, G., and Johansen, T. (2003). Interaction codes within the family of mammalian Phox and Bem1p domain-containing proteins. J. Biol. Chem. *278*, 34568–34581.

Langer, G., Cohen, S.X., Lamzin, V.S., and Perrakis, A. (2008). Automated macromolecular model building for X-ray crystallography using ARP/wARP version 7. Nat. Protoc. *3*, 1171–1179.

Lazarou, M., Sliter, D.A., Kane, L.A., Sarraf, S.A., Wang, C., Burman, J.L., Sideris, D.P., Fogel, A.I., and Youle, R.J. (2015). The ubiquitin kinase PINK1 recruits autophagy receptors to induce mitophagy. Nature *524*, 309–314.

Liu, X., Li, Y., Wang, X., Xing, R., Liu, K., Gan, Q., Tang, C., Gao, Z., Jian, Y., Luo, S., et al. (2017). The BEACH-containing protein WDR81 coordinates p62 and LC3C to promote aggrephagy. J. Cell Biol. *216*, 1301–1320.

MacLean, B., Tomazela, D.M., Shulman, N., Chambers, M., Finney, G.L., Frewen, B., Kern, R., Tabb, D.L., Liebler, D.C., and MacCoss, M.J. (2010). Skyline: an open source document editor for creating and analyzing targeted proteomics experiments. Bioinformatics *26*, 966–968.

Matsumoto, G., Wada, K., Okuno, M., Kurosawa, M., and Nukina, N. (2011). Serine 403 phosphorylation of p62/SQSTM1 regulates selective autophagic clearance of ubiquitinated proteins. Mol. Cell *44*, 279–289.

Matsumoto, G., Shimogori, T., Hattori, N., and Nukina, N. (2015). TBK1 controls autophagosomal engulfment of polyubiquitinated mitochondria through p62/SQSTM1 phosphorylation. Hum. Mol. Genet. *24*, 4429–4442.

Mihailovich, M., Militti, C., Gabaldón, T., and Gebauer, F. (2010). Eukaryotic cold shock domain proteins: highly versatile regulators of gene expression. BioEssays *32*, 109–118.

Mochida, K., Ohsumi, Y., and Nakatogawa, H. (2014). Hrr25 phosphorylates the autophagic receptor Atg34 to promote vacuolar transport of α-mannosidase under nitrogen starvation conditions. FEBS Lett. *588*, 3862–3869.

Mochida, K., Oikawa, Y., Kimura, Y., Kirisako, H., Hirano, H., Ohsumi, Y., and Nakatogawa, H. (2015). Receptor-mediated selective autophagy degrades the endoplasmic reticulum and the nucleus. Nature *522*, 359–362.

Mueller, U., Förster, R., Hellmig, M., Huschmann, F.U., Kastner, A., Malecki, P., Pühringer, S., Röwer, M., Sparta, K., Steffien, M., et al. (2015). The macromolecular crystallography beamlines at BESSY II of the Helmholtz-Zentrum Berlin: current status and perspectives. Eur. Phys. J. Plus *130*, 141–150.

Nishimura, T., Kaizuka, T., Cadwell, K., Sahani, M.H., Saitoh, T., Akira, S., Virgin, H.W., and Mizushima, N. (2013). FIP200 regulates targeting of Atg16L1 to the isolation membrane. EMBO Rep. *14*, 284–291.

Pankiv, S., Clausen, T.H., Lamark, T., Brech, A., Bruun, J.A., Outzen, H., Øvervatn, A., Bjørkøy, G., and Johansen, T. (2007). p62/SQSTM1 binds directly to Atg8/LC3 to facilitate degradation of ubiquitinated protein aggregates by autophagy. J. Biol. Chem. *282*, 24131–24145.

Pfaffenwimmer, T., Reiter, W., Brach, T., Nogellova, V., Papinski, D., Schuschnig, M., Abert, C., Ammerer, G., Martens, S., and Kraft, C. (2014). Hrr25 kinase promotes selective autophagy by phosphorylating the cargo receptor Atg19. EMBO Rep. *15*, 862–870.

Rappsilber, J., Mann, M., and Ishihama, Y. (2007). Protocol for micro-purification, enrichment, pre-fractionation and storage of peptides for proteomics using StageTips. Nat. Protoc. *2*, 1896–1906.

Rui, Y.N., Xu, Z., Patel, B., Chen, Z., Chen, D., Tito, A., David, G., Sun, Y., Stimming, E.F., Bellen, H.J., et al. (2015). Huntingtin functions as a scaffold for selective macroautophagy. Nat. Cell Biol. *17*, 262–275.

Sánchez-Martín, P., and Komatsu, M. (2018). p62/SQSTM1 - steering the cell through health and disease. J. Cell Sci. *131*, jcs222836.

Sawa-Makarska, J., Abert, C., Romanov, J., Zens, B., Ibiricu, I., and Martens, S. (2014). Cargo binding to Atg19 unmasks additional Atg8 binding sites to mediate membrane-cargo apposition during selective autophagy. Nat. Cell Biol. *16*, 425–433.

Schindelin, H., Marahiel, M.A., and Heinemann, U. (1993). Universal nucleic acid-binding domain revealed by crystal structure of the B. subtilis major cold-shock protein. Nature *364*, 164–168.

Schneider, C.A., Rasband, W.S., and Eliceiri, K.W. (2012). NIH Image to ImageJ: 25 years of image analysis. Nat. Methods *9*, 671–675.

Seibenhener, M.L., Babu, J.R., Geetha, T., Wong, H.C., Krishna, N.R., and Wooten, M.W. (2004). Sequestosome 1/p62 is a polyubiquitin chain binding protein involved in ubiquitin proteasome degradation. Mol. Cell. Biol. *24*, 8055–8068.

Sheldrick, G.M. (2010). Experimental phasing with SHELXC/D/E: combining chain tracing with density modification. Acta Crystallogr. D Biol. Crystallogr. *66*, 479–485.

Shintani, T., Huang, W.-P., Stromhaug, P.E., and Klionsky, D.J. (2002). Mechanism of cargo selection in the cytoplasm to vacuole targeting pathway. Dev. Cell *3*, 825–837.

Sikorski, R.S., and Hieter, P. (1989). A system of shuttle vectors and yeast host strains designed for efficient manipulation of DNA in Saccaromyces cerevisiae. Genetics *122*, 19–27.

Smith, M.D., Harley, M.E., Kemp, A.J., Wills, J., Lee, M., Arends, M., von Kriegsheim, A., Behrends, C., and Wilkinson, S. (2018). CCPG1 is a non-canonical autophagy cargo receptor essential for ER-phagy and pancreatic ER proteostasis. Dev. Cell *44*, 217–232.e11.

Steinkellner, G., Rader, R., Thallinger, G.G., Kratky, C., and Gruber, K. (2009). VASCo: computation and visualization of annotated protein surface contacts. BMC Bioinformatics *10*, 32.

Stolz, A., Ernst, A., and Dikic, I. (2014). Cargo recognition and trafficking in selective autophagy. Nat. Cell Biol. *16*, 495–501.

Sun, D., Wu, R., Zheng, J., Li, P., and Yu, L. (2018). Polyubiquitin chain-induced p62 phase separation drives autophagic cargo segregation. Cell Res. *28*, 405–415.

Tanaka, C., Tan, L.-J., Mochida, K., Kirisako, H., Koizumi, M., Asai, E., Sakoh-Nakatogawa, M., Ohsumi, Y., and Nakatogawa, H. (2014). Hrr25 triggers selective autophagy-related pathways by phosphorylating receptor proteins. J. Cell Biol. *207*, 91–105.

Tanji, K., Miki, Y., Ozaki, T., Maruyama, A., Yoshida, H., Mimura, J., Matsumiya, T., Mori, F., Imaizumi, T., Itoh, K., et al. (2014). Phosphorylation of serine 349 of p62 in Alzheimer's disease brain. Acta Neuropathol. Commun. *2*, 50.

Torggler, R., Papinski, D., Brach, T., Bas, L., Schuschnig, M., Pfaffenwimmer, T., Rohringer, S., Matzhold, T., Schweida, D., Brezovich, A., and Kraft, C. (2016). Two independent pathways within selective autophagy converge to activate Atg1 kinase at the vacuole. Mol. Cell *64*, 221–235.

Tyanova, S., Temu, T., and Cox, J. (2016). The MaxQuant computational platform for mass spectrometry-based shotgun proteomics. Nat. Protoc. *11*, 2301–2319.

Wurzer, B., Zaffagnini, G., Fracchiolla, D., Turco, E., Abert, C., Romanov, J., and Martens, S. (2015). Oligomerization of p62 allows for selection of ubiquitinated cargo and isolation membrane during selective autophagy. eLife *4*, e08941.

Zaffagnini, G., and Martens, S. (2016). Mechanisms of selective autophagy. J. Mol. Biol. *428* (9 Pt A), 1714–1724.

Zaffagnini, G., Savova, A., Danieli, A., Romanov, J., Tremel, S., Ebner, M., Peterbauer, T., Sztacho, M., Trapannone, R., Tarafder, A.K., et al. (2018). p62 filaments capture and present ubiquitinated cargos for autophagy. EMBO J. *37*, e98308.

STAR★METHODS

KEY RESOURCES TABLE

REAGENT or RESOURCE	SOURCE	IDENTIFIER
Antibodies		
Mouse anti-p62	BD Bioscience	Cat#610832; RRID: AB_398152
Mouse anti-GST	Sigma	Cat#SAB4200237; RRID: AB259845
Rabbit anti-FIP200 (D10D11)	Cell Signaling Technology	Cat#12436
Rabbit anti-FIP200	Atlas antibodies	Cat#HPA053049; RRID: AB_2682025
Mouse anti-FIP200 (6B2)	MFPL antibody facility	N/A
Rabbit anti-Stx17	Sigma	Cat#HPA001204; RRID: AB_1080118
Mouse anti-LC3B (clone 2G6)	nanoTools	Cat#0260-100
Rabbit anti-GABARAP	Cell Signaling Technology	Cat#13733
Mouse anti-GFP	Roche	Cat#11814460001; RRID: AB_390913
Mouse anti-myc (clone 4A6)	Millipore	Cat#05-724; RRID: AB_310809
Rabbit anti-Atg19	Eurogentec	N/A
Rabbit anti-Ape1		Gift from Kraft lab (Institute for Biochem. And Mol. Biol., Freiburg, Germany)
Mouse anti-Atg11 clone 6FG-G4	MFPL antibody facility	N/A
Rabbit anti-phospho-p62 S349	Cell Signaling Technology	Cat#95697
Mouse anti-GAPDH	Sigma	Cat#G8795; RRID: AB_1078991
Rabbit anti-p62	MBL	Cat#PM045; RRID: AB_1279301
Mouse anti-ubiquitin FK2	Enzo Life Science	Cat#BML-PW8810; RRID: AB_10541840
Rabbit anti-ULK1 H-240	Santa Cruz Biotech.	Cat#sc-33182; RRID: AB_2214706
Rabbit anti-ATG16L1 D6D5	Cell Signaling Technology	Cat#8089; RRID: AB_10950320
Goat polyclonal anti-mouse HRP	Jackson Immunoresearch	Cat#115-035-003; RRID: AB_10015289
Goat polyclonal anti-rabbit HRP	Jackson Immunoresearch	Cat#111-035-003; RRID: AB_2313567
Goat anti-rabbit Alexa Fluor 488	Invitrogen	Cat#A11008; RRID: AB_143165
Goat anti-mouse Alexa Fluor 488	Invitrogen	Cat#A11001; RRID: AB_2534069
Goat anti-mouse Alexa Fluor 647	Jackson Immunoresearch	Cat#115-605-146; RRID: AB_2338912
Goat anti-rabbit Alexa Fluor 647	Jakcson Immunoresearch	Cat#111-605-144; RRID: AB_2338078
Bacterial and Virus Strains		
E. coli Rosetta (DE3) pLys	Novagen	Cat#70956
E. coli Rosetta2 (DE3)	Novagen	Cat#71397
Chemicals, Peptides, and Recombinant Proteins		
cOmplete EDTA-free protease inhibitor cocktail	Roche	Cat#11836170001
Proteinase K from *Engyodontium album*	Sigma	Cat#P2308
Lysyl Endopeptidase C	Wako	Cat#125-02543
Bradford protein assay	Bio-Rad	Cat#5000006
DC protein assay kitII	Bio-Rad	Cat#5000112
GSTrap HP column	GE Healthcare	Cat#17528201
HisTrap HP column	GE Healthcare	Cat#17524801
Glutathione Sepharose 4B beads	GE Healthcare	Cat#17075601
GFP-trap_A beads	Chromotek	Cat#gta-20
RFP-trap_A beads	Chromotek	Cat#rta-20
Strep Tactin Sepharose HP	GE Healthcare	Cat#28935599
Dynabeads M-270 Epoxy	ThermoFisher	Cat#14301
Pierce anti-HA magnetic beads	ThermoFisher	Cat#88836

(*Continued on next page*)

Continued

REAGENT or RESOURCE	SOURCE	IDENTIFIER
Puromycin	ThermoFisher	Cat#A1113802
MG132 (Z-Leu-Leu-Leu-CHO)	Boston Biochem	Cat#I-130
BafilomycinA1	Santa Cruz Biotech.	Cat#sc-201550
Wortmannin	Sigma	Cat#W1628
Lipofectamine RNAiMAX Transfection Reagent	ThermoFisher	Cat#13778030
Fugene HD Transfection Reagent	Promega	Cat#E2311
DAPI-Fluoromount-G	SouthernBiotech	Cat#0100-20
Pefabloc SC-Protease inhibitor	Carl Roth	Cat#A154.3
Deposited Data		
FIP200 CTR structure	PDB	PDB: 6GMA
FIP200 Claw domain structure	PDB	PDB: 6DCE
Original data for this study	Mendeley data	https://doi.org/10.17632/jdv2yxymc5.1
Experimental Models: Cell Lines		
HeLa cells (CCL-2)	ATCC	ATCC CCL-2
HAP1 cells	Horizon Genomics	Cat#C631
HAP1 STG-p62 wt clone 3D	Zaffagnini et al., 2018	$SM_{CL}33$
HAP1 STG-p62 LIRmut clone 1B	Zaffagnini et al., 2018	$SM_{CL}50$
HAP1 FIP200-STG clone 2	This study	$SM_{CL}44$
HeLa FIP200ΔClaw clone A3	This study	SM_{CL} 62
Experimental Models: Organisms/Strains		
S. cerevisiae: background strain S288c BY4741	Euroscarf	Y00000
S. cerevisiae: S288c BY4741 - atg11Δ (atg11::kan)	Pfaffenwimmer et al., 2014	Gift from Kraft lab (Institute for Biochem. And Mol. Biol., Freiburg, Germany)
Oligonucleotides		
Non-tergeting siRNA pool ON-target Plus	Dharmacon	D-001810-10-50
FIP200 siRNA ON-target Plus	Dharmacon	J-021117-05-0010
FIP200-STG sgRNAF1: caccgAAATCTGTTTTGTGCCTAAG	This study	SMP1393
FIP200-STG sgRNAR1: aaacCTTAGGCACAAAACAGATTTc	This study	SMP1394
FIP200-STG sgRNAF2: caccgACAGAGTGAAAGCCGTATCA	This study	SMP1395
FIP200-STG sgRNAR2: aaacTGATACGGCTTTCACTCTGTc	This study	SMP1396
FIP200ΔClaw sgRNAB1: caccGCATGTCTTCAGTATCTTCA	This study	SMP 2146
FIP200ΔClaw sgRNAB2: CGTACAGAAGTCATAGAAGTcaaa	This study	SMP 2147
Recombinant DNA		
pGEX-GST-FIP200 CTR aa 1429-1594	This study	SMC565
pET-mCherry-p62 wt	Wurzer et al., 2015	SMC391
pET-mCherry-p62 LIRmut	Wurzer et al., 2015	SMC542
pGEX-2x ubiquitin	Wurzer et al., 2015	Gift from Ikeda lab (IMBA, Vienna, Austria)
pGEX-4x ubiquitin	Wurzer et al., 2015	Gift from Ikeda lab (IMBA, Vienna, Austria)
pET-His-TEV-GFP-FIP200 CTR aa 1429-1594	This study	SMC707
pET-His-TEV-GFP-FIP200 CTR aa 1458-1594	This study	SMC750
pET- His-TEV-FIP200 CTR aa 1458-1594	This study	SMC752
pET-His-TEV-FIP200 Claw aa 1494-1594	This study	N/A
pGEX-p62 LIR	This study	SMC785
pGEX-p62 aa334-356	This study	SMC786

(*Continued on next page*)

Continued

REAGENT or RESOURCE	SOURCE	IDENTIFIER
pGEX-p62 aa 334-373	This study	SMC787
pGEX-p62 FIR	This study	SMC788
pGEX-p62 FIRmut	This study	SMC818
pGEX-GST-Atg19-C-ter	Sawa-Makarska et al., 2014	SMC295
pGEX-GST-Atg19-C-ter-mut	Abert et al., 2016	SMC805
pGEX-GST-Atg19 C-ter 3D	This study	SMC494
pGEX-GST-Atg19 C-ter 3DLL	This study	SMC879
pGEX-GST-Atg19 C-ter 3DFF	This study	SMC694
pGEX-GST-Atg19 C-ter 3DW	This study	SMC892
pGEX-GST-Atg19 C-ter 3DFFLL	This study	SMC878
pGEX-GST-Atg19 C-ter 3DLLW	This study	SMC877
pGEX-GST-Atg19 C-ter 3DFFLLW	This study	SMC876
pET-His-TEV-GFP-Atg11 CTD	This study	SMC822
pET-His-TEV-EGFP	Zaffagnini et al., 2018	SMC559
pET-His-TEV-GFP-LC3B	Wurzer et al., 2015	SMC459
pET-His-TEV-LC3B	This study	SMC893
pGEX-p62 FIR 1P	This study	SMC962
pGEX-p62 FIR 3P	This study	SMC960
pGEX-p62 FIR 4P	This study	SMC996
pET-His-TEV-mCherry-p62 4P	This study	SMC1035
pET-His-TEV-GFP-FIP200 CTR N1517A	This study	SMC1004
pET-His-TEV-GFP-FIP200 CTR N1517F	This study	SMC1003
pET-His-TEV-GFP-FIP200 CTR S1532A	This study	SMC1007
pET-His-TEV-GFP-FIP200 CTR L1535A	This study	SMC1006
pET-His-TEV-GFP-FIP200 CTR L1535F	This study	SMC1005
pET-His-TEV-GFP-FIP200 CTR R1573D	This study	SMC957
pET-His-TEV-GFP-FIP200 CTR F1574A	This study	SMC1001
pET-His-TEV-GFP-FIP200 CTR F1574W	This study	SMC1000
pET-His-TEV-GFP-FIP200 CTR R1584A	This study	SMC1008
pME18s-HA-hFIP200	Addgene plasmid	Cat#24303
pME18s-HA-hFIP200 R1573D	This study	SMC1123
pME18s-HA-hFIP200 F1574W	This study	SMC1124
pME18s-HA-hFIP200 aa1-1457	This study	SMC1128
pSpCas9n(BB)-2A-Puro (PX462)	Addgene plasmid	Cat#62987
pSpCas9(BB)-2A-GFP (PX458)	Addgene plasmid	Cat#48138
pUC19	Addgene plasmid	Cat#50005
pRS315	Sikorski and Hieter, 1989	Gift from Kraft lab (Institute for Biochem. And Mol. Biol., Freiburg, Germany)
pRS315-Atg11 wt	This study	SMC991
pRS315-Atg11 ΔCTR	This study	SMC1033
pRS315-9xmyc-Atg11	This study	Gift from Kraft lab (Institute for Biochem. And Mol. Biol., Freiburg, Germany)
pRS315-9xmyc-Atg11 ΔCTR	This study	SMC1069
Software and Algorithms		
ImageJ 1.x	Schneider et al., 2012	https://imagej.net/ImageJ1
GraphPad Prism 7.05		https://www.graphpad.com

(*Continued on next page*)

Continued

REAGENT or RESOURCE	SOURCE	IDENTIFIER
XDS	Kabsch, 2010	http://xds.mpimf-heidelberg.mpg.de/
XDSAPP	Krug et al., 2012	https://www.helmholtz-berlin.de/forschung/oe/np/gmx/xdsapp/index_en.html
Phenix	Adams et al., 2010	http://www.phenix-online.org/
Coot	Emsley et al., 2010	https://www2.mrc-lmb.cam.ac.uk/personal/pemsley/coot/
PyMol 2.0	DeLano, 2014	https://pymol.org/2/
VASCo	Steinkellner et al., 2009	http://genome.tugraz.at/VASCo/
PDBeFold	Krissinel and Henrick, 2007	http://www.ebi.ac.uk/msd-srv/ssm/
ARP/ wARP	Langer et al., 2008	http://www.embl-hamburg.de/ARP/

CONTACT FOR REAGENT AND RESOURCE SHARING

Further information and requests for resources and reagents should be directed to and will be fulfilled by the Lead Contact, Sascha Martens (sascha.martens@univie.ac.at).

EXPERIMENTAL MODEL AND SUBJECT DETAILS

Cell lines and cell culture

All cell lines were cultivated at 37°C in humidified 5% CO_2 atmosphere. HeLa cells CCL-2 were purchased from ATCC and grown in Dulbecco Modified Eagle Medium (DMEM, GIBCO, Thermo Fisher) supplemented with 10% (v/v) Fetal Bovine Serum (Thermo Fisher) and Penicillin-Streptomycin (5000 U/ml – GIBCO, Thermo Fisher).

HAP1 cells were purchased from Horizon Discovery and cultivated in Iscove's Modified Dulbecco's Medium (IMDM - GIBCO, Thermo Fisher Scientific), supplemented with 10% (v/v) Fetal Bovine Serum (Thermo Fisher Scientific), Penicillin-Streptomycin (5,000 U/mL) (GIBCO, Thermo Fisher Scientific).

Generation of endogenously tagged cell lines

HAP1 STG-p62 cells (wt and LIRmut) were generated as described previously (Zaffagnini et al., 2018). Briefly, sgRNAs were cloned individually into pSPCas9n(BB)-2A-Puro (PX462-Addgene). The repair template was cloned into pUC19 and cells were co-transfected with the sgRNA bearing vectors and repair template containing pUC19. After 72 h of Puromycin selection cells were sorted by FACS for green fluorescence into 96-well plates. Clones were screened for the insertion of the tag by PCR and validated by western blotting.

HAP1 FIP200-STG cells were generated by cloning sgRNAs (designed by the help of CRISPR design tool, https://zlab.bio/guide-design-resources) into the pSpCas9n(BB)-2A-Puro (PX462) vector (Addgene).

HAP1 cells were co-transfected with two sgRNA bearing vectors (PX462) and a pUC19 vector (Addgene) containing the repair template for 48 hr. Puromycin (Thermo Fisher) selection was applied for 3 days and then single GFP expressing cells were FACS sorted into 96-well plates. Integration of the insert into HAP1 genome was confirmed by sequencing. As test of autophagy flux in this cell line compared to HAP1 wt, LC3B lipidation and p62 degradation were assessed by WB after 2 h starvation in Earlés Balanced Salt Solution (EBSS) medium (Sigma) +/− Bafilomycin (Santa Cruz Biotechnology) treatment (Figure S6A). Membranes were probed with the following antibodies: rabbit anti-FIP200 (1:1000 - Cell Signaling), mouse anti-p62 (1:3000 - BD Bioscience), mouse anti-LC3B (1:500 - nanoTools), mouse anti-GAPDH (1:25000 - Sigma).

Generation of HeLa FIP200ΔClaw cell line

To generate HeLa FIP200ΔClaw cells, sgRNAs targeting the region around R1591 of FIP200 were cloned into pSpCas9(BB)-2A-GFP (PX458) vector (Addgene). HeLa cells were transfected with the vector containing the sgRNAs and after 24 h, GFP-Cas9 expressing cells were sorted by FACS into 96-well plates. Deletion of the Claw domain in the selected clones was confirmed by western blotting and sequencing of the genomic region.

S. cerevisiae *strains*

S. cerevisiae strain BY4741 was purchased from EUROSCARF. Cells were grown in nutrient-rich (YPD) or starvation (SD-N) medium, as specified in the method details at 30°C. The Atg11Δ strain was a gift from the Kraft lab (Pfaffenwimmer et al., 2014).

E. coli *strains*

E. coli strains used to express recombinant proteins were purchased from Novagen and grown as described in the method details.

METHOD DETAILS

Protein expression and purification

mCherry-p62 wt and the LIR mutant, GST-2x ubiquitin, GST-4x ubiquitin, GFP-LC3B, GST-Atg19 C terminus and Atg8 were expressed and purified as previously described (Sawa-Makarska et al., 2014; Wurzer et al., 2015) and according to the expression and purification protocols described below for the proteins obtained for this study. All the GST-tagged constructs were sub-cloned into the pGEX 4T1 vector. The GFP-tagged constructs of FIP200 were obtained by first cloning the GFP sequence into pETDuet 1 vector downstream of the 6xHis tag and then inserting FIP200 sequence in frame after the GFP coding sequence. A Tobacco Etch Virus (TEV) protease cleavage site was inserted between the 6xHis tag and the GFP tag to allow the removal of the 6xHis tag from the purified protein. All the point mutations were obtained by round the horn or quickchange site directed mutagenesis. Proteins were expressed in *E. coli* Rosetta (DE3) pLysS cells. Cells were grown at 37°C to an OD_{600} of 0.6, induced with 0.1 mM IPTG and grown for additional 16 h at 18°C. Cells were harvested by centrifugation and the cell pellet was resuspended in lysis buffer (50 mM HEPES pH 7.5, 300 mM NaCl, 2 mM $MgCl_2$, 2 mM β-mercaptoethanol, 1mM Pefabloc, DNase I (Sigma)). For 6xHis tagged protein, the lysis buffer was supplemented with 10 mM imidazole. Cells were lysed by one freeze and thaw cycle followed by sonication and the lysate was cleared by ultracentrifugation at 40,000xg for 40 min at 4°C. GST fused proteins were purified using a GSTrap HP 5 mL column (GE Healthcare) equilibrated with 50 mM HEPES pH 7,5, 300 mM NaCl, 1 mM DTT and eluted with 20 mM reduced L-glutathione (Sigma) in the same buffer at pH 8.0. 6xHis tagged proteins were purified on a HisTrap HP 5 mL column (GE-Healthcare) equilibrated with 50 mM HEPES pH 7.5, 300 mM NaCl, 10 mM imidazole, 2 mM β-mercaptoethanol. Proteins were eluted by a stepwise imidazole gradient. Protein containing fractions were pooled and the 6xHis tag was cleaved with TEV protease O/N at 4°C. After affinity purification, all proteins were further purified by size exclusion chromatography on a Superdex S200 column (GE-Healthcare) equilibrated with 25 mM HEPES pH 7.5, 150 mM NaCl, 1 mM DTT.

Expression and purification of GST-TEV-FIP200-MBP followed by FITC-labeling

FIP200 was sub-cloned into the pCAG vector with a GST tag nucleotide sequence at the 5′ and MBP tag at the 3′ of the ORF. A TEV cleavage site was inserted between GST and FIP200. The GST-TEV-FIP200-MBP was expressed in HEK293-GnT1 suspension cells. Cells were infected at the concentration of 2-2.5 million/ml and harvested after 48-60 h. The harvested cells were pelleted at 2,500 rpm for 20 min at 4°C, and washed with PBS once. The pellets were then lysed in 50 mM Tris-HCl pH 7.4, 200 mM NaCl, 2 mM $MgCl_2$, 1 mM TCEP, 1% Triton X-100, 10% Glycerol and protease inhibitors (Roche) before being cleared at 15,000 rpm for 30 min at 4°C. Additional 300 mM NaCl was added into the supernatant. The GST-TEV-FIP200-MBP protein was purified using Glutathione Sepharose 4B (GE Healthcare) and eluted with 50 mM reduced L-glutathione (Sigma) in the lysis buffer. The obtained protein was either used for experiments or further processed as follows. The GST tag was cleaved with TEV protease over night at 4°C to allow the exposure of an N-terminal glycine residue for subsequent labeling with Δ59SortaseA. The FIP200-MBP protein was then purified using Amylose resin (New England Biolabs) and eluted with 20mM HEPES pH 8.0, 200 mM NaCl, 1 mM TCEP and 50 mM Maltose and snap frozen in liquid nitrogen.

FITC labeling was conducted mixing FITC-conjugated peptide (FITC-LPETGG, from GenScript) with Gly-FIP200-MBP protein at a peptide/protein ratio of 10:1 in the presence of Δ59SortaseA enzyme in a buffer containing 50 mM Tris-HCl pH 7.5, 150 mM NaCl and 10 mM $CaCl_2$. Labeling was conducted for 2 hr at RT in the dark. The labeling was confirmed by gel electrophoresis of the samples collected before (t_0) and after the addition of the enzyme to the reaction. Fluorescence was detected using ChemiDoc instrument (BioRad) equipped with Fluorescein Filter prior to Coomassie staining. Labeling reaction mix was further processed by gel filtration on a Superose6_10/300 column (GE-Healthcare) pre-equilibrated in buffer containing 20 mM HEPES pH 7.5, 200 mM NaCl and 1 mM DTT. Fractions eluting at the expected elution volume, positive for the labeling and containing the protein of interest were pooled, concentrated through a 100 kDa cut-off Amicon filter and used immediately for the assay without further freezing.

Microscopy-based protein-protein interaction assays

In this assay beads bound bait proteins are incubated with a dilution of a fluorescently labeled prey protein. Recruitment of the prey will result in accumulation of fluorescence around the beads which can be visualized by microscopy. The advantage of this assay over a classic pull down is that proteins can be visualized at the equilibrium, since the prey protein is not washed away after the incubation with the bait, allowing the detection of interactions with high off rates. The fluorescent signal accumulated on the beads is proportional to the amount of protein bound to the bait and can be measured in ImageJ.

In details, glutathione Sepharose 4B beads (GE Healthcare, average diameter 90 μm) were incubated for 30 min at 4°C (16 rpm horizontal rotation) with GST-tagged bait proteins (4 mg/mL for GST-p62 mutants and GST-FIP200 CTR, 0,6 mg/ml for GST-FIP200 full-length, 35 μM for GST-Atg19 mutants). The beads were washed 2 times in 10x beads volume with washing buffer (25 mM HEPES pH7.5, 150 mM NaCl, 1 mM DTT). The buffer was removed and the beads were resuspended 1:1 in washing buffer. 10 μL of a 2-5 μM dilution of fluorescently labeled binding partners were added to the beads suspension and incubated for 30 min to 1 h at room temperature or at 4°C before imaging with a Zeiss LSM700 confocal microscope or a Visitron spinning disk microscope (Figures 1E, 2B, and 2C) with a 20X magnification. After imaging, the samples were collected and analyzed by SDS-PAGE followed by Coomassie or silver staining to visualize the amount of bait protein bound to the beads.

For the FIP200-LC3B competition assay, RFP-trap_A beads (Chromotek, average diameter 90 μm) were incubated for 30 min at 4°C (10 rpm horizontal rotation) with 4 mg/mL mCherry-p62 FIR 4P or mCherry. The beads were washed 3 times in 20x beads volume

of 25mM HEPES pH7.5, 500 mM NaCl, 1 mM DTT. The buffer was removed and the beads were resuspended 1:1 in 25 mM HEPES pH7.5, 150 mM NaCl, 1 mM DTT, 0.1% Triton-X. 0.8μL of the beads suspension were added to 8 μL of a 2 μM solution of GFP-FIP200 CTR and incubated for 30 min at room temperature before addition of 5 μL LC3B at increasing concentrations. The beads were imaged with a Visitron spinning disk microscope with 20X magnification after 20 min incubation at room temperature.

p62 pull-down from HeLa and HAP1 cell lysates

HeLa or HAP1 cells were seeded into 4 × 10 cm dishes and grown until confluence. Cells were harvested with trypsin and washed with PBS. The cell pellet was resuspended in 100 μl lysis buffer (20 mM HEPES pH 7.5, 250 mM Sorbitol, 0.5 mM EGTA, 5 mM Mg-Acetate, 0.3 mM DTT, cOmplete EDTA-free protease inhibitor cocktail (Roche)) and cells were lysed by one freeze and thaw cycle. After 10 min centrifugation at 1,000xg protein concentration in the supernatant (lysate) was measured by Bradford protein assay (Bio-Rad). 10 μl of Glutathione Sepharose 4B or GFP-Trap_A beads (GE-Healthcare and Chromotek, respectively) were incubated with 4 mg/ml of bait protein (GST-FIP200 CTR or GFP-FIP200 CTR wt/mut) for 30 min at 4°C. Beads were washed 2 times with 10 x beads volume in 25 mM HEPES pH 7.5, 150 mM NaCl, 1 mM DTT (wash buffer) and resuspended in 10 μl wash buffer. 200 μg (300 μg for HAP1) of cell lysate were added to the beads and incubated for 1 h at 4°C. Beads were washed 3 times in 10x beads volume of wash buffer and resuspended in 10 μl wash buffer. Beads bound protein were eluted by boiling the beads for 5 min at 98°C in Laemmli buffer. 5 μl of each sample were analyzed by western blotting with mouse anti-p62 (1:3000, BD Bioscience). Other 5 μl of the samples were analyzed by SDS-PAGE followed by Coomassie staining (Figure 6D) or western blot with mouse anti-GST (1:1000, Sigma; Figure 1B) to visualize the bait protein input (For the pull-down with HAP1 cells lysates in Figure 6F, the entire eluate was analyzed by western blot and Ponceau staining of the membrane was used to visualize the bait protein input).

Identification of phosphorylated residues by mass spectrometry

HAP1 cells were harvested with trypsin from 3 confluent 15 cm dishes. Cell pellet was washed 3 times in ice-cold PBS, resuspended in lysis buffer (50 mM Tris pH 7.5, 150 mM NaCl, 1 mM DTT, 1 mM NaF, 20 mM beta-glycerophosphate, 1 mM Na-orthovanadate, supplemented with cOmplete EDTA-free protease inhibitors cocktail (Roche)) and frozen in liquid nitrogen to disrupt the membranes. Lysates were thawed, spun at 500 g for 10 min at 4°C and protein concentration was measured by Bradford protein assay (Bio-Rad). For pull-downs, 3 mg of lysate were incubated with 25 μL of GFP-Trap_A beads (Chromotek) for 90 min at 4°C under gentle rotation. Afterward, the beads were washed four times with lysis buffer, transferred to new tubes and resuspended in 30 μL of 2 M urea in 50 mM ammonium bicarbonate (ABC). Disulfide bonds were reduced with 10 mM dithiothreitol for 30 min at room temperature before adding 25 mM iodoacetamide and incubating for another 30 min at room temperature in the dark. Remaining iodoacetamide was quenched by adding 5 mM DTT and the proteins were digested with 150 ng Lysyl Endopeptidase C (Wako) at room temperature overnight. The next day the supernatant was transferred to a new tube, the beads were washed with another 30 μL of 2 M urea in 50 mM ABC and the wash was combined with the supernatant. After diluting to 1 M urea with 50 mM ABC 150 ng trypsin was added and sample was digested for 6 h at 37°C in the dark. The digestion was stopped by addition of 1% trifluoroacetic acid (TFA), and the peptides were desalted using C18 Stagetips (Rappsilber et al., 2007). Peptides were separated on an Ultimate 3000 RSLC nano-flow chromatography system (Thermo Fisher), using a pre-column for sample loading (Acclaim PepMap C18, 2 cm × 0.1 mm, 5 μm, Thermo Fisher), and a C18 analytical column (Acclaim PepMap C18, 50 cm × 0.75 mm, 2 μm, Thermo Fisher), applying a segmented linear gradient from 2% to 80% solvent B (80% acetonitrile, 0.1% formic acid; solvent A 0.1% formic acid) at a flow rate of 230 nL/min over 120 min. Eluting peptides were analyzed on a Q Exactive HF Orbitrap mass spectrometer (Thermo Fisher), which was coupled to the column with a nano-spray Flex ion-source (Thermo Fisher) using coated emitter tips (New Objective).

Data-dependent mass spectrometry analysis

The mass spectrometer was operated in data-dependent acquisition mode (DDA), survey scans were obtained in a mass range of 375-1500 m/z with lock mass activated, at a resolution of 60k at 200 m/z and an AGC target value of 3E6. The 8 most intense ions were selected with an isolation width of 1.6 m/z, fragmented in the HCD cell at 27% collision energy and the spectra recorded for max. 250 ms at a target value of 1E5 and a resolution of 30k. Peptides with a charge of +1 or > +6 were excluded from fragmentation, the peptide match feature was set to preferred, the exclude isotope feature was enabled, and selected precursors were dynamically excluded from repeated sampling for 20 s.

Raw data were processed using the MaxQuant software package (version 1.6.0.16) (Tyanova et al., 2016) and the Uniprot human reference proteome (https://www.uniprot.org/) as well as a database of most common contaminants. The search was performed with full trypsin specificity and a maximum of two missed cleavages at a protein and peptide spectrum match false discovery rate of 1%. Carbamidomethylation of cysteine residues were set as fixed, oxidation of methionine, phosphorylation of serine, threonine, and tyrosine, and N-terminal acetylation as variable modifications. For label-free quantification the "match between runs" feature and the LFQ function were activated - all other parameters were left at default. To further validate the phosphosites found in the FIR, we set-up a targeted method.

Targeted mass spectrometry analysis

Parallel reaction monitoring (PRM) assays were generated based on the DDA results. We focused on the p62 peptide at position 345-378 which carried most of the potential phosphorylation sites in the FIR, and recorded spectra for the +3 precursor mass with up to four phosphorylations over the whole gradient. For PRM data acquisition, we operated the same instrument type as for shotgun MS, applying a 120 min gradient for chromatographic separation. The segmented gradient was adapted to start at

20% solvent B to account for the late elution of the peptide and of potential multiply phosphorylated forms. The following MS parameters were used: survey scan with 30k resolution, AGC 1E6, 30 ms IT, over a range of 600 to 1400 m/z, PRM scan with 60 k resolution, AGC 1E5, 750 ms IT, isolation window of 1.2 m/z with 0.5 m/z offset, and NCE of 27%.

Data analysis and manual validation were performed in Skyline (MacLean et al., 2010). To generate a spectral library, data were searched in Mascot 2.2.07 at 10 ppm peptide and 20 mmu fragment mass tolerance, and the same modifications as above. For manual validation, extracted ion chromatograms (XIC) for all singly phosphorylated versions were generated in Skyline and in a second step also for doubly phosphorylated versions using combinations of the most prominent singly phosphorylated sites. No signals for 3x or 4x phosphorylated peptides could be detected. Transitions indicative for a specific site were manually validated in terms of mass accuracy (+/− 2 ppm) and consistent elution patterns - the XICs are presented in the Figure S2. Since all singly phosphorylated variants displayed the same elution behavior the PRM actually generated mixed spectra which contain signatures of several phosphopeptides and which made an unambiguous designation for some of the potential sites impossible. Nevertheless, in combination with the data of the doubly phosphorylated peptide a clear assignment for the most prominent sites could be achieved.

Surface plasmon resonance spectroscopy

All SPR experiments were performed on a Biacore T200 instrument (GE Healthcare) at 25°C in 50 mM Tris HCl pH 7.5, 150 mM NaCl. GST, GST-p62 FIR wt, GST-p62 FIR LIRmut, and GST-p62 FIR 4P were diluted in 10 mM acetate buffer pH 3.8, 10 mM NaCl and immobilized on a CM 5 Series S sensor chip (GE Healthcare) using the Amine Coupling Kit (GE Healthcare). High density immobilization was achieved by coupling the proteins to a theoretical R_{max} of approximately 6000 RU. After three conditioning cycles with 30 μM FIP200 CTR and regeneration in 10 mM glycine pH 2.1, FIP200 CTR was passed over the four flow channels at a flow rate of 30 μl/min. Single-cycle data were collected as two technical replicates for three independent experiments using a three-fold dilution series of FIP200 CTR (0.4 μM – 30 μM). Association of FIP200 CTR was monitored for 180 s, followed by dissociation in buffer for 70 s. After each cycle, remaining FIP200 CTR was stripped off the surface with two 60 s injections of 10 mM glycine pH 2.1. A new chip was used for each of the independent experiments. Evaluation was performed using the Biacore T200 Evaluation Software 3.0 (GE Healthcare). Data were double referenced by subtracting the GST and buffer control signals. Data points at equilibrium were fitted globally with a one site binding model (Response = R_{max} x [protein]/(K_D + [protein]), where R_{max} is the fitted maximal binding capacity and K_D the apparent dissociation constant, using GraphPad Prism (GraphPad Software, Inc.).

Protease protection assay

HeLa cells were seeded into 6-well plates (400,000 cells per well) the day before treatment. 5 μM Puromycin (Thermo Fisher), 400 nM Bafilomycin (Santa Cruz Biotechnology), 1 μM Wortmannin (Sigma) or a combination of these were added to the cells for 2 h in fresh Earle's Balanced Salt Solution (Sigma) for starvation experiments or DMEM-10% FBS for all of the other samples. Cells were washed twice in ice-cold PBS, harvested and homogenized in 20 mM HEPES (pH 7.5), 220 mM mannitol, 70 mM sucrose, 1 mM EDTA by passing 4 times through a 26-gauge syringe needle and then by a glass homogenizer (30 strokes). Samples were then spun for 10 min at 500xg at 4°C to remove nuclei and unbroken cells. Each supernatant was split in three different tubes: one was left untreated, the other two were incubated with either 100 μg/ml proteinase K (Sigma) or a combination of proteinase K and 0.5% Triton X-100 in a final volume of 1 mL for 30 min on ice. Proteolysis was terminated by the addition of 1 mM PMSF for 10 min on ice. Finally, samples were precipitated by adding 250 μL trichloroacetic acid for 10 min on ice and washed twice with 200 μL acetone. Protein pellets were resuspended in Laemmli buffer and heated at 95°C for 5 min prior to SDS-PAGE.

For western blot, the following antibodies were used: rabbit anti-FIP200 (1:1000, Cell Signaling), rabbit anti-STX-17 (1:1000, Sigma), mouse anti-p62 (1:1000, BD Biosciences) and mouse anti-LC3B (1:500 nanoTools).

Crystallization and structure determination

N-terminally His_6-tagged FIP200 CTR was expressed in *E. coli* Rosetta2 (DE3) cells (Novagen) at 18°C overnight after induction with 0.2 mM isopropyl-β-d-thiogalactoside. Cells were sedimented by centrifugation for 20 min at 5000 *g* and 4°C, resuspended in 30 mL lysis buffer (50 mM HEPES pH 7.5, 300 mM NaCl, 10 mM imidazole, 1 mM $MgCl_2$) and mechanically disrupted by a microfluidizer (Microfluidics) in the presence of 10 μg ml^{-1} DNase (Roche) and 1 mM protease inhibitor 4-(2-aminoethyl)benzenesulfonyl fluoride hydrochloride (AEBSF). Cell debris was removed by centrifugation at 140,000 *g* for 45 min before the supernatant was applied on Ni-NTA resin (GE Healthcare) previously equilibrated with lysis buffer. After washing the column with 50 mM HEPES pH 7.5, 500 mM NaCl, 75 mM imidazole, 10 mM $MgCl_2$, 10 mM KCl, 1 mM ATP, the protein was eluted in 50 mM HEPES pH 7.5, 300 mM NaCl, 500 mM imidazole. For over-night dialysis into size exclusion buffer (25 mM HEPES pH 7.5, 150 mM NaCl), the protein was concentrated to 10 mL and incubated at a molar ratio of 1:15 with TEV protease. The cleaved protein was applied to the Ni-NTA column again and elution was performed stepwise with elution buffer (25 mM HEPES pH 7.5, 150 mM NaCl, (I) 100 mM imidazole (II) 150 mM imidazole (III) 200 mM imidazole). Fractions containing untagged protein were further purified by size exclusion chromatography on a Superdex S75 16/600 column (GE Healthcare). Peak fractions containing the protein of interest were pooled and concentrated to 30 mg/ml.

L-seleno-methionine-labeled protein was expressed in minimal medium supplemented with trace elements, vitamins, and amino acids (Doublie, 1997) and purified in the same way as native protein. Initial crystallization screens were performed in 96-well plates using the vapor-diffusion method. After optimization, native rod-shaped crystals grew within 24 h to 72 h at 20°C in 0.1 M MES pH 6.0,

13%–15% PEG 6000, 0.8 – 1.2 M LiCl using a protein to reservoir ratio of 1:1. Crystals of L-seleno-methionine-labeled protein grew in similar crystallization conditions (0.1 M MES pH 6.0, 8%–11% PEG 6000, 1.0 – 1.2 M LiCl, 10 mM DTT). Datasets were collected at beamline BL14.1, BESSY II, (Berlin, Germany) (Mueller et al., 2015). Diffraction data were indexed, integrated, and scaled using XDS (Kabsch, 2010) or XDSapp (Krug et al., 2012). Location of anomalous scatterers, generation of experimental phases and density modification was performed using PHENIX (Adams et al., 2010). Iterative model building and refinement were done with Coot (Emsley et al., 2010) and PHENIX, using 3 TLS (Translation-Libration-Screw-rotation) parameters per chain, employing non-crystallographic symmetry and secondary structure restraints. Figures were prepared using PyMol (DeLano, 2014). To calculate the lipophilic surface potential, the PyMol-plugin VASCo was used (Steinkellner et al., 2009). The dimer interface was analyzed with the PISA server (Krissinel and Henrick, 2007). Coordinates and diffraction data have been deposited in the Protein Data Bank (PDB) with accession code 6GMA.

For the crystallization of FIP200 Claw domain, the DNA (aa 1494-1594) was subcloned into 1B vector and expressed in *E.coli* BL21(DE3) cells. After induction with 0.5 mM isopropyl-β-d-thiogalactoside overnight at 18°C, the cells were pelleted by centrifugation at 4,000 g for 20 min. Cell pellets were lysed in 50 mM Tris-HCl pH 8.0, 500 mM NaCl, 0.5 mM TCEP, 5 mM imidazole and 1 mM phenylmethylsulphonyl fluoride by ultrasonication. The lysate was centrifugated at 15,000 g for 50 min at 4°C. The supernatant was loaded into Ni-NTA resin and washed with 20 mM imidazole and further eluted with 300 mM imidazole. The elution was incubated with tobacco etch virus protease and dialyzed into 20 mM Tris-HCl pH 8.0, 150 mM NaCl, 0.5 mM TCEP at 4°C overnight. The protein was reloaded to Ni-NTA column and the flow through was collected. The protein was further purified by a Superdex 75 (GE Healthcare) column equilibrated in 20 mM Tris-HCl pH 8.0, 150 mM NaCl, 0.5 mM TCEP. For selenomethionine labeling, the protein was expressed in M9 minimal medium and 150 mg selenomethionine were added when OD600 reached 0.9. The protein was purified as above.

The purified protein was concentrated to 3.4 mg/ml for crystallization. Crystals were grown in sitting drop at 19°C. The protein solution was mixed with an equal amount of reservoir buffer composed of 0.1 M HEPES pH 7.4, 2 M Ammonium sulfate.

Diffraction data were collected from a SeMet-substituted crystal of FIP200 Claw domain at the peak wavelength on ALS beamline 8.3.1. Diffraction data were processed using XDS (Kabsch, 2010), and initial phases were determined by single wavelength anomalous dispersion (SAD) in ShelxC/D/E (Sheldrick, 2010), which located the expected sole selenium site. An initial structure model was auto-built using ARP/wARP (Langer et al., 2008). Iterative rounds of manual model building in Coot (Emsley et al., 2010) and refinement in PHENIX (Adams et al., 2010). The coordinates and diffraction data have been deposited in PDB with accession code 6DCE.

Right-angle light scattering

100 μl of a 3 mg/ml FIP200 CTR solution was applied to a Superdex S75 10/300 size exclusion chromatography column coupled to a RALS (Right-Angle Light Scattering)-refractive index detector (Malvern). The running buffer contained 25 mM HEPES pH 7.5 and 150 mM NaCl.

p62 aggregation assay

p62 aggregation assay (Figures 5D and 5E) and quantification were performed as described previously (Zaffagnini et al., 2018). Briefly, 20 μM GST-4x ubiquitin was added to a protein mixture of 20 μM GFP-FIP200 CTR (wt or R1573D, F1574A, F1574W and R1584A mutants) and 2 μM mCherry-p62 wt. Aggregate formation was monitored over time using a Visitron inverse spinning disk microscope equipped with a Yokogawa CSU-X1 spinning disk, a standard CCD camera (CoolSNAP HQ^2), 561nm DPSS laser (100mW, AOTF-controlled) and LD Achroplan 20x/0.4 Corr objective. Images were taken every 30 s for 30 min. For the GFP-FIP200 CTR recruitment to preformed aggregates (Figure 5C), mCherry-p62 (2 μM) and GST-4x ubiquitin (10 μM) were incubated for 30 min at room temperature to allow aggregates formation. 1 μM GFP-FIP200 CTR (wt or mutants) was added and images were taken after 30 min incubation at room temperature with a Zeiss LSM 700 confocal microscope with 63x magnification.

Immunocytochemistry

For immunocytochemistry analysis, cells were grown on glass coverslips (∅ 12 mm, high precision, Marienfeld-superior) and fixed with 4% (w/v) paraformaldehyde in PBS for 20 min at room temperature. For detection of endogenous LC3B (Figures 7B and S7C), cells were fixed in ice cold methanol for 20 min on ice. For the immunofluorescent labelings in Figure 7, cells were permeabilized in 0.1% Triton X-100 in PBS for 5 min at room temperature and incubated for 1 h at room temperature in blocking buffer (1% BSA in PBS). Subsequently, coverslips were transferred into a humid chamber and incubated with primary antibody (rabbit anti-p62 1:500 – BD Bioscience, mouse anti-ubiquitin FK2 1:1000 – Enzo Life Science, mouse anti-LC3B 1:100 – nanoTools, rabbit anti-GABARAP 1:200 – Cell Signaling) diluted in blocking buffer for 1 h at room temperature. Following three PBS washing steps, coverslips were incubated, in the dark, with the secondary antibody (goat anti-mouse Alexa Fluor 647 1:500 – Jackson Immunoresearch, goat anti-rabbit Alexa Fluor 488 1:500 - Invitrogen). Coverslips were washed 3 times with PBS and mounted on glass slides (Roth) by inverting them onto a droplet of the mounting media DAPI-Fluoromont-G (Southern Biotech).

For the remaining immunofluorescence labeling, cells were permeabilized in 0.25% Triton X-100 for 15 min at room temperature. After two washes in PBS, coverslips were incubated for 30 min at room temperature in blocking buffer (1% BSA in PBS), transferred into a humid chamber and incubated with primary antibodies diluted in 1% BSA in PBS for 1 h at 37°C (FIP200-p62 immunostaining) or 16 h at 4°C (rabbit anti-FIP200 1:200 – Cell Signaling, mouse anti-p62 1:200 – BD Bioscience, rabbit anti-ULK1 1:50 – Santa Cruz

Biotech, rabbit anti-ATG16L1 1:50 – Cell Signaling). Coverslips were then washed 3 times for 5 min in PBS and incubated with secondary antibodies (goat anti-mouse Alexa Fluor 488 1:1000 – Invitrogen, goat anti-rabbit Alexa Fluor 647 1:500 – Jackson Immonoresearch). After 3 × 5 min washes in PBS, coverslips were mounted on glass slides using DAPI Fluoromont-G (Southern Biotech).

Imaging was performed on an inverted confocal laser scanning microscope Zeiss LSM 700, Plan-Apochromat 63x/1.4 Oil DIC or Plan-Apochromat 40x/1.3 Oil DIC. To prevent cross-contamination between fluorochromes, each channel was imaged sequentially using the multitrack recording module before merging. Images from fluorescence and confocal acquisitions were processed and analyzed with ImageJ software.

Cell lysis and western blotting

Unless otherwise specified, cells were harvested with trypsine. Cell pellets were washed with PBS and resuspended in 20 mM Tris-HCl pH 8.0, 10% glycerol, 135 mM NaCl, 0.5% Nonided P-40 Substitute, 2.5 mM $MgCl_2$, DNase, cOmplete EDTA-free protease inhibitor cocktail (Roche). For the lysates in Figure S2C the following lysis buffer was used: 50 mM Tris-HCl pH 7.4, 1 mM EGTA, 1 mM EDTA, 1% Triton X-100, 0.27 M sucrose, 1 mM DTT, 1mM NaF, 20 mM beta-glycerophosphate, 1 mM Na-vanadate). After 20 min incubation on ice, lysates were cleared by centrifugation at 16,000xg for 5 min at 4°C and total protein concentration was measured by Bradford protein assay (Bio-Rad). For subsequent western blot analysis, 20-25 μg of lysates were boiled for 5 min at 98°C resolved on SDS-PAGE and transferred on nitrocellulose membrane by wet blot. For the detection of LC3B I and II, samples were heated at 60°C for 10 min and proteins transferred on PVDF membrane. Membranes were blocked in 3% Non-fat dry Milk in TBS + 0.05% Tween-20 (blocking buffer) and incubated with primary antibody diluted in blocking buffer. After 3 × 15 min washes in TBS + 0.05% Tween-20 (TBST), they were incubated with secondary antibody conjugated with Horse Radish Peroxidase. After 3 × 15 min washes in TBST, membranes were developed using Super Signal West Pico chemiluminescence substrate. Images were taken with ChemiDoc Touch system (Bio-Rad) or films.

Antibody dilutions, unless otherwise specified: rabbit anti-phospho p62 S349 (1:1000, Cell Signaling); mouse anti-p62 (1:3000, BD-Bioscience); mouse anti-GAPDH (1:25000, Sigma); rabbit anti-FIP200 (1:1000, Cell Signaling – for the detection of full-length FIP200); mouse anti-FIP200 (1:100, MFPL antibody facility – for the detection of FIP200ΔClaw); mouse anti-LC3B (1:500 nanoTools).

Affinity co-purification of FIP200 and p62 from HAP1 FIP200-STG cells

Cells were washed 3x with PBS, harvested by Cell Lifter (Corning Incorporated, Costar) in ice-cold lysis buffer (50 mM Tris pH 7.5, 150 mM NaCl, 1 mM DTT and cOmplete EDTA-free protease inhibitor cocktail (Roche)), homogenized by resuspension with a 20-gauge syringe needle, and centrifuged at 13,000 g at 4°C for 15 min. Protein concentrations in the lysates were estimated using the DC protein assay kitII (Bio-Rad) according to manufacturer's protocol.

For affinity co-purification, 10 μl of StrepTactin Sepharose HP beads (GE Healthcare) were blocked with 5% BSA in lysis buffer for 1 h at 4°C. Then, lysates (total protein > 1 mg) were added to the beads and incubated for 2 h at 4°C. After 2 washes with lysis buffer, beads bound proteins were eluted by boiling the sample for 5 min at 98°C in Laemmli buffer and analyzed by SDS-PAGE followed by western blot. The membrane was probed with mouse anti-p62 (1:3000 – BD Bioscience). Affinity purification of FIP200-STG followed by quantitative mass spectrometry analysis were done using HAP1 cells grown in suspension. HAP1 wt and FIP200-STG cells were harvested by centrifugation at 1300xg for 15 min at 4°C and washed 3 times with PBS. Washed pellets were frozen in liquid nitrogen, resuspended in ice-cold lysis buffer (50 mM Tris pH 7.5, 150 mM NaCl, 1mM DTT and cOmplete EDTA-free protease inhibitor cocktail (Roche)) and lysed as described above for adherent cells. For immunoprecipitation 20 μl of StrepTactin Sepharose HP beads (GE Healthcare) were incubated with 4 mg of lysate for 2 h at 4°C. Beads were washed four times with lysis buffer and beads bound proteins were digested with trypsin for mass spectrometry analysis. Peptides were analyzed by a LC-MS/MS, and acquired spectra were searched against an *in- silico* digested protein database consisting of the human proteome (https://www.uniprot.org/) and common contaminants using the MaxQuant software. Proteins were relatively quantified across the samples using the label-free quantitation (LFQ) algorithm of the MaxQuant software. For ratio calculation, LFQ intensities were log_2-transformed and missing values were replaced with a fixed value (17.2).

HA-FIP200 – p62 co-immunoprecipitation from HeLa cells

HeLa cells were seeded in 10 cm dishes and let grow until 80% confluency. Cells were transfected with pME18s vectors (Addgene) containing HA-FIP200 (wt, point mutants or truncation) using Fugene transfection reagent (Promega). A vector:Fugene ratio of 1:6 was used for transfection. 24 h after transfection, cells were harvested with trypsin. The cell pellets were washed in PBS and resuspended in 100 μl lysis buffer (20 mM HEPES pH 7.5, 250 mM Sorbitol, 0.5 mM EGTA, 5 mM Mg-Acetate, 0.3 mM DTT, cOmplete EDTA-free protease inhibitor cocktail (Roche)). After 1 freeze and thaw cycle, the lysates were clarified by spinning at 1,000xg for 10 min at 4°C. Protein concentration in the lysates was measured by Bradford assay (BioRad) and all samples were adjusted to the same final concentration in 300 μl IP buffer (25 mM HEPES pH 7.5, 125 mM NaCl, 0.05% Triton X-100). Anti-HA magnetic beads (Pierce – Thermo Scientific) were washed 3 times in IP buffer and 1,5 μl of beads slurry were incubated with each sample for 1 h at 4°C on rotating wheel (9 rpm). After 3 × 5 min washes in IP buffer, beads were resuspended in 10 μl of 2x non-reducing protein loading dye and heated for 10 min at 95°C.

Samples were analyzed by western blot with mouse anti-p62 (1:500 – BD Bioscience) and rabbit anti-FIP200 (1:1000 – Atlas antibodies).

Cell treatment with siRNA

Cells were seeded in a 6 well plate (80,000 cells/well). Cells to be analyzed by immunocytochemistry were seeded on coverslips. The next day cells were transfected with siRNA (20 nM final concentration) using Lipofectamine RNAiMAX Transfection Reagent (Thermo Fisher) in OptiMEM medium. 48 h after transfection cells were either harvested for western blot analysis or fixed for immunofluorescence staining.

Co-immunoprecipitation of yeast proteins

S. cerevisiae Atg11Δ cells were transformed with pRS315-9myc-Atg11 plasmids in one-step buffer (0.24 M LiAc, 47% PEG4000) + 10 mM DTT, 5 μl of salmon sperm DNA and 1 μl of plasmid DNA. Cells were grown in YPD medium and 150 OD of cells were harvested by centrifugation at 3200xg for 8 min at room temperature. Cell pellets were washed in 10 mL PBS + 2% glucose and resuspended in 300 μl RLB+ buffer (10% glycerol, 0.5% Tween-20, 1 mM NaF, 20 mM beta-glycerophosphate, 1 mM PMSF, 1 mM vanadate, cOmplete EDTA-free protease inhibitor cocktail (Roche) in PBS). Cells were lysed with glass beads at 4°C (1 min beads beating, 1 min on ice, for 10 times). Lysates were cleared by centrifugation at 5,000xg for 2 min at 4°C and protein concentration was measured by Bradford protein assay (Bio-Rad) and adjusted to the same final concentration. 7.5 μl of anti-myc conjugated Dynabeads M-270 Epoxy (ThermoFisher) were added to each lysate and incubated for 1 h at 4°C on turning wheel. Beads were washed 3 times in 1 mL RLB+ and resuspended in 15 μl RLB+. Beads bound protein were eluted by boiling the beads for 10 min at 98°C in Urea loading buffer (116 mM Tris-HCl pH 6.8, 4.9% glycerol, 8 M Urea, 8% SDS) and analyzed by western blot with mouse anti-myc (1:500 – Millipore) and rabbit anti-Atg19 (1:7000) antibodies.

Ape1 processing assay

For this assay, *S. cerevisiae* BY4741 and Atg11Δ yeast strains were used. The Atg11Δ strain was transformed either with pRS315 empty vector or pRS315-Atg11 (wt or ΔCTR). Pre-cultures were grown in selective medium (SD-Leu) and used to inoculated O/N cultures in YPD. The next day, part of the cells were grown in starvation medium (SD-N) for additional 4 h at 30°C. Cells were harvested by centrifugation and whole cell lysates were prepared by trichloroacetic acid (TCA) extraction. Cell lysates were analyzed by western blot with rabbit anti-Ape1 (1:20000). Mouse anti-Atg11 (1:1000 – MFPL antibody facility) was used to test the expression level of Atg11 in the strains and conditions used.

QUANTIFICATION AND STATISTICAL ANALYSIS

Microscopy based protein-protein interaction assays

Quantification of all microscopy based protein-protein interaction assays was performed in ImageJ 1.x (Schneider et al., 2012) by drawing a line across each bead and taking the maximum gray value along the line. The maximum gray value for any given pixel represents the fluorescence intensity. The range of possible gray values for a given image depend on the color depth acquisition settings (0-255 for 8-bit images; 0-4065 for 12-bit images etc.). The average values for each sample were averaged between 3 independent replicates and plotted with the relative standard errors.

p62 pull-down experiments and protease protection assay

Protein bands intensities (Figures 3B, S3D, S6C, S6E, and S6F) were quantified with ImageJ by drawing a rectangle around the gel lane and obtaining the lane profile. The area of the peak in the profile was taken as a measure of the band intensity. Average intensities (normalized as described in the figure legend) and standard error of 3 independent experiments were plotted.

p62/ubiquitin puncta count and p62/LC3B colocalization analysis

p62 and ubiquitin puncta (Figures 7A and 7D) were counted using ImageJ. Images were thresholded and the accuracy of the established threshold was validated manually. The same threshold value was applied to all the images and replicates of the same experiment. Puncta were counted using the "Analyze Particles" function and excluding all particles smaller than 0.05 μ^2. The average number of puncta/cell was averaged between 3 or more independent experiments and standard errors were calculated. Using the "Analyze Particles" function the total area of p62 condensates was also measured and the average area of one particle was calculated. Assuming that the condensates have a spherical shape, the average particle volume could be extrapolated.

For all the colocalization analysis images were thresholded. Puncta in the green channel were identified with the "Analyze Particles" function as described above and the coordinates saved in the ROI. Then, puncta in the red channel were identified with the same method and overlayed in the ROI. The two sets of coordinates were visualized in different colors in the same image and the overlapping particles were counted. The average number of colocalizing puncta/cell was averaged between 2 or more independent experiments and standard errors were calculated.

Statistical analysis

For all the quantifications described above, statistical analysis was performed. Statistical significance of the difference between 2 samples was established by 2 samples unpaired t test. Significant differences are indicated with * when p value $\leq$ 0.05, ** when p value $\leq$ 0.01, *** when p value $\leq$ 0.001

DATA AND SOFTWARE AVAILABILITY

The FIP200 CTR and FIP200 Claw crystal structure data have been deposited in the PDB database with the ID codes PDB: 6GMA and PDB: 6DCE, respectively.

The original data for this study have been deposited in Mendeley Dataset and are available at the following link: https://doi.org/10.17632/jdv2yxymc5.1.

Molecular Cell, Volume *74*

Supplemental Information

FIP200 Claw Domain Binding to p62 Promotes Autophagosome Formation at Ubiquitin Condensates

Eleonora Turco, Marie Witt, Christine Abert, Tobias Bock-Bierbaum, Ming-Yuan Su, Riccardo Trapannone, Martin Sztacho, Alberto Danieli, Xiaoshan Shi, Gabriele Zaffagnini, Annamaria Gamper, Martina Schuschnig, Dorotea Fracchiolla, Daniel Bernklau, Julia Romanov, Markus Hartl, James H. Hurley, Oliver Daumke, and Sascha Martens

Figure S1 - related to Fig. 1

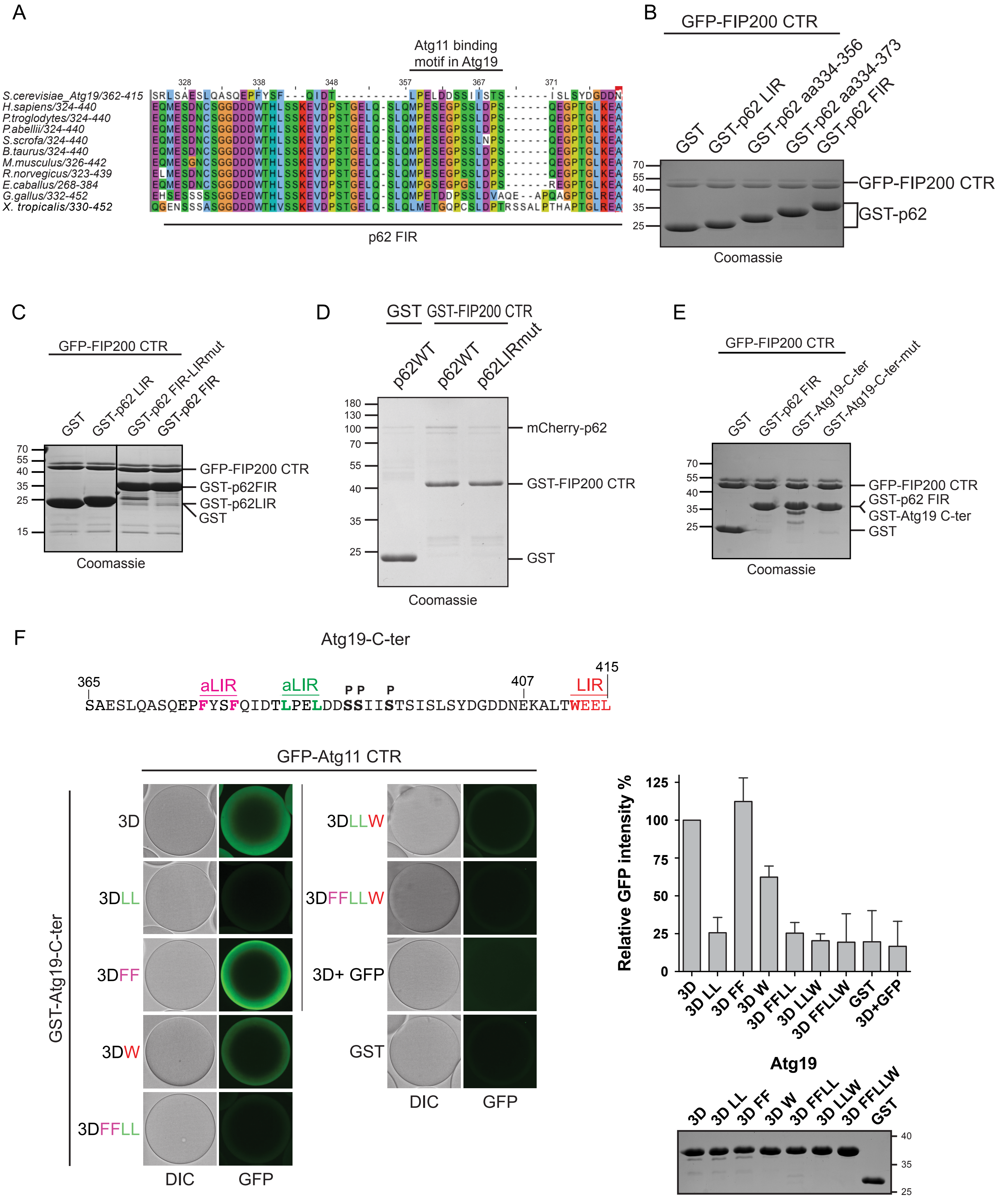

Figure S1, related to Figure 1.

A) Sequence alignment of p62 FIR from different species with *S. cerevisiae* Atg19. The region of p62 showing sequence similarity to the Atg11 binding site in Atg19 (aa 358-370) is indicated above the alignment.

B,C,D,E) Protein inputs for the respective experiments in Fig. 1E, F, G, H. 20 µl of each sample (beads + supernatant) were collected after microscopy imaging and analyzed by SDS-PAGE followed by Coomassie staining.

F) The Atg19 C-terminus sequence is shown. The canonical LIR motif at the extreme C-terminus is labeled in red. Pink and green labels indicate two accessory LIR-like motifs: $_{376}$FYSF$_{379}$ and $_{384}$LPEL$_{387}$. Phosphorylation sites are indicated in bold. The GFP-Atg11 CTR (aa851-1178) was mixed at 1µM final concentration with glutathione beads pre-coated with GST-Atg19 C-terminus (the phosphorylation-mimicking 3D mutant: S390D, S391D, S394D (Pfaffenwimmer et al., 2014) with the indicated LIR mutations (LL: L384, L386A, FF: F376, F379A, W: W412A) and the beads were imaged using a Zeiss LSM700 confocal microscope. The graph on the right shows average intensities (normalized to the signal of GFP-Atg11 CTR bound to GST-Atg19 C-ter 3D) and standard deviation of 3 independent experiments. The gel below the graph shows beads bound GST/GST Atg19 C-terminus constructs (1µl of each beads sample was loaded on the gel).

Figure S2 - related to Fig. 2

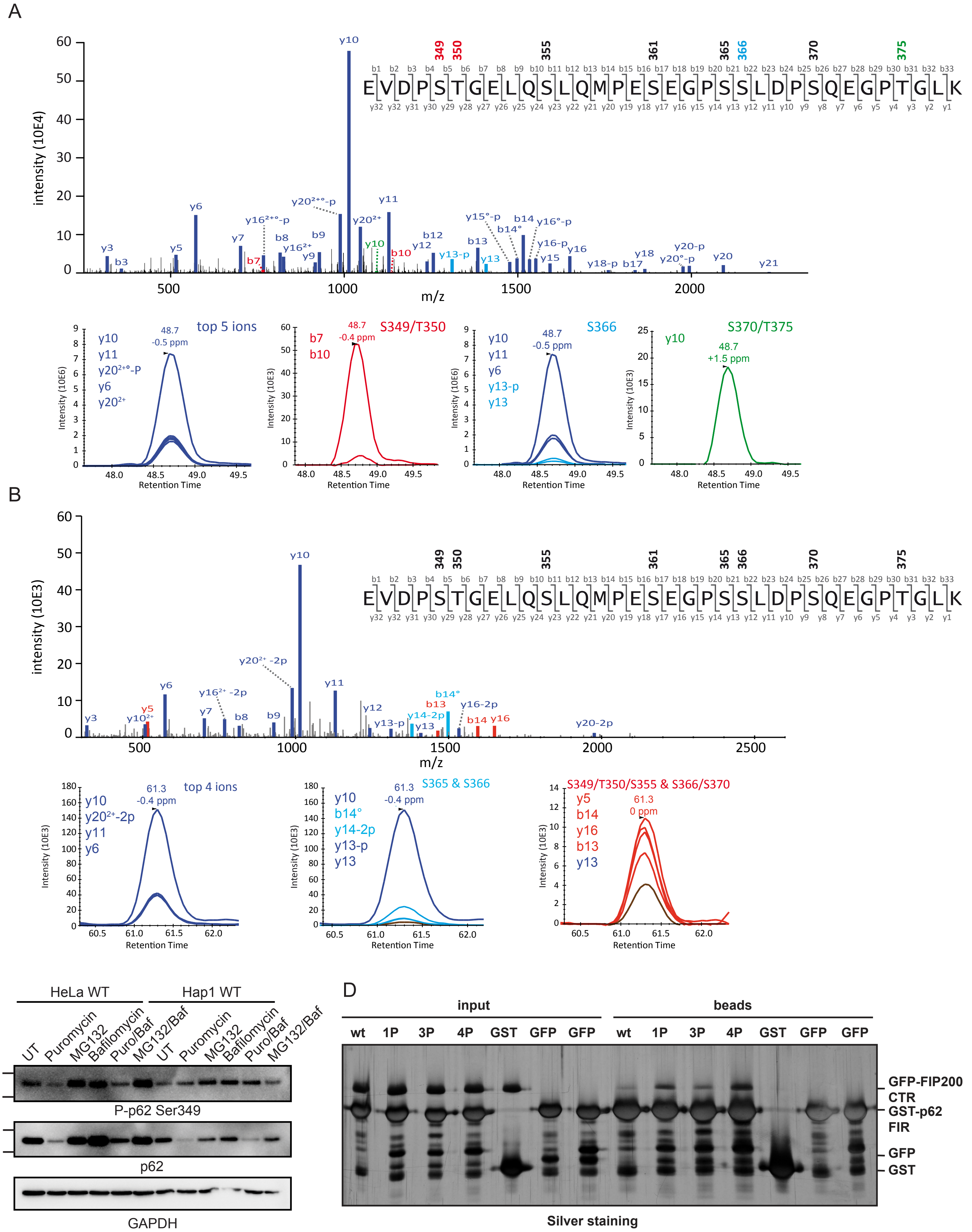

Figure S2, related to Figure 2.

A, B) MS/MS fragmentation spectra of singly (A) and doubly (B) phosphorylated forms of the peptide covering the FIR of p62. Spectra were acquired using parallel reaction monitoring, targeting peptide forms with up to four phosphorylations. Since all singly phosphorylated variants co-eluted, the spectra obtained display mixed signatures of different phosphorylation sites. Although this makes an unambiguous designation for some of the potential sites impossible (S355, S361), a combined analysis with the data of the doubly phosphorylated peptide allowed a clear assignment for two sites (S365, S366). Additionally, there is evidence for phosphorylation of at least one or both of the neighboring sites at the positions S349/T350, as well as at S370/S375. Depicted spectra were summed over the whole chromatographic peak, ions are color-coded for site-specific and -unspecific fragment ions, and the corresponding ion chromatograms are shown below each spectrum. Selected ions were validated manually in terms of mass accuracy, co-elution, isotopic envelope, and charge state. Dark blue: fragment ions with ambiguous site information; light blue, red, green: site-specific fragment ions; p: neutral phosphate loss; °: water loss.

C) HeLa and HAP1 cells were treated with puromycin, MG132, Bafilomycin or a combination of them for 3 h and cell lysates were analyzed by Western blot. The membranes were probed with a phospho-S349 specific antibody (top) or with an anti-p62 antibody (middle). An anti-GAPDH antibody was used as a loading control (bottom).

D) Binding of GFP-FIP200 CTR to GST-p62 FIR phospho-mimicking mutant assessed by GST pull-down. The same beads analyzed by microscopy in Fig. 2B were loaded on the gel before being washed (inputs) and after the unbound protein was washed away (beads).

Figure S3 - related to Fig. 2 and 3

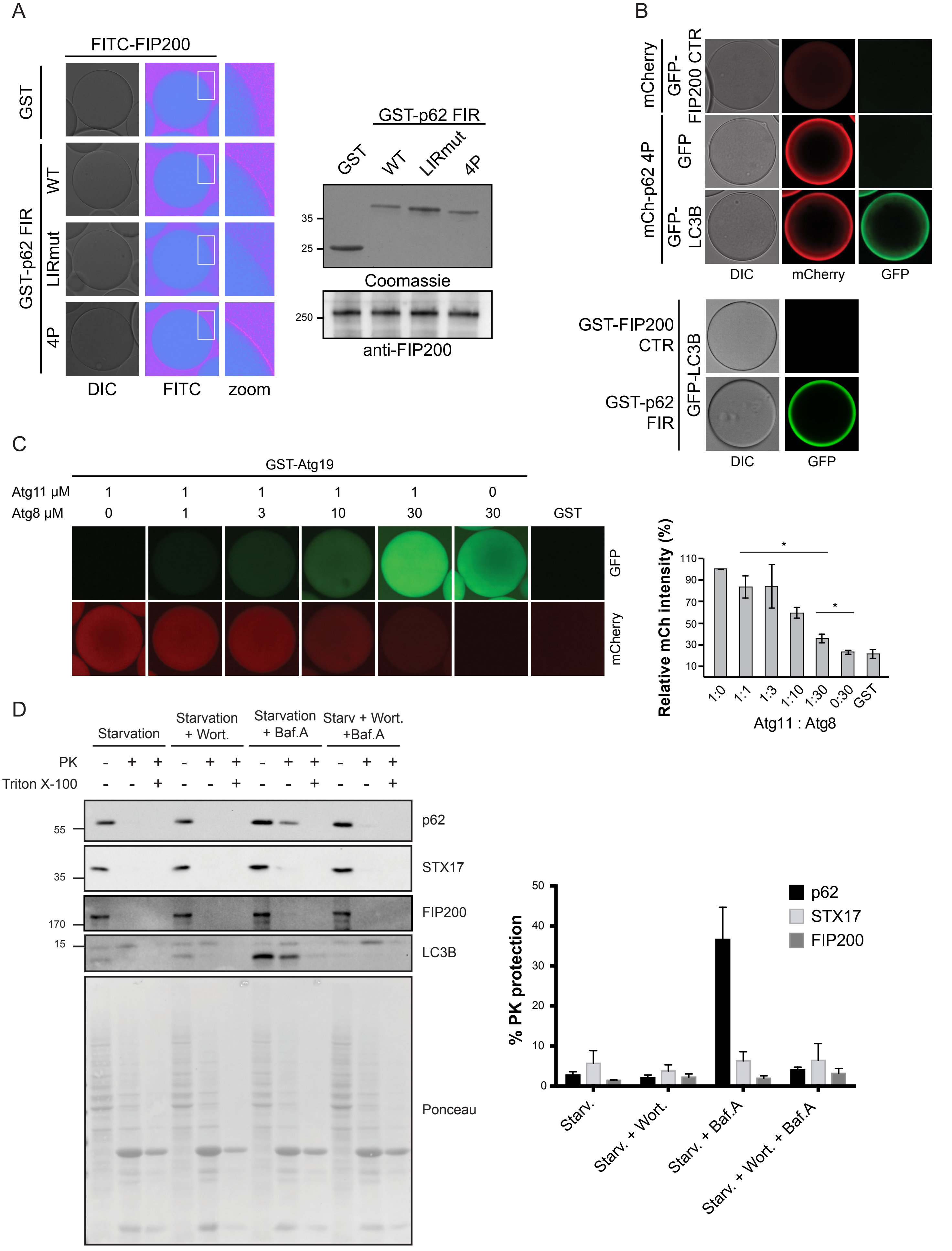

Figure S3, related to Figure 2 and 3.

A) Binding of GST-p62 FIR (wt, LIRmut and 4P phospho-mimetic mutant) to FITC-labelled full length FIP200 was assessed with the same experimental set up as in Fig. 2B. FITC peptide signal is shown in false color (ImageJ: Ice). The amount of bait protein on the beads was visualized by Coomassie staining and FIP200 protein inputs by Western blotting.

B) Negative and positive controls for the experiment in Fig. 3A. In the top panel, mCherry coated beads were incubated with GFP-FIP200 CTR (1.1 μM; top) and GST-p62 FIR 4P coated beads were incubated with GFP only (1.1 μM; middle) or GFP-LC3B (1.1 μM; bottom). In the bottom panel, GST-FIP200 CTR coated beads and GST-p62 FIR coated beads were incubated with GFP-LC3B (2 μM).

C) GST- Atg19 C-terminus coated glutathione beads were pre-incubated with mCherry-tagged Atg11 CTR and increasing amounts of GFP-Atg8 were added. Concentrations of mCherry-Atg11 CTR and GFP-Atg8 are indicated above the images. For the GST negative control, 1 μM mCherry-Atg11 CTR and 1 μM GFP-Atg8 were added. Average mCherry intensity (normalized to the intensity of 1:0 Atg11:Atg8 sample) and standard error of 3 independent experiments are plotted on the right. Significant differences are indicated with * when p value % 0.05, ** when p value % 0.01, and *** when p value % 0.001.

D) Protease protection assay as in Fig. 3B. As additional control, cells were treated with the PI3K inhibitor Wortmannin, which prevents autophagosome formation.

Figure S4 - related to Fig. 4

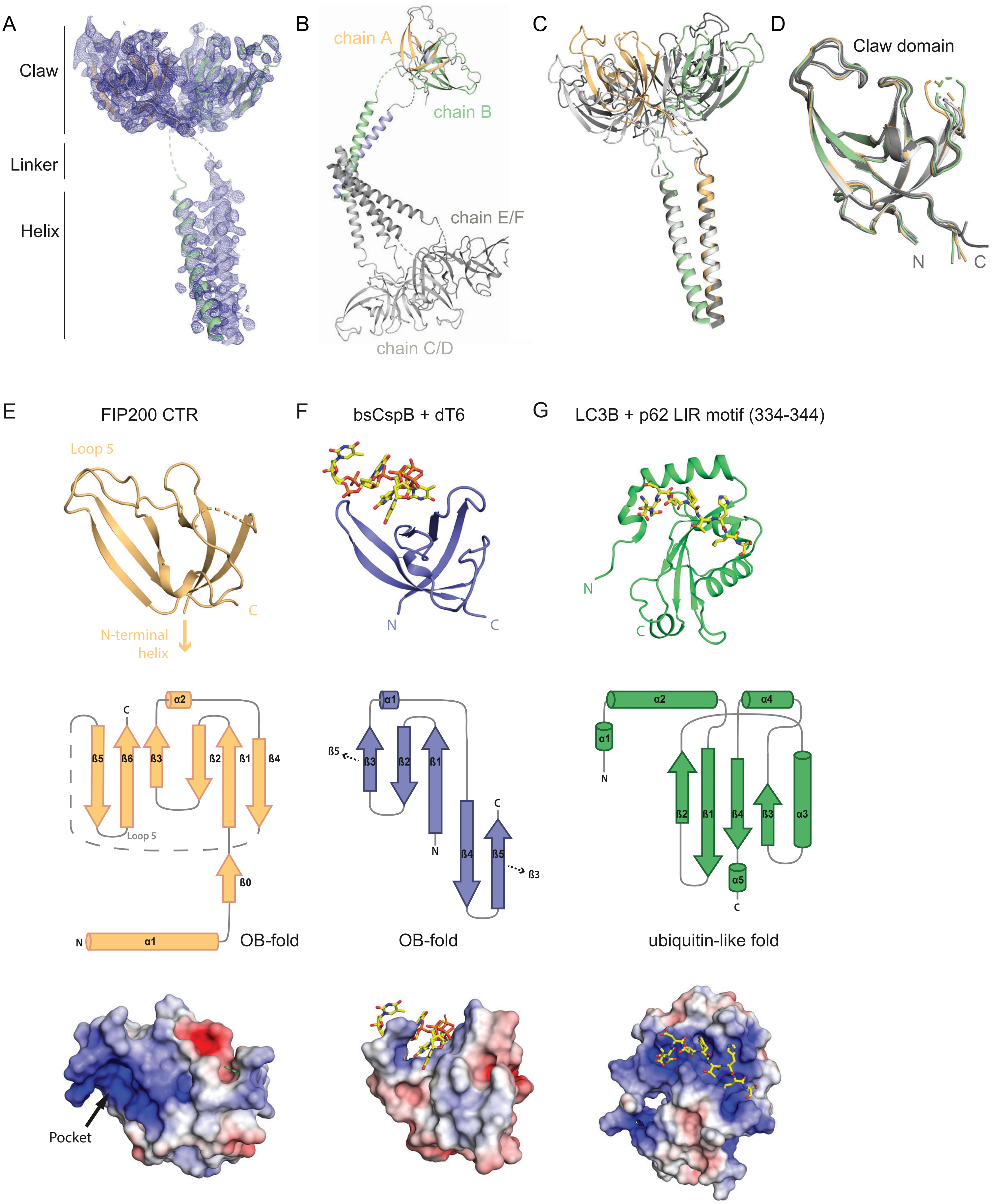

Figure S4, related to Figure 4.

A) Experimental electron density after density modification at 1 σ. Final model shown in cartoon representation.

B) Asymmetric unit cell of the crystal containing six molecules arranged as three dimers. One dimer is shown in colors.

C) Cα atoms superposition of the three dimers of the asymmetric unit cell (orange/green, light gray, dark gray). Only N-terminal α-helices were used for alignment.

D) Superposition of the Cα atoms in the six Claw domains present in the asymmetric unit cell (rmsd = 0.33).

E-G) Comparison of FIP200 Claw domain with *bs*CspB and LC3B. Cartoon representation, topology plot and electrostatic surface potential of E) FIP200 Claw domain, F) bsCspB bound to dT_7. (PDB code: 2ES2) and G) LC3B bound to the $p62^{334-344}$ LIR motif (PDB code: 2ZJD). Electrostatic surface potential in ±5 k_bT/e_c. Ligands are depicted in yellow stick representation. The arrow in S4E indicates the putative p62 FIR binding pocket.

Figure S5 - related to Fig. 4 and 5

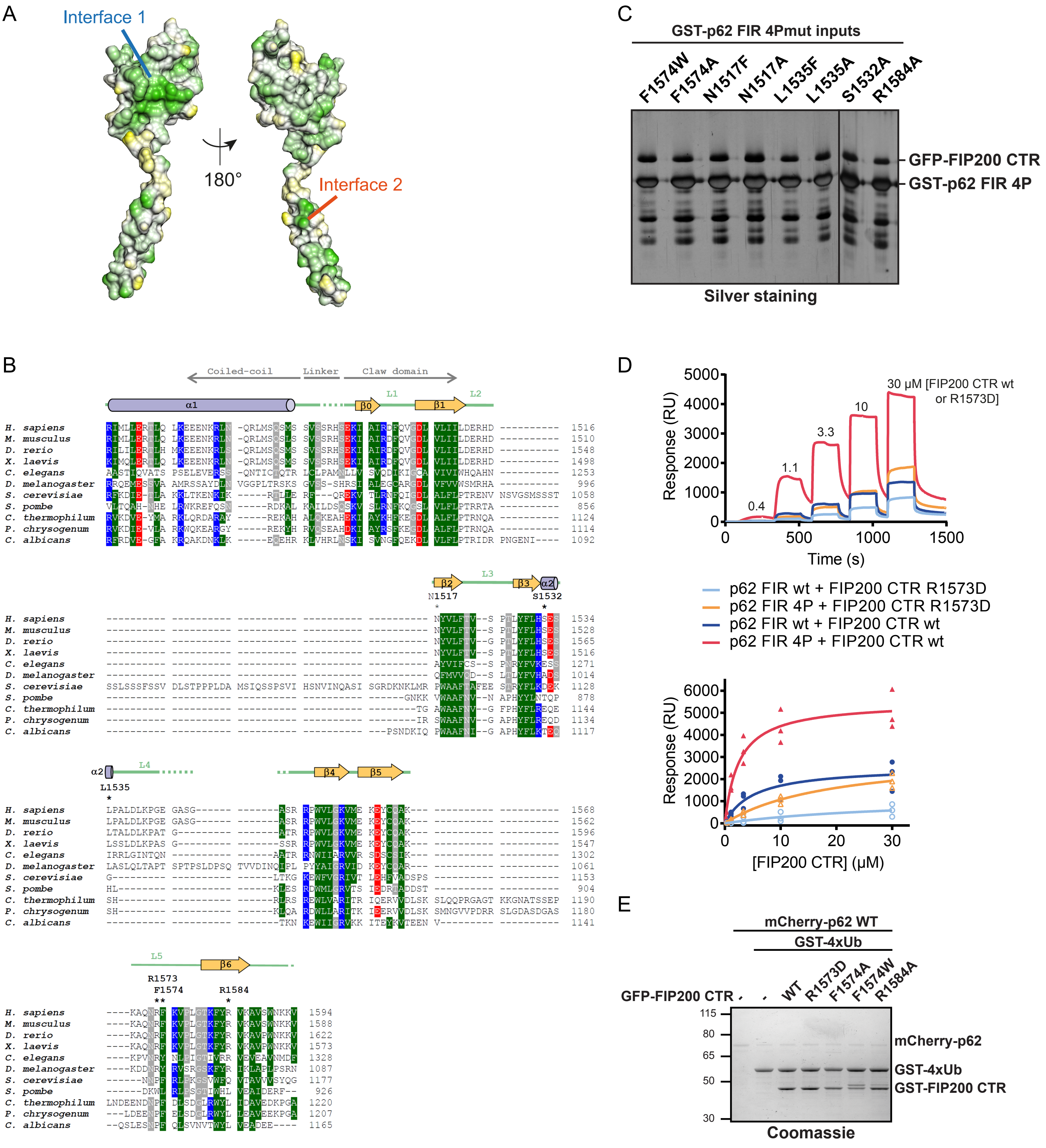

Figure S5, related to Figure 4 and 5.

A) Lipophilic surface potential of the FIP200 CTR monomer. Lipophilic and hydrophilic residues are colored in green and yellow, respectively.

B) Sequence alignment of the following Atg11 homology regions from different species (UniProt accession code in parentheses). *H. sapiens* (Q8TDY2), *M. musculus*, (Q9ESK9), *D. rerio*, (E7FFM2), *X. laevis* (A0A1L8FYZ6), *C. elegans* (Q22342), *D. melanogaster* (Q7KTS2), *S. cerevisiae* (Q12527), *S. pombe* (O14261), *C. thermophilum*, (G0S0K0), *P. chrysogenum* (A0A167XQU9), *C. albicans* (Q5AMN3). Residues with a conservation greater than 60% are color-coded (D, E in red; R, K, H in blue N, Q, S, T, G, P in gray; A, L, I, V, F, Y, W, M, C in green). FIP200 CTR secondary structure is displayed on top of the alignment. α-Helices are shown as cylinders and β-strands as arrows. Mutated residues in this study are indicated with an asterisk (*).

C) The samples (beads + supernatant) of the experiment in Fig. 5B were analyzed by SDS-PAGE followed by Silver staining.

D) Representative SPR sensorgrams (top) corrected for background binding to GST and buffer control. The experiment was performed as described in Fig. 2E to analyze the binding of GFP-FIP200 CTR wt and R1573D to GST-p62 FIR wt and 4P. Equilibrium analysis (bottom) for the FIP200 CTR variants: GST-p62 FIR variants interaction was performed as in Fig. 2F. Binding of the FIP200 CTR R1573D was too weak to be accurately fitted for a $K_D$app determination. Data were collected as two technical replicates of three independent experiments.

E) Loading control for the experiment in Fig. 5D. After imaging, samples were recovered and analyzed by SDS-PAGE followed by Coomassie staining.

Figure S6 - related to Fig. 6

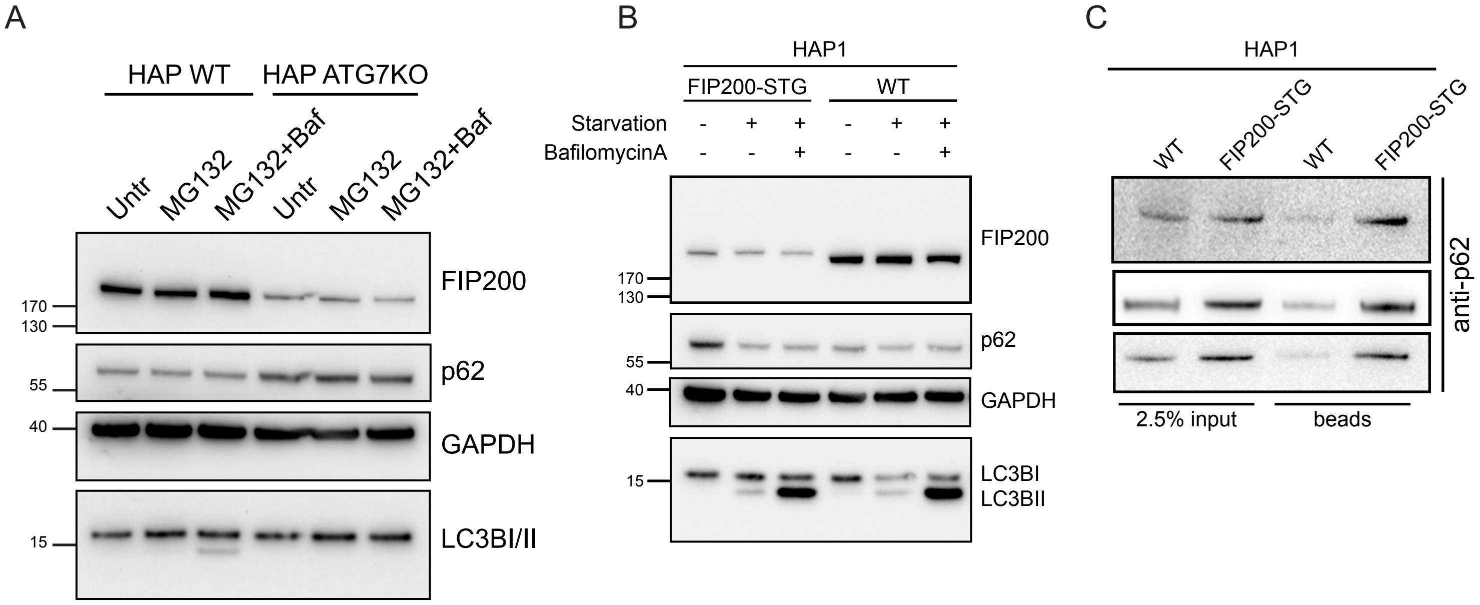

Figure S6, related to Figure 6

A) HAP1 wt or ATG7ko were left untreated or treated with MG132 (+/- Bafilomycin). After 1 h treatment, cell lysates were analyzed by Western blot to assess the ability to perform LC3B lipidation. FIP200 and p62 levels in the cells were also analyzed while GAPDH signal was used as loading control.

B) Validation of the HAP1 FIP200-STG cell line. Bulk autophagy was induced by starvation (in presence or absence of Bafilomycin) in HAP1 wt and FIP200-STG cell lines. Cell lysates were analyzed by Western blotting with anti-FIP200, anti-p62 and anti-LC3B. Anti-GAPDH was used as loading control. Overexpression of FIP200 caused cell death in the cell lines used.

C) 3 additional replicates of the affinity co-purification in Fig. 6C are shown.

Figure S7 - related to Fig. 7

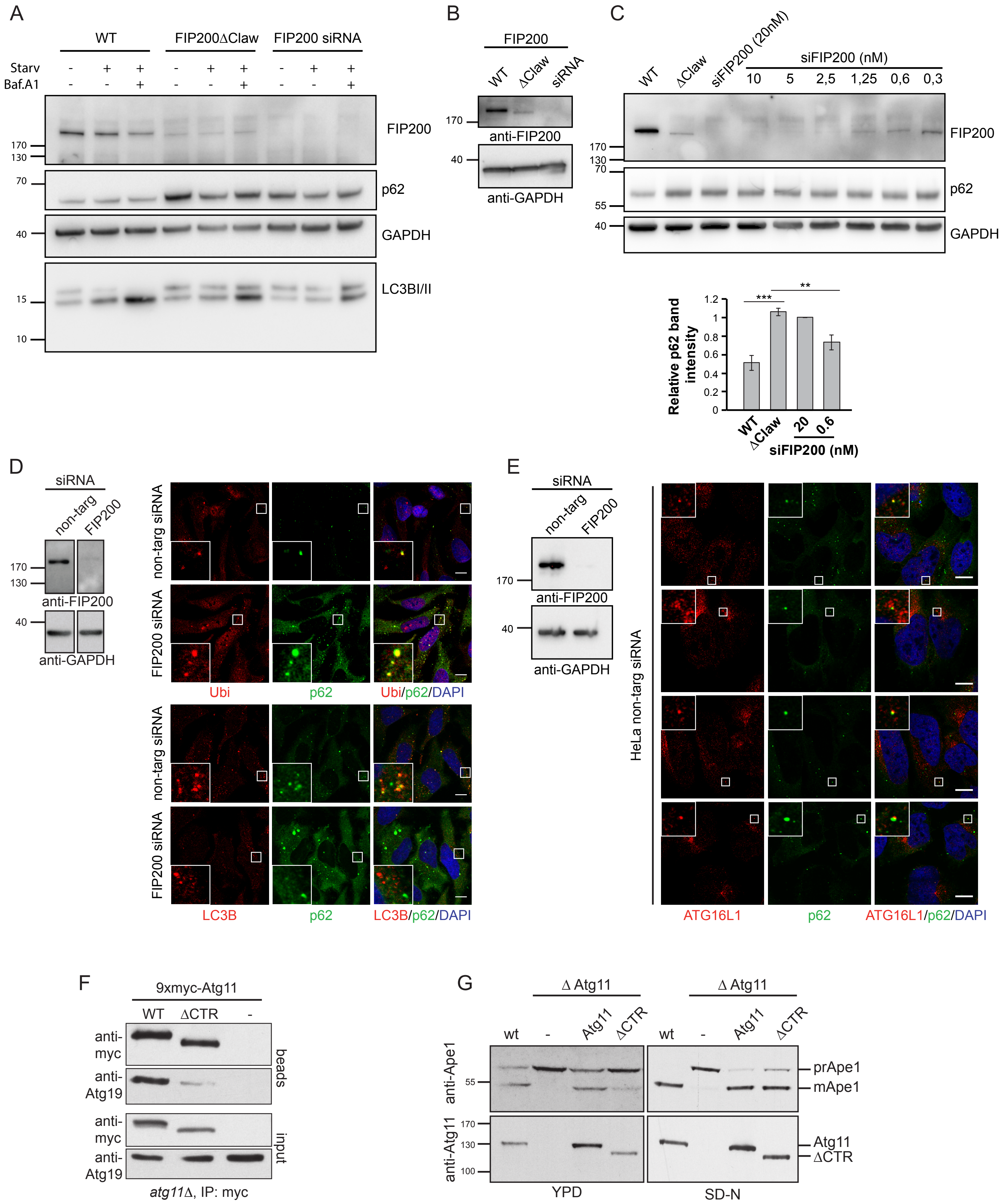

Figure S7, related to Figure 7.

A) HeLa wt and FIP200ΔClaw, treated with non-targeting siRNA and HeLa wt treated with FIP200 siRNA were left untreated or starved (in presence or absence of Bafilomycin). Cell lysates were analyzed by Western blotting for autophagy markers (p62 accumulation and LC3B lipidation) and FIP200. GAPDH levels were used as loading control.

B) Expression levels of FIP200 wt and FIP200ΔClaw in HeLa cells were analyzed by Western blotting using lysates collected from low-passage number (3) cells.

C) Higher passage number HeLa FIP200ΔClaw were treated with non-targeting siRNA and wt cells were treated either with non-targeting siRNA or the indicated concentrations of FIP200 siRNA. Then, FIP200 expression levels and p62 levels were analyzed by Western blotting. p62 band intensity for the samples shown in the plot was measured with ImageJ and normalized to the amount of GAPDH in the lysate. Average p62 band intensity (relative to p62 levels in HeLa treated with 20 nM FIP200 siRNA) and standard error for 4 independent experiments are shown. Significant differences are indicated with * when p value % 0.05, ** when p value % 0.01, and *** when p value % 0.001.

D) Immunofluorescence staining with ubiquitin and p62 or p62 and LC3B relative to the quantification showed in Fig. 7D. HeLa cells treated with non-targeting siRNA or FIP200 siRNA were fixed and immuno-stained as indicated. Scale bar = 10 µm. The efficiency of the siRNA treatment was assessed by Western blotting.

E) Cells used for the immunofluorescence staining in Fig. 7E were analyzed by Western blotting with anti-FIP200 to assess the efficiency of the siRNA treatment. Additional images of p62-ATG16L1 immunofluorescence in HeLa cells treated with non-targeting siRNA are shown. Scale bar = 10 µm.

E) Co-immunoprecipitation of *S. cerevisiae* Atg11 and Atg19. An Atg11-deficient yeast strain was transformed with plasmids expressing myc-Atg11 (full-length or ΔCTR, under endogenous promoter) or empty plasmid. Atg11 was immunoprecipitated using the myc tag and samples were probed with Atg19 antibody.

F) prApe1 processing assay using Atg11 deficient yeast strains transformed with the indicated Atg11 constructs. Cells were grown in YPD (rich) or SD-N (starvation) medium and cell lysates were analyzed by Western blotting. Conversion of prApe1 into mApe1 was used to monitor the selective Cvt pathway.

Table S1-related to Figure 6C: Mass Spectrometry analysis of FIP200-STG purified from Hap1 cells.

Gene Name	Protein Name	Score	Sequence coverage (%)	Log2(LFQ) (STG-FIP200-ST-GFP/wt)
**RB1CC1**	FIP200/RB1-inducible coiled-coil protein 1	323,3	68,5	13,07
**ULK1**	Serine/threonine-protein kinase ULK1	323,3	51,7	12,41
**ATG13**	Autophagy-related protein 13	323,3	54,4	9,9
**ATG101**	Autophagy-related protein 101	267,8	73,9	11,96
**SQSTM1**	p62/Sequestosome-1	227,1	38,2	1,68

Table S1. FIP200-STG and wt Hap1 cells were subjected to StrepTactin purification followed by mass spectrometry. Listed are the known FIP200 direct interactors enriched in the FIP200-STG sample compared to the wt. A Log2(LFQ)≥1 was considered as significant enrichment. LFQ: label-free quantitation.
